# Sociodemographic characteristics associated with hepatitis C virus infection in Vietnamese Americans: A cross-sectional analysis of community screening data

**DOI:** 10.1371/journal.pone.0275210

**Published:** 2022-09-27

**Authors:** Alice W. Lee, Wura Jacobs, Michelle Tran, Becky Nguyen, Dung N. Hua, John N. Ho, Thai Van Nguyen

**Affiliations:** 1 Department of Public Health, California State University, Fullerton, Fullerton, CA, United States of America; 2 Department of Kinesiology, California State University, Stanislaus, Turlock, CA, United States of America; 3 Vietnamese American Cancer Foundation, Fountain Valley, CA, United States of America; Affiliated Hospital of Nantong University, CHINA

## Abstract

**Background:**

Prevalence of hepatitis C virus (HCV) infection among Vietnamese Americans is reportedly high. Understanding the profile of those at greater risk of HCV in this ethnic population is a vital step to addressing this high prevalence. We hypothesize that certain sociodemographic characteristics increase the likelihood of having HCV in Vietnamese Americans.

**Methods:**

Cross-sectional data from 2,497 Vietnamese Americans in Southern California who participated in a series of community hepatitis screening events organized by the Vietnamese American Cancer Foundation (VACF) were analyzed. Serological tests via immunoassays were used to determine whether the participant had hepatitis C antibodies (anti-HCV) to indicate a HCV infection. Sociodemographic characteristics as well as participants’ reasons for screening were collected from questionnaires, and logistic regression models with odds ratios (ORs) and 95% confidence intervals (CIs) were used to quantify their associations with HCV infection.

**Results:**

Approximately 5.8% of the study population was infected with HCV. Older adults and male participants had higher odds of being infected with HCV (e.g. OR = 2.90, 95% CI 1.25–6.76 for ages 70+ versus ages <40; OR = 2.57, 95% CI 1.79–3.69 for male versus female participants) as were those with a family history of HCV infection (OR = 2.74, 95% CI 1.57–4.78). In addition, perceived self-risk as a motivation for screening was significantly associated with HCV infection (OR = 1.88, 95% CI 1.26–2.78).

**Conclusions:**

This study identifies specific subgroups in the Vietnamese American community who would largely benefit from targeted interventions given their higher likelihood of having HCV. These interventions should emphasize improving HCV knowledge and promoting HCV self-risk assessment since awareness of one’s own risk may motivate those likely to be infected to get screened.

## Introduction

Hepatitis C virus (HCV) is associated with both acute and chronic liver disease, which can result in liver cirrhosis and liver cancer [1]. It is a major public health issue with important behavioral implications since reports estimate 50% to 80% of individuals are unaware of their HCV infection status [2–4]. In the United States (U.S.), more than two million individuals have a chronic HCV infection [3, 5], and global estimates indicate this number to be more than 170 million people, the majority of whom come from the Western Pacific and Southeast Asia regions [6–8].

Vietnamese Americans constitute one of the largest Asian ethnic groups and account for a significant number of HCV cases with a disease prevalence that mirrors the reported prevalence in Vietnam [7]. Although the prevalence of HCV in the general U.S. population is estimated to be only about 0.84% [5], studies have shown it to range from 2.2% to 15.4% among Vietnamese Americans [7, 9–13]. This higher prevalence has been attributed to several behavioral factors prior to their immigration to the U.S., including exposure to unsanitary medical practices during childhood as well as use of contaminated needles during blood transfusions, acupuncture, or traditional tattooing [12]. There was also increased immigration after the Vietnam War, which may explain why existing studies have found the 1945–1965 birth cohort to be at higher risk for HCV [14–17] and why older age may be a predictor of HCV infection [7, 9, 12, 15].

As more Vietnamese migrate to the U.S. from HCV endemic countries, identifying those at higher risk of HCV is an essential first step to addressing the disease’s burden in this community as they could inform future targeted strategies. In addition, despite the importance of screening and early detection, prevalence of HCV screening among Vietnamese Americans is sub-optimal [18], hence understanding how screening motivations is associated with HCV infection may be relevant as well. This study leverages serological and self-reported questionnaire data collected from a series of community hepatitis screening events to examine factors associated with HCV infection among Vietnamese Americans in Southern California.

## Materials and methods

### Study design and population

Cross-sectional data were collected from free community hepatitis screening events organized by the Vietnamese American Cancer Foundation (VACF) (http://www.vacf.org/) between February 2011 and November 2017. VACF is a public-benefit organization located in Fountain Valley, California that serves Southern California residents, especially Vietnamese Americans, through cancer education, research, advocacy, and services. Their events are promoted through announcements on local Vietnamese radio and television stations and newspapers as well as through community referral.

All participants of VACF’s events who desired screening were tested for infection with HCV. Because some participated in multiple screening events, duplicates were excluded as such individuals are likely to be quite different from those who only attended once. This resulted in 3,264 unique participants (i.e. those who participated in only one event). We also excluded those who did not report a Vietnamese ethnicity (N = 757) nor a Southern California residence (N = 9). After these exclusions, one participant was excluded due to an indeterminate HCV serological test result, resulting in a final sample size of 2,497 Vietnamese Americans.

### Data collection

At each screening event, blood was drawn by trained phlebotomists and transported to various commercial laboratories in the Orange County, California region that partner with VACF. Using immunoassays for hepatitis serology, each participant’s blood was tested for hepatitis C antibodies (anti-HCV). Prior to phlebotomy, all participants signed a written consent form available in English and Vietnamese. Trained VACF staff also gave consenting participants a questionnaire that included questions about their ethnicity and language preference, demographics, personal and family health history relating to HCV, and reasons for getting screened. Further details regarding data ascertainment have been previously published [19]. An institutional ethics review board approved the retrospective analysis of the de-identified VACF community hepatitis screening event data used in this study (research project number: HSR-17-18-515).

### Measures

Individuals with a positive blood test for anti-HCV were classified as having an HCV infection. Those with a negative test result were classified as not having an HCV infection. Basic sociodemographic information that was collected via the questionnaire included sex (male, female), age (<40, 40–49, 50–59, 60–69, and 70+ years), marital status (single/widowed, married), highest level of education completed (less than high school, high school graduate, some college or technical/vocational training, college graduate), and annual household income (<$10,000, $10,001-$30,000, $30,001-$50,000, $50,001+). Other characteristics included whether the participant was born in the U.S. (yes, no), their current employment status (employed, unemployed) as well as whether they had health insurance (yes, no). Participants’ family history of HCV was determined from a series of questions about whether they had an infected family member and which specific member of the family was infected. These questions were combined into a binary variable where “yes” indicated having one or more family members infected with HCV and “no” indicated having no infected family member.

Those who participated in VACF’s hepatitis screening events from 2011 through 2016 were asked to indicate why they were getting screened on the questionnaire, and each participant could select multiple reasons; questions related to reason for screening were not asked of participants in 2017. Three overarching reasons were considered for this analysis: advised by a family member or friend, recommended by a health care provider, and perceived self-risk (e.g. thought they were infected, has an infected family member). These three reasons were considered because not only were they likely to reflect participants’ risk of HCV, but they also captured participants’ risk from different perspectives.

### Data analysis

Descriptive analyses based on HCV infection status were conducted, and data were summarized as percentages. Chi-square tests and Fisher’s exact tests were used to examine the association between each sociodemographic characteristic in categories and HCV infection. Characteristics that were statistically significantly different between those infected with HCV and those uninfected at a p-value ≤0.10 [20] in addition to those considered relevant *a priori* (i.e. age, sex, education level, U.S. birth status) were included in a multivariate logistic regression model to quantify each characteristic’s association with HCV infection using odds ratios (ORs) and 95% confidence intervals (CIs). To evaluate the association between reason for screening participation and HCV infection, multivariate logistic regression analyses were conducted for each of the three reasons separately after adjusting for the *a priori* characteristics; among the 2011–2016 participants since reason for screening was not asked of participants in 2017, only those who reported their reason for screening were included (N = 2,045).

To ensure our selection of significant factors to be included in the multivariate logistic regression model was not driven by differences in the missing categories, the Chi-square tests and Fisher’s exact tests were conducted excluding the missing. In the multivariate logistic regression analyses, those missing covariate data were modeled with missing categories to preserve the sample size. Sensitivity analyses were conducted using a complete case approach, but since the overall findings did not change, the results with the missing categories are presented.

All statistical analyses were performed using SAS software, release 9.4 (SAS Institute Inc., Cary, NC). Statistical significance was considered at a p-value of ≤0.05 and all tests of statistical significance were two-sided.

## Results

A total of 3,264 unique individuals participated in VACF’s screening events from February 2011 to November 2017. Of them, 2,497 were Vietnamese Americans living in Southern California who had HCV serological data, constituting the final sample used for analysis. Overall, 5.8% (N = 146) of the included participants were infected with HCV.

Participants’ characteristics are shown in Table 1. Those uninfected with HCV were younger than those infected; 20.5% of uninfected participants were <40 years of age versus only 6.9% of infected participants (Table 1). In addition, there was a significant difference in the distribution of sex with most HCV cases being among male participants (p<0.001; Table 1). There was also a higher percentage of infected participants with a lower annual household income (p = 0.053) and a family history of HCV (p = 0.002) as well as who were currently married (p = 0.005) and unemployed (p = 0.012) when compared to those uninfected with HCV (Table 1). Across all participants, the majority were born outside the U.S. (95.5%) and had health insurance (59.6%) (Table 1).

**Table 1:** Characteristics of study participants by HCV infection status.

Characteristic	Uninfected (N = 2,351)			Infected (N = 146)			Total (N = 2,497)			p-value^c^
	N	% across^a^	% within^b^	N	% across^a^	% within^b^	N	% across^a^	% within^b^	
Age										<0.001
<40 years	481	20.5	98.0	10	6.9	2.0	491	19.7	100.0	
40–49 years	527	22.4	96.3	20	13.7	3.7	547	21.9	100.0	
50–59 years	664	28.2	92.0	58	39.7	8.0	722	28.9	100.0	
60–69 years	475	20.2	92.2	40	27.4	7.8	515	20.6	100.0	
70+ years	201	8.6	91.8	18	12.3	8.2	219	8.8	100.0	
Missing	3	0.1	100.0	0	0.0	0.0	3	0.1	100.0	
Sex										<0.001
Female	1328	56.6	96.2	53	36.3	3.8	1381	55.3	100.0	
Male	1013	43.1	91.6	93	63.7	8.4	1106	44.3	100.0	
Missing	10	0.4	100.0	0	0.0	0.0	10	0.4	100.0	
Highest Education Completed										0.053
Less than high school	461	19.6	92.9	35	24.0	7.1	496	19.9	100.0	
High school graduate	775	33.0	92.9	59	40.4	7.1	834	33.4	100.0	
Some college or technical/vocational training	420	17.9	95.5	20	13.7	4.5	440	17.6	100.0	
College graduate	528	22.5	95.8	23	15.8	4.2	551	22.1	100.0	
Missing	167	7.1	94.9	9	6.2	5.1	176	7.1	100.0	
Annual Household Income										0.069
<$10,000	705	30.0	92.9	54	37.0	7.1	759	30.4	100.0	
$10,000-$30,000	789	33.6	94.5	46	31.5	5.5	835	33.4	100.0	
$30,001-$50,000	241	10.3	92.3	20	13.7	7.7	261	10.5	100.0	
$50,001+	143	6.1	97.9	3	2.1	2.1	146	5.9	100.0	
Missing	473	20.1	95.4	23	15.8	4.6	496	19.9	100.0	
Marital Status										0.005
Married	1485	63.2	93.2	108	74.0	6.8	1593	63.8	100.0	
Single/widowed	741	31.5	96.1	30	20.6	3.9	771	30.9	100.0	
Missing	125	5.3	92.5	8	5.5	6.0	133	5.3	100.0	
Employment Status										0.012
Unemployed	1136	48.3	93.1	84	57.5	6.9	1220	48.9	100.0	
Employed	1015	43.2	95.6	47	32.2	4.4	1062	42.5	100.0	
Missing	200	8.5	93.0	15	10.3	7.0	215	8.6	100.0	
Health Insurance Status										0.54
Uninsured	705	30.0	94.1	44	30.1	5.9	749	30.0	100.0	
Insured	1409	59.9	94.8	78	53.4	5.2	1487	59.6	100.0	
Missing	237	10.1	90.8	24	16.4	9.2	261	10.5	100.0	
Family History of HCV										0.002
No	1321	56.2	94.4	78	53.4	5.6	1399	56.0	100.0	
Yes	141	6.0	88.1	19	13.0	11.9	160	6.4	100.0	
Missing	889	37.8	94.8	49	33.6	5.2	938	37.6	100.0	
Born in the United States										0.071
No	2240	95.3	94.0	144	98.6	6.0	2384	95.5	100.0	
Yes	80	3.4	98.8	1	0.7	1.2	81	3.2	100.0	
Missing	31	1.3	96.9	1	0.7	3.1	32	1.3	100.0	

HCV, hepatitis C virus.

^a^ Percentage of participants across the subgroups for each characteristic.

^b^ Percentage of participants within each subgroup for each characteristic.

^c^ Based on Chi-square or Fisher’s exact tests comparing those uninfected to those infected with HCV excluding those missing for each characteristic.

Table 2 presents the ORs and 95% CIs from a multivariate regression model for HCV infection. Compared to participants ages <40 years, those ages 50–59, 60–69, and 70+ had three times the odds of being infected with HCV (OR = 3.36, 95% CI 1.63–6.93, OR = 3.26, 95% CI 1.55–6.86, and OR = 2.90, 95% CI 1.25–6.76, respectively; Table 2). Male participants as well as those with a family history of HCV also had over twice the odds of being infected (OR = 2.57, 95% CI 1.79–3.69 and OR = 2.74, 95% CI 1.57–4.78, respectively; Table 2). Characteristics that appeared to be associated with lower odds of HCV infection included a higher level of education (OR = 0.62, p = 0.10 for college graduates), having employment (OR = 0.68, p = 0.075), being single/widowed (OR = 0.77, p = 0.25), and being born in the U.S. (OR = 0.51, p = 0.52) although none of these results were statistically significant (Table 2).

**Table 2:** Multivariate logistic regression analysis of characteristics and their associations with HCV infection.

Characteristic	OR^a^	95% CI	p-value
Age			
<40 years	1.00	--	--
40–49 years	1.60	0.71–3.59	0.26
50–59 years	3.36	1.63–6.93	0.001
60–69 years	3.26	1.55–6.86	0.002
70+ years	2.90	1.25–6.76	0.014
Missing	N/A	N/A	N/A
Sex			
Female	1.00	--	--
Male	2.57	1.79–3.69	<0.001
Missing	N/A	N/A	N/A
Highest Education Completed			
Less than high school	1.00	--	--
High school graduate	1.07	0.68–1.66	0.78
Some college or technical/vocational training	0.64	0.35–1.15	0.14
College graduate	0.62	0.35–1.09	0.10
Missing	0.85	0.39–1.87	0.69
Annual Household Income			
<$10,000	1.00	--	--
$10,000-$30,000	0.84	0.54–1.31	0.43
$30,001-$50,000	1.36	0.74–2.49	0.32
$50,001+	0.40	0.12–1.36	0.14
Missing	0.66	0.38–1.13	0.13
Marital Status			
Married	1.00	--	--
Single/widowed	0.77	0.49–1.20	0.25
Missing	0.96	0.44–2.06	
Employment Status			
Unemployed	1.00	--	--
Employed	0.68	0.44–1.04	0.075
Missing	1.23	0.67–2.27	0.51
Family History of HCV			
No	1.00	--	--
Yes	2.74	1.57–4.78	<0.001
Missing	1.01	0.70–1.48	0.94
Born in the United States			
No	1.00	--	--
Yes	0.51	0.07–3.99	0.52
Missing	0.57	0.07–4.41	0.59

HCV, hepatitis C virus; OR, odds ratio; CI, confidence interval.

^a^ Model adjusted for all other characteristics included in the table. N/A indicates cannot be calculated due to a zero cell.

Across the three reasons for screening considered, perception of self-risk was the most frequent selection (22.3%) (Table 3). In addition, after adjusting for age, sex, education level, and U.S. birth status in each model for each screening reason, perception of self-risk was the only reason statistically significantly associated with HCV infection; those who were getting screened because they felt they were at risk of HCV had 1.88 times the odds of being infected with HCV (OR = 1.88, 95% CI 1.26–2.78; Table 3). Participants who were getting screened based on the recommendation of a family member, friend, or health care provider were not statistically significantly more likely to be infected (Table 3).

**Table 3:** Association between screening motivations and HCV infection among only participants who reported their reason for screening.

Reason for Screening^a^	Uninfected^b^ (N = 1,920)	Infected^b^ (N = 125)	Total^b^ (N = 2,045)	OR^c^	95% CI	p-value
Advised by family member or friend						
Not selected	1,693 (88.2)	118 (94.4)	1,811 (88.6)	1.00	--	--
Selected	227 (11.8)	7 (5.6)	234 (11.4)	0.51	0.23–1.11	0.087
Recommended by health care provider						
Not selected	1,775 (92.4)	117 (93.6)	1,892 (92.5)	1.00	--	--
Selected	145 (7.6)	8 (6.4)	153 (7.5)	0.88	0.42–1.86	0.74
Perceived self-risk						
Not selected	1,506 (78.4)	83 (66.4)	1,589 (77.7)	1.00	--	--
Selected	414 (21.6)	42 (33.6)	456 (22.3)	1.88	1.26–2.78	0.002

HCV, hepatitis C virus; OR, odds ratio; CI, confidence interval.

^a^ “Selected” indicates those who selected this reason as a reason for getting screened. “Not selected” indicates those who did not select this reason as a reason for getting screened.

^b^ Number of participants (% of participants).

^c^ Each reason for screening was modeled separately and adjusted for age, sex, education level, and whether the participant was born in the United States.

## Discussion

Prevalence of HCV infection among Vietnamese Americans is high [7], yet there are few HCV studies that focus specifically on this ethnic population. To address this gap, our study leveraged data from a well-established community-based organization’s hepatitis screening events to identify characteristics that are associated with HCV infection among Vietnamese Americans. Overall, we found several notable differences by sex, age, and family history of HCV. We also found perception of self-risk as a reason for screening to be statistically significantly associated with HCV infection.

Despite our study being based solely on Vietnamese Americans in the Southern California region, the 5.8% HCV prevalence we determined is in line with previous studies, which reported prevalence ranging from 2.2% to 15.4% [7, 9–13]. Although HCV prevalence in our study was on the lower end of the range, it remains notably higher than the average prevalence in the general U.S. population (~0.84%) [5], highlighting how the Vietnamese American community remains disproportionately burdened by HCV. Furthermore, given that Southern California is home to the highest concentration of Vietnamese Americans nationally [21] and therefore likely sees significant migration from Vietnam, a high endemic region for HCV, the number of HCV cases is sure to be quite substantial, making this geographic area relevant for this work.

Identifying factors associated with HCV infection is critical as this information can inform strategies for HCV screening and awareness. Similar to earlier studies that found older adults, particularly those born between 1945 and 1965, to be most at risk for HCV [7, 9, 12, 15], our study also observed those older to have higher risk. As mentioned previously, such participants likely immigrated to the U.S. after the Vietnam War, and because Vietnam is a developing country with high HCV prevalence, older adults may have been more likely to contract HCV through exposure to unsanitary medical practices and other behaviors that may have involved the use of contaminated needles prior to their immigration [7, 22, 23]. This would also explain the lower odds of HCV we observed among Vietnamese Americans born in the U.S. although this result was not statistically significant.

We found male participants to be at a higher risk of HCV infection than female participants, and this finding likely reflects behavioral factors given that male participants may be less health conscious and more likely to engage in high-risk activities [24], such as injection drug use which is a major HCV risk factor [25]. Having a family history of HCV significantly increased the odds of having HCV as well, which is consistent with prior research [26, 27]. HCV, like many diseases, has environmental and lifestyle antecedents that family members share, hence an individual’s family history has the potential to capture information about shared factors that contribute to HCV risk [28]. There was also some suggestion of education and employment being associated with HCV, and this may be related to health literacy, which could reflect one’s understanding of HCV [29–31]. Overall, these findings highlight specific subgroups in the Vietnamese American community who can benefit from targeted health campaigns.

A unique aspect to our study is the evaluation of screening motivations and their association with HCV infection in Vietnamese Americans. The majority of participants in our study reported receiving screening services due to their perception of self-risk (22.3%), and this reason for screening significantly increased the odds of being infected with HCV by 88%. Self-risk perception (i.e., beliefs about potential harm to oneself) is likely driven by both an individual’s understanding of HCV risk factors in relation to their own behaviors as well as their environment, such as observing infected members of their family or social network [32–34]. Given that this self-awareness appears to be associated with whether someone is infected with HCV, interventions that aim to increase HCV knowledge may be especially important in this community. Few studies have evaluated levels of HCV knowledge in Vietnamese Americans although the promotion and development of HCV educational programs has been shown to improve HCV knowledge in this population [31]. Future efforts should improve HCV knowledge in Vietnamese Americans as this could help identify those at high risk of the disease who, in turn, could be targeted when it comes to outreach for future screening events.

Our study is not without its limitations. First, it is a cross-sectional analysis of participants from one specific geographic region (i.e. Southern California), and although Southern California is home to the highest number of Vietnamese Americans in the U.S. [21], they still may not be representative of all Vietnamese Americans. Our study is also based on those who chose to be screened and such participants may be healthier or more health-conscious than the general Vietnamese American community. However, it should be noted that those who engage in high-risk behaviors may be equally likely to choose to participate in screening. In addition, the estimated HCV prevalence in our study is consistent with other community studies that are based on convenience samples. Second, because this study relied on a questionnaire to obtain information from each participant, there could be issues with recall. However, it is unlikely that those infected with HCV and those uninfected would differentially report the characteristics considered in this analysis. Third, the data used in this study were obtained from community screening events, and as such, questionnaires have to be brief due to time constraints. Therefore, information on several key risk factors and health details were not collected from the participants. And lastly, some participants were missing covariate data, and for our multivariate analyses, we used a missing indicator approach to preserve our sample size. Although such an approach could lead to biased estimates, a sensitivity analysis using a complete case approach resulted in similar findings. In addition, the results for the missing categories did not suggest missingness being associated with HCV.

Despite these limitations, our study makes important contributions to the body of work on HCV among Vietnamese Americans. Many hepatitis screening studies often target the general Asian American population with few focusing specifically on Vietnamese Americans [9] who are not only sociodemographically and culturally distinct from other Asian ethnicities, but also have the highest rates of liver cancer across all racial/ethnic groups [35]. To our knowledge, the most recent study on HCV in Vietnamese Americans was based on data from 2012, hence our study provides an updated evaluation of HCV and its prevalence for this ethnic population. Other strengths of our study are the ascertainment of participants’ HCV infection status using serology, which minimized disease misclassification, as well as its large sample size. Asian Americans constitute only 7% of the U.S. population [36], hence examining specific Asian ethnic subgroups (like Vietnamese Americans) can often result small numbers that may not be sufficient. In addition, no study to our knowledge has examined the association between screening motivations and HCV infection among Vietnamese Americans, and our finding of self-risk perception being statistically significantly associated with HCV infection highlights an important opportunity for targeted efforts.

Using a community-based effort, we have identified several characteristics associated with higher odds of HCV among Vietnamese Americans, including perception of self-risk which underscores the importance of HCV knowledge in personal risk assessment as this could motivate those infected to get screened. We have also highlighted HCV’s continued burden among Vietnamese Americans and the need for ongoing interventions in this community. These findings should be used to inform and improve current HCV strategies so they can more effectively serve Vietnamese Americans when it comes to this important public health issue.

## Supporting information

S1 Dataset (DOCX)
