# Supplementary material for: Sociodemographic characteristics associated with hepatitis C virus infection in Vietnamese Americans: A cross-sectional analysis of community screening data
Source: PLoS One. 2022 Sep 27;17(9):e0275210. doi: 10.1371/journal.pone.0275210 (PMC9514619; doi:10.1371/journal.pone.0275210)
Supplement: S1 Dataset — (DOCX) [file pone.0275210.s001.docx]

| Year | Sex  0=female  1=male  2=missing | Education  0=less than hs  1=hs grad  2=some coll  3=coll grad  4=missing | Income  0=<$10k  1=$10k-30k  2=$30,001-50k  3=$50,001+  4=missing | Health insurance  0=no  1=yes  2=missing | Marital status  0=married  1=single/ widowed  2=missing | Age  0=<40  1=40-49  2=50-59  3=60-69  4=70+  5=missing | Hep C infection  0=no  1=yes | Family history of hepatitis C  0=no  1=yes  2=missing | Born in US  0=no  1=yes  2=missing | Work  0=not employed  1=employed  2=missing | Doctor recomm to screen  0=no  1=yes  2=missing | Self-risk as reason to screen  0=no  1=yes  2=missing | Family or friend recomm to screen  0=no  1=yes  2=missing |
| --- | --- | --- | --- | --- | --- | --- | --- | --- | --- | --- | --- | --- | --- |
| 2013 | 1 | 3 | 0 | 0 | 1 | 3 | 0 | 0 | 0 | 0 | 0 | 0 | 0 |
| 2013 | 1 | 1 | 4 | 0 | 0 | 1 | 0 | 0 | 0 | 1 | 0 | 0 | 0 |
| 2013 | 1 | 2 | 1 | 0 | 0 | 2 | 0 | 0 | 0 | 1 | 1 | 0 | 0 |
| 2013 | 1 | 1 | 4 | 2 | 0 | 1 | 0 | 2 | 0 | 1 | 2 | 2 | 2 |
| 2013 | 0 | 1 | 1 | 0 | 1 | 1 | 0 | 0 | 0 | 1 | 0 | 0 | 0 |
| 2013 | 0 | 3 | 2 | 1 | 1 | 2 | 0 | 0 | 0 | 1 | 0 | 0 | 0 |
| 2013 | 1 | 4 | 2 | 2 | 0 | 2 | 0 | 0 | 0 | 1 | 1 | 0 | 0 |
| 2013 | 0 | 1 | 0 | 1 | 0 | 3 | 0 | 0 | 0 | 0 | 1 | 0 | 0 |
| 2013 | 1 | 2 | 1 | 1 | 1 | 0 | 0 | 2 | 0 | 1 | 1 | 0 | 0 |
| 2013 | 0 | 4 | 4 | 1 | 0 | 4 | 0 | 0 | 0 | 2 | 2 | 2 | 2 |
| 2013 | 0 | 1 | 1 | 1 | 0 | 0 | 0 | 0 | 0 | 1 | 1 | 0 | 0 |
| 2013 | 0 | 3 | 3 | 1 | 0 | 1 | 0 | 0 | 0 | 1 | 0 | 0 | 0 |
| 2013 | 1 | 4 | 4 | 1 | 0 | 4 | 0 | 0 | 0 | 2 | 2 | 2 | 2 |
| 2013 | 0 | 3 | 1 | 1 | 0 | 1 | 0 | 0 | 0 | 1 | 1 | 0 | 0 |
| 2013 | 0 | 0 | 0 | 1 | 1 | 1 | 0 | 0 | 0 | 0 | 0 | 0 | 0 |
| 2013 | 1 | 1 | 0 | 2 | 1 | 0 | 0 | 2 | 0 | 0 | 2 | 2 | 2 |
| 2013 | 0 | 2 | 2 | 0 | 0 | 0 | 0 | 2 | 0 | 1 | 1 | 0 | 0 |
| 2013 | 0 | 1 | 1 | 0 | 0 | 0 | 0 | 0 | 0 | 1 | 0 | 0 | 1 |
| 2013 | 0 | 3 | 1 | 0 | 0 | 1 | 0 | 0 | 0 | 0 | 0 | 0 | 1 |
| 2013 | 1 | 3 | 0 | 0 | 1 | 0 | 0 | 0 | 0 | 1 | 1 | 0 | 0 |
| 2013 | 0 | 3 | 1 | 0 | 0 | 2 | 0 | 0 | 0 | 1 | 0 | 0 | 0 |
| 2013 | 1 | 3 | 0 | 0 | 1 | 0 | 0 | 0 | 0 | 0 | 0 | 0 | 0 |
| 2013 | 0 | 1 | 1 | 0 | 1 | 2 | 0 | 0 | 0 | 0 | 0 | 0 | 0 |
| 2013 | 0 | 2 | 1 | 0 | 1 | 1 | 0 | 2 | 0 | 1 | 0 | 0 | 0 |
| 2013 | 0 | 0 | 2 | 0 | 0 | 1 | 0 | 0 | 0 | 1 | 1 | 0 | 0 |
| 2013 | 1 | 2 | 2 | 0 | 0 | 0 | 0 | 0 | 0 | 1 | 1 | 0 | 0 |
| 2013 | 1 | 3 | 1 | 0 | 0 | 1 | 0 | 0 | 0 | 1 | 0 | 0 | 0 |
| 2013 | 0 | 3 | 4 | 0 | 0 | 3 | 0 | 0 | 0 | 0 | 0 | 0 | 0 |
| 2013 | 1 | 2 | 2 | 0 | 0 | 1 | 0 | 0 | 0 | 1 | 0 | 0 | 1 |
| 2013 | 0 | 3 | 3 | 1 | 0 | 1 | 0 | 2 | 0 | 1 | 0 | 0 | 0 |
| 2013 | 0 | 3 | 4 | 2 | 0 | 0 | 0 | 2 | 0 | 0 | 2 | 2 | 2 |
| 2013 | 0 | 3 | 2 | 0 | 1 | 1 | 0 | 0 | 0 | 1 | 0 | 0 | 0 |
| 2013 | 0 | 1 | 1 | 0 | 0 | 2 | 0 | 0 | 0 | 0 | 0 | 0 | 0 |
| 2013 | 0 | 3 | 4 | 1 | 0 | 2 | 0 | 0 | 0 | 2 | 2 | 2 | 2 |
| 2013 | 0 | 2 | 4 | 1 | 0 | 0 | 0 | 0 | 0 | 0 | 0 | 0 | 0 |
| 2013 | 1 | 2 | 3 | 1 | 0 | 2 | 0 | 0 | 0 | 1 | 0 | 0 | 1 |
| 2013 | 0 | 3 | 4 | 2 | 0 | 1 | 0 | 0 | 0 | 0 | 1 | 0 | 0 |
| 2013 | 0 | 0 | 0 | 1 | 0 | 2 | 0 | 0 | 0 | 0 | 1 | 0 | 0 |
| 2013 | 0 | 1 | 4 | 1 | 0 | 3 | 0 | 0 | 0 | 1 | 1 | 0 | 0 |
| 2013 | 0 | 3 | 2 | 1 | 0 | 1 | 0 | 2 | 0 | 1 | 1 | 0 | 0 |
| 2013 | 0 | 0 | 0 | 0 | 0 | 3 | 0 | 0 | 0 | 0 | 1 | 0 | 0 |
| 2013 | 0 | 2 | 1 | 0 | 0 | 2 | 0 | 2 | 0 | 0 | 1 | 0 | 0 |
| 2013 | 0 | 3 | 4 | 2 | 1 | 0 | 0 | 0 | 0 | 0 | 1 | 0 | 0 |
| 2013 | 1 | 2 | 0 | 0 | 1 | 1 | 0 | 0 | 0 | 0 | 1 | 0 | 0 |
| 2013 | 0 | 2 | 0 | 1 | 1 | 0 | 0 | 2 | 1 | 0 | 1 | 0 | 0 |
| 2013 | 1 | 3 | 3 | 1 | 0 | 0 | 0 | 0 | 0 | 1 | 1 | 0 | 0 |
| 2013 | 0 | 1 | 4 | 2 | 0 | 0 | 0 | 2 | 0 | 0 | 2 | 2 | 2 |
| 2013 | 0 | 0 | 0 | 2 | 0 | 2 | 0 | 2 | 0 | 1 | 0 | 0 | 0 |
| 2013 | 0 | 2 | 0 | 0 | 1 | 0 | 0 | 0 | 0 | 1 | 0 | 0 | 0 |
| 2013 | 0 | 0 | 1 | 1 | 1 | 4 | 0 | 0 | 0 | 0 | 0 | 0 | 0 |
| 2013 | 1 | 1 | 0 | 1 | 1 | 0 | 0 | 0 | 0 | 1 | 0 | 0 | 0 |
| 2013 | 1 | 1 | 1 | 1 | 0 | 2 | 0 | 0 | 0 | 1 | 1 | 0 | 0 |
| 2013 | 0 | 1 | 2 | 0 | 2 | 1 | 0 | 2 | 0 | 2 | 1 | 0 | 0 |
| 2013 | 1 | 3 | 1 | 0 | 0 | 2 | 0 | 0 | 0 | 0 | 0 | 0 | 0 |
| 2013 | 0 | 0 | 0 | 0 | 2 | 4 | 0 | 2 | 0 | 0 | 0 | 0 | 0 |
| 2013 | 1 | 1 | 1 | 1 | 1 | 0 | 0 | 0 | 0 | 1 | 0 | 0 | 0 |
| 2013 | 1 | 1 | 0 | 0 | 2 | 0 | 0 | 0 | 0 | 2 | 1 | 0 | 0 |
| 2013 | 0 | 3 | 0 | 1 | 1 | 0 | 0 | 0 | 1 | 1 | 0 | 0 | 0 |
| 2013 | 0 | 3 | 1 | 0 | 0 | 1 | 0 | 0 | 0 | 1 | 2 | 2 | 2 |
| 2013 | 1 | 1 | 2 | 1 | 0 | 0 | 0 | 0 | 0 | 1 | 0 | 0 | 0 |
| 2013 | 1 | 2 | 1 | 1 | 0 | 3 | 0 | 0 | 0 | 1 | 1 | 0 | 0 |
| 2013 | 0 | 1 | 1 | 0 | 0 | 1 | 0 | 0 | 0 | 0 | 1 | 0 | 0 |
| 2013 | 0 | 2 | 1 | 2 | 1 | 0 | 0 | 2 | 0 | 1 | 1 | 0 | 0 |
| 2013 | 0 | 2 | 1 | 1 | 1 | 0 | 0 | 0 | 0 | 0 | 1 | 0 | 0 |
| 2013 | 0 | 2 | 1 | 0 | 1 | 0 | 0 | 0 | 0 | 1 | 0 | 0 | 0 |
| 2013 | 0 | 2 | 0 | 0 | 1 | 0 | 0 | 0 | 0 | 1 | 0 | 0 | 0 |
| 2013 | 0 | 3 | 1 | 1 | 0 | 4 | 0 | 0 | 0 | 0 | 0 | 1 | 0 |
| 2013 | 0 | 3 | 1 | 1 | 0 | 3 | 0 | 0 | 0 | 0 | 1 | 0 | 0 |
| 2013 | 1 | 1 | 1 | 0 | 1 | 0 | 0 | 1 | 0 | 0 | 1 | 0 | 0 |
| 2013 | 0 | 4 | 3 | 1 | 1 | 0 | 0 | 0 | 0 | 1 | 0 | 0 | 0 |
| 2013 | 1 | 0 | 4 | 1 | 1 | 0 | 0 | 2 | 0 | 0 | 1 | 0 | 0 |
| 2013 | 0 | 1 | 1 | 1 | 0 | 1 | 0 | 0 | 0 | 0 | 0 | 0 | 0 |
| 2013 | 1 | 0 | 1 | 2 | 0 | 1 | 0 | 2 | 0 | 1 | 0 | 0 | 0 |
| 2013 | 1 | 0 | 1 | 0 | 0 | 0 | 0 | 2 | 0 | 1 | 0 | 0 | 0 |
| 2013 | 0 | 1 | 1 | 1 | 0 | 2 | 0 | 0 | 0 | 1 | 1 | 0 | 0 |
| 2013 | 0 | 0 | 1 | 2 | 0 | 0 | 0 | 0 | 0 | 1 | 0 | 0 | 0 |
| 2013 | 0 | 1 | 4 | 2 | 1 | 0 | 0 | 0 | 0 | 0 | 1 | 0 | 0 |
| 2013 | 0 | 2 | 2 | 0 | 0 | 0 | 0 | 0 | 0 | 0 | 1 | 0 | 0 |
| 2013 | 0 | 0 | 1 | 0 | 1 | 3 | 0 | 0 | 0 | 1 | 0 | 0 | 0 |
| 2013 | 0 | 3 | 0 | 2 | 2 | 2 | 0 | 0 | 0 | 1 | 0 | 0 | 0 |
| 2013 | 0 | 1 | 0 | 1 | 1 | 4 | 0 | 0 | 0 | 1 | 1 | 0 | 0 |
| 2013 | 1 | 3 | 0 | 0 | 1 | 0 | 0 | 0 | 1 | 1 | 1 | 0 | 0 |
| 2013 | 1 | 3 | 4 | 0 | 1 | 0 | 0 | 0 | 0 | 0 | 1 | 0 | 0 |
| 2013 | 0 | 2 | 0 | 0 | 0 | 3 | 0 | 0 | 0 | 0 | 0 | 0 | 0 |
| 2013 | 0 | 2 | 2 | 0 | 0 | 3 | 0 | 0 | 0 | 1 | 0 | 0 | 0 |
| 2013 | 1 | 2 | 2 | 0 | 0 | 1 | 0 | 0 | 0 | 1 | 0 | 0 | 0 |
| 2013 | 1 | 2 | 1 | 1 | 0 | 4 | 0 | 2 | 0 | 0 | 1 | 0 | 0 |
| 2013 | 1 | 4 | 4 | 0 | 0 | 2 | 0 | 2 | 0 | 0 | 1 | 0 | 0 |
| 2013 | 1 | 0 | 4 | 1 | 2 | 4 | 0 | 0 | 0 | 2 | 0 | 0 | 0 |
| 2013 | 1 | 3 | 1 | 0 | 0 | 2 | 0 | 0 | 0 | 2 | 0 | 0 | 0 |
| 2013 | 1 | 1 | 1 | 0 | 1 | 0 | 0 | 2 | 0 | 1 | 0 | 0 | 0 |
| 2013 | 1 | 1 | 4 | 0 | 0 | 3 | 0 | 0 | 0 | 0 | 1 | 0 | 0 |
| 2013 | 0 | 1 | 0 | 1 | 1 | 3 | 0 | 2 | 0 | 0 | 0 | 0 | 0 |
| 2013 | 0 | 1 | 1 | 1 | 0 | 2 | 0 | 1 | 0 | 1 | 1 | 0 | 0 |
| 2013 | 0 | 3 | 1 | 0 | 2 | 2 | 0 | 0 | 0 | 0 | 0 | 0 | 0 |
| 2013 | 0 | 3 | 2 | 1 | 0 | 1 | 0 | 0 | 0 | 1 | 0 | 0 | 0 |
| 2013 | 1 | 3 | 0 | 0 | 2 | 1 | 0 | 0 | 0 | 1 | 1 | 0 | 0 |
| 2013 | 0 | 1 | 0 | 0 | 0 | 2 | 0 | 0 | 0 | 0 | 0 | 0 | 0 |
| 2013 | 0 | 0 | 2 | 0 | 0 | 1 | 0 | 0 | 0 | 1 | 0 | 0 | 0 |
| 2013 | 0 | 0 | 1 | 1 | 0 | 1 | 0 | 0 | 0 | 1 | 1 | 0 | 0 |
| 2013 | 0 | 1 | 1 | 2 | 1 | 2 | 0 | 0 | 0 | 1 | 0 | 0 | 0 |
| 2013 | 1 | 3 | 4 | 0 | 0 | 1 | 0 | 2 | 0 | 1 | 0 | 0 | 0 |
| 2013 | 0 | 1 | 4 | 0 | 0 | 2 | 0 | 2 | 0 | 0 | 0 | 0 | 0 |
| 2013 | 0 | 0 | 1 | 2 | 0 | 1 | 0 | 2 | 0 | 2 | 2 | 2 | 2 |
| 2013 | 1 | 0 | 0 | 2 | 2 | 2 | 0 | 2 | 0 | 2 | 2 | 2 | 2 |
| 2013 | 0 | 2 | 4 | 2 | 1 | 2 | 0 | 1 | 0 | 1 | 0 | 1 | 0 |
| 2013 | 0 | 0 | 0 | 1 | 2 | 0 | 0 | 0 | 0 | 0 | 0 | 0 | 0 |
| 2013 | 1 | 2 | 0 | 0 | 0 | 1 | 0 | 0 | 0 | 0 | 1 | 0 | 0 |
| 2013 | 0 | 1 | 4 | 1 | 0 | 2 | 0 | 1 | 0 | 0 | 1 | 0 | 0 |
| 2013 | 1 | 0 | 0 | 0 | 1 | 1 | 0 | 1 | 0 | 0 | 1 | 0 | 0 |
| 2013 | 0 | 2 | 0 | 1 | 1 | 3 | 0 | 0 | 0 | 0 | 1 | 0 | 0 |
| 2013 | 0 | 0 | 0 | 0 | 2 | 2 | 0 | 0 | 0 | 0 | 1 | 0 | 1 |
| 2013 | 0 | 3 | 2 | 1 | 1 | 1 | 0 | 0 | 0 | 1 | 0 | 0 | 0 |
| 2013 | 1 | 1 | 0 | 1 | 0 | 2 | 0 | 0 | 0 | 0 | 0 | 0 | 0 |
| 2013 | 1 | 1 | 1 | 1 | 0 | 1 | 0 | 0 | 0 | 1 | 1 | 0 | 0 |
| 2013 | 0 | 0 | 4 | 0 | 1 | 1 | 0 | 0 | 0 | 0 | 1 | 0 | 0 |
| 2013 | 0 | 1 | 1 | 1 | 1 | 2 | 0 | 0 | 0 | 1 | 1 | 0 | 0 |
| 2013 | 0 | 1 | 0 | 0 | 0 | 3 | 0 | 0 | 0 | 0 | 1 | 0 | 0 |
| 2013 | 0 | 1 | 0 | 0 | 0 | 1 | 0 | 0 | 0 | 1 | 0 | 0 | 0 |
| 2013 | 1 | 3 | 1 | 1 | 2 | 3 | 0 | 0 | 0 | 2 | 0 | 0 | 0 |
| 2013 | 0 | 0 | 2 | 0 | 0 | 3 | 0 | 0 | 0 | 2 | 0 | 0 | 0 |
| 2013 | 1 | 0 | 1 | 1 | 1 | 1 | 0 | 0 | 0 | 1 | 0 | 0 | 0 |
| 2013 | 0 | 0 | 1 | 0 | 0 | 2 | 0 | 0 | 0 | 1 | 0 | 0 | 0 |
| 2013 | 1 | 1 | 0 | 2 | 2 | 4 | 0 | 0 | 2 | 0 | 2 | 2 | 2 |
| 2013 | 1 | 0 | 1 | 0 | 0 | 2 | 0 | 0 | 0 | 1 | 0 | 0 | 0 |
| 2013 | 0 | 3 | 0 | 1 | 0 | 3 | 0 | 0 | 0 | 0 | 0 | 0 | 0 |
| 2013 | 1 | 1 | 4 | 0 | 0 | 2 | 0 | 0 | 0 | 0 | 1 | 0 | 0 |
| 2013 | 1 | 3 | 0 | 1 | 0 | 4 | 0 | 2 | 0 | 0 | 1 | 1 | 0 |
| 2013 | 0 | 2 | 1 | 0 | 0 | 1 | 0 | 0 | 0 | 0 | 1 | 0 | 0 |
| 2013 | 0 | 0 | 0 | 0 | 2 | 3 | 0 | 0 | 0 | 0 | 1 | 0 | 0 |
| 2013 | 0 | 0 | 1 | 0 | 0 | 2 | 0 | 0 | 0 | 1 | 1 | 0 | 0 |
| 2013 | 1 | 1 | 4 | 1 | 0 | 2 | 0 | 0 | 0 | 0 | 1 | 1 | 0 |
| 2013 | 0 | 4 | 4 | 1 | 2 | 0 | 0 | 2 | 0 | 1 | 1 | 0 | 0 |
| 2013 | 0 | 1 | 1 | 0 | 0 | 3 | 0 | 0 | 0 | 1 | 1 | 0 | 1 |
| 2013 | 0 | 1 | 1 | 0 | 0 | 2 | 0 | 0 | 0 | 1 | 1 | 0 | 0 |
| 2013 | 0 | 3 | 0 | 2 | 0 | 3 | 0 | 2 | 0 | 0 | 2 | 2 | 2 |
| 2013 | 0 | 1 | 2 | 1 | 1 | 3 | 0 | 0 | 0 | 0 | 1 | 0 | 0 |
| 2013 | 0 | 1 | 1 | 0 | 1 | 3 | 0 | 0 | 0 | 0 | 0 | 0 | 0 |
| 2013 | 1 | 1 | 4 | 1 | 0 | 3 | 0 | 0 | 0 | 0 | 0 | 1 | 0 |
| 2013 | 1 | 1 | 0 | 0 | 2 | 4 | 0 | 0 | 0 | 0 | 0 | 0 | 0 |
| 2013 | 0 | 1 | 1 | 1 | 0 | 2 | 0 | 2 | 0 | 1 | 1 | 0 | 0 |
| 2013 | 0 | 2 | 4 | 1 | 0 | 3 | 0 | 2 | 0 | 1 | 1 | 0 | 0 |
| 2013 | 1 | 3 | 0 | 1 | 1 | 1 | 0 | 2 | 0 | 0 | 0 | 0 | 0 |
| 2013 | 1 | 3 | 0 | 0 | 0 | 3 | 0 | 0 | 0 | 0 | 0 | 0 | 0 |
| 2013 | 0 | 0 | 4 | 1 | 1 | 3 | 0 | 0 | 0 | 2 | 1 | 0 | 0 |
| 2013 | 1 | 0 | 0 | 0 | 0 | 2 | 0 | 0 | 0 | 0 | 1 | 0 | 0 |
| 2013 | 0 | 1 | 1 | 0 | 1 | 2 | 0 | 0 | 0 | 1 | 0 | 0 | 0 |
| 2013 | 0 | 1 | 1 | 0 | 2 | 1 | 0 | 0 | 0 | 1 | 0 | 0 | 0 |
| 2013 | 1 | 2 | 1 | 1 | 0 | 2 | 0 | 0 | 0 | 1 | 0 | 0 | 0 |
| 2013 | 1 | 3 | 4 | 1 | 0 | 3 | 0 | 0 | 0 | 0 | 1 | 0 | 0 |
| 2013 | 0 | 2 | 4 | 0 | 1 | 0 | 0 | 0 | 0 | 1 | 1 | 0 | 0 |
| 2013 | 0 | 0 | 0 | 0 | 1 | 2 | 0 | 2 | 0 | 1 | 1 | 0 | 0 |
| 2013 | 1 | 2 | 0 | 0 | 1 | 3 | 0 | 0 | 0 | 1 | 0 | 0 | 0 |
| 2013 | 1 | 3 | 1 | 1 | 0 | 4 | 0 | 0 | 0 | 0 | 0 | 0 | 0 |
| 2013 | 0 | 1 | 4 | 2 | 0 | 1 | 0 | 2 | 0 | 1 | 2 | 2 | 2 |
| 2013 | 0 | 1 | 1 | 2 | 0 | 0 | 0 | 0 | 0 | 1 | 1 | 0 | 0 |
| 2013 | 1 | 2 | 0 | 0 | 0 | 2 | 0 | 0 | 0 | 1 | 1 | 0 | 0 |
| 2013 | 1 | 0 | 1 | 0 | 0 | 3 | 0 | 0 | 0 | 1 | 0 | 0 | 0 |
| 2013 | 1 | 1 | 1 | 1 | 0 | 0 | 0 | 2 | 0 | 1 | 1 | 0 | 0 |
| 2013 | 0 | 1 | 4 | 0 | 0 | 1 | 0 | 2 | 0 | 0 | 1 | 0 | 0 |
| 2013 | 0 | 1 | 0 | 1 | 1 | 3 | 0 | 0 | 0 | 1 | 1 | 0 | 0 |
| 2013 | 0 | 0 | 2 | 0 | 1 | 1 | 0 | 0 | 0 | 1 | 0 | 0 | 0 |
| 2013 | 0 | 2 | 4 | 1 | 0 | 3 | 0 | 2 | 0 | 0 | 0 | 0 | 1 |
| 2013 | 0 | 0 | 0 | 1 | 1 | 2 | 0 | 0 | 0 | 0 | 1 | 0 | 0 |
| 2013 | 0 | 1 | 1 | 0 | 0 | 0 | 0 | 0 | 0 | 0 | 0 | 0 | 0 |
| 2013 | 0 | 1 | 0 | 0 | 0 | 1 | 0 | 0 | 0 | 1 | 2 | 2 | 2 |
| 2013 | 0 | 1 | 1 | 0 | 2 | 2 | 0 | 0 | 0 | 1 | 1 | 0 | 0 |
| 2013 | 0 | 1 | 4 | 1 | 0 | 1 | 0 | 0 | 0 | 0 | 1 | 0 | 0 |
| 2013 | 0 | 1 | 0 | 0 | 0 | 1 | 0 | 0 | 0 | 1 | 1 | 0 | 0 |
| 2013 | 0 | 4 | 0 | 1 | 0 | 3 | 0 | 2 | 0 | 0 | 0 | 0 | 1 |
| 2013 | 0 | 1 | 0 | 1 | 1 | 0 | 0 | 0 | 0 | 1 | 0 | 0 | 0 |
| 2013 | 1 | 3 | 2 | 0 | 0 | 3 | 0 | 2 | 0 | 0 | 1 | 0 | 0 |
| 2013 | 1 | 3 | 1 | 1 | 1 | 1 | 0 | 2 | 0 | 1 | 1 | 0 | 0 |
| 2013 | 0 | 1 | 1 | 1 | 0 | 1 | 0 | 0 | 0 | 0 | 0 | 0 | 0 |
| 2013 | 1 | 1 | 0 | 1 | 1 | 4 | 0 | 0 | 0 | 0 | 0 | 0 | 0 |
| 2013 | 0 | 2 | 1 | 1 | 0 | 1 | 0 | 0 | 0 | 0 | 0 | 0 | 0 |
| 2013 | 1 | 2 | 1 | 1 | 0 | 2 | 0 | 2 | 0 | 1 | 0 | 0 | 0 |
| 2013 | 1 | 1 | 4 | 1 | 0 | 2 | 0 | 2 | 0 | 0 | 0 | 0 | 0 |
| 2013 | 0 | 0 | 0 | 0 | 0 | 3 | 0 | 0 | 0 | 0 | 1 | 0 | 0 |
| 2013 | 0 | 0 | 0 | 0 | 1 | 1 | 0 | 1 | 0 | 0 | 1 | 0 | 0 |
| 2013 | 1 | 2 | 1 | 1 | 0 | 0 | 0 | 0 | 0 | 1 | 1 | 0 | 0 |
| 2013 | 0 | 1 | 4 | 2 | 0 | 0 | 0 | 0 | 0 | 1 | 0 | 0 | 0 |
| 2013 | 0 | 1 | 1 | 1 | 0 | 1 | 0 | 1 | 0 | 1 | 0 | 0 | 0 |
| 2013 | 1 | 2 | 1 | 0 | 0 | 1 | 0 | 2 | 0 | 1 | 0 | 0 | 0 |
| 2013 | 0 | 2 | 0 | 1 | 1 | 4 | 0 | 0 | 0 | 0 | 1 | 1 | 0 |
| 2013 | 0 | 1 | 2 | 0 | 1 | 0 | 0 | 2 | 0 | 1 | 1 | 0 | 0 |
| 2013 | 1 | 1 | 0 | 1 | 1 | 2 | 0 | 0 | 0 | 1 | 0 | 1 | 0 |
| 2013 | 1 | 1 | 2 | 0 | 1 | 1 | 0 | 0 | 0 | 1 | 1 | 0 | 1 |
| 2013 | 1 | 3 | 4 | 2 | 1 | 0 | 0 | 0 | 0 | 0 | 0 | 0 | 0 |
| 2013 | 0 | 2 | 0 | 0 | 1 | 3 | 0 | 0 | 0 | 0 | 0 | 1 | 0 |
| 2013 | 0 | 2 | 1 | 0 | 0 | 2 | 0 | 1 | 0 | 2 | 0 | 0 | 0 |
| 2013 | 1 | 0 | 1 | 0 | 0 | 2 | 0 | 2 | 0 | 1 | 1 | 0 | 0 |
| 2013 | 1 | 4 | 4 | 1 | 1 | 0 | 0 | 2 | 0 | 0 | 1 | 0 | 0 |
| 2013 | 1 | 1 | 4 | 1 | 1 | 3 | 0 | 0 | 0 | 0 | 1 | 1 | 0 |
| 2013 | 0 | 1 | 0 | 1 | 1 | 3 | 0 | 2 | 0 | 0 | 1 | 0 | 0 |
| 2013 | 0 | 3 | 4 | 0 | 1 | 0 | 0 | 0 | 0 | 0 | 1 | 0 | 0 |
| 2013 | 0 | 0 | 1 | 1 | 1 | 1 | 0 | 0 | 0 | 1 | 0 | 1 | 0 |
| 2013 | 0 | 2 | 4 | 0 | 1 | 3 | 0 | 0 | 0 | 0 | 0 | 0 | 0 |
| 2013 | 1 | 2 | 1 | 0 | 0 | 2 | 0 | 0 | 0 | 0 | 1 | 0 | 0 |
| 2013 | 1 | 2 | 1 | 0 | 0 | 1 | 0 | 2 | 0 | 2 | 0 | 0 | 0 |
| 2013 | 0 | 1 | 0 | 2 | 1 | 2 | 0 | 2 | 0 | 1 | 2 | 2 | 2 |
| 2013 | 0 | 1 | 1 | 0 | 0 | 1 | 0 | 0 | 0 | 1 | 1 | 0 | 0 |
| 2013 | 1 | 3 | 0 | 2 | 1 | 2 | 0 | 2 | 0 | 1 | 2 | 2 | 2 |
| 2013 | 1 | 1 | 1 | 1 | 0 | 4 | 0 | 0 | 0 | 0 | 0 | 0 | 0 |
| 2013 | 0 | 0 | 0 | 1 | 2 | 0 | 1 | 0 | 0 | 2 | 0 | 0 | 0 |
| 2013 | 1 | 3 | 4 | 0 | 0 | 3 | 1 | 0 | 0 | 0 | 0 | 0 | 0 |
| 2013 | 1 | 1 | 0 | 2 | 0 | 3 | 1 | 0 | 0 | 0 | 0 | 0 | 0 |
| 2013 | 1 | 3 | 4 | 0 | 0 | 2 | 1 | 2 | 0 | 0 | 1 | 0 | 0 |
| 2013 | 1 | 3 | 3 | 1 | 0 | 4 | 1 | 0 | 0 | 0 | 1 | 0 | 0 |
| 2013 | 1 | 1 | 4 | 1 | 0 | 4 | 1 | 0 | 0 | 0 | 1 | 0 | 0 |
| 2013 | 1 | 2 | 1 | 2 | 0 | 3 | 1 | 2 | 0 | 1 | 2 | 2 | 2 |
| 2013 | 0 | 0 | 1 | 1 | 0 | 1 | 0 | 0 | 0 | 1 | 0 | 0 | 0 |
| 2013 | 1 | 1 | 1 | 1 | 0 | 2 | 0 | 2 | 0 | 0 | 0 | 0 | 1 |
| 2013 | 0 | 4 | 4 | 0 | 2 | 1 | 0 | 2 | 0 | 2 | 0 | 0 | 1 |
| 2013 | 0 | 3 | 3 | 1 | 0 | 2 | 0 | 0 | 0 | 0 | 0 | 0 | 0 |
| 2013 | 1 | 1 | 1 | 1 | 1 | 2 | 0 | 0 | 0 | 1 | 2 | 2 | 2 |
| 2013 | 0 | 2 | 4 | 2 | 0 | 2 | 0 | 2 | 0 | 0 | 2 | 2 | 2 |
| 2013 | 0 | 4 | 4 | 2 | 2 | 1 | 0 | 2 | 0 | 1 | 2 | 2 | 2 |
| 2013 | 1 | 3 | 2 | 2 | 0 | 2 | 0 | 2 | 0 | 1 | 2 | 2 | 2 |
| 2013 | 0 | 0 | 0 | 0 | 1 | 0 | 0 | 0 | 1 | 0 | 0 | 0 | 0 |
| 2013 | 0 | 1 | 0 | 0 | 0 | 2 | 0 | 0 | 0 | 0 | 0 | 0 | 0 |
| 2013 | 0 | 3 | 1 | 0 | 0 | 1 | 0 | 0 | 0 | 1 | 0 | 0 | 0 |
| 2013 | 1 | 4 | 2 | 2 | 0 | 1 | 0 | 2 | 0 | 1 | 0 | 0 | 0 |
| 2013 | 0 | 0 | 0 | 0 | 0 | 2 | 0 | 0 | 0 | 0 | 0 | 0 | 0 |
| 2013 | 0 | 0 | 4 | 2 | 0 | 2 | 0 | 0 | 0 | 0 | 0 | 0 | 1 |
| 2013 | 0 | 1 | 1 | 0 | 1 | 0 | 0 | 2 | 0 | 0 | 0 | 0 | 0 |
| 2013 | 0 | 0 | 0 | 1 | 0 | 1 | 0 | 2 | 0 | 0 | 0 | 0 | 0 |
| 2013 | 0 | 1 | 1 | 0 | 0 | 1 | 0 | 0 | 0 | 1 | 0 | 0 | 0 |
| 2013 | 1 | 3 | 1 | 0 | 1 | 0 | 0 | 2 | 0 | 0 | 0 | 0 | 0 |
| 2013 | 0 | 0 | 0 | 0 | 1 | 1 | 0 | 0 | 0 | 0 | 0 | 0 | 1 |
| 2013 | 0 | 1 | 1 | 0 | 0 | 1 | 0 | 2 | 0 | 1 | 0 | 0 | 1 |
| 2013 | 1 | 2 | 2 | 1 | 0 | 2 | 0 | 0 | 0 | 1 | 0 | 0 | 0 |
| 2013 | 1 | 3 | 0 | 1 | 0 | 1 | 0 | 1 | 0 | 0 | 0 | 0 | 0 |
| 2013 | 1 | 1 | 0 | 1 | 1 | 3 | 0 | 0 | 0 | 0 | 0 | 1 | 0 |
| 2013 | 0 | 1 | 0 | 0 | 0 | 2 | 0 | 0 | 0 | 0 | 0 | 0 | 0 |
| 2013 | 0 | 1 | 1 | 0 | 0 | 2 | 0 | 1 | 0 | 1 | 0 | 0 | 0 |
| 2013 | 0 | 1 | 1 | 0 | 0 | 1 | 0 | 0 | 0 | 1 | 0 | 0 | 1 |
| 2013 | 0 | 1 | 0 | 2 | 1 | 2 | 0 | 0 | 0 | 1 | 0 | 0 | 0 |
| 2013 | 0 | 1 | 0 | 0 | 0 | 2 | 0 | 0 | 0 | 0 | 0 | 0 | 1 |
| 2013 | 1 | 1 | 0 | 0 | 0 | 3 | 0 | 2 | 0 | 0 | 0 | 0 | 0 |
| 2013 | 1 | 1 | 1 | 1 | 0 | 1 | 0 | 0 | 0 | 1 | 0 | 0 | 0 |
| 2013 | 0 | 1 | 1 | 1 | 0 | 1 | 0 | 0 | 0 | 1 | 0 | 0 | 0 |
| 2013 | 0 | 0 | 2 | 0 | 0 | 1 | 0 | 1 | 0 | 1 | 0 | 0 | 0 |
| 2013 | 1 | 2 | 0 | 0 | 1 | 0 | 0 | 0 | 0 | 0 | 0 | 0 | 0 |
| 2013 | 0 | 1 | 1 | 0 | 0 | 2 | 0 | 0 | 0 | 1 | 0 | 0 | 0 |
| 2013 | 0 | 3 | 4 | 0 | 0 | 3 | 0 | 2 | 0 | 0 | 0 | 0 | 0 |
| 2013 | 0 | 2 | 1 | 0 | 1 | 0 | 0 | 2 | 0 | 1 | 0 | 0 | 0 |
| 2013 | 0 | 4 | 4 | 1 | 0 | 1 | 0 | 2 | 0 | 0 | 0 | 1 | 0 |
| 2013 | 1 | 2 | 2 | 1 | 1 | 1 | 0 | 2 | 0 | 0 | 0 | 0 | 0 |
| 2013 | 0 | 1 | 1 | 1 | 1 | 2 | 0 | 1 | 0 | 1 | 0 | 0 | 0 |
| 2013 | 0 | 0 | 0 | 1 | 1 | 3 | 0 | 0 | 0 | 1 | 0 | 0 | 0 |
| 2013 | 1 | 1 | 1 | 0 | 1 | 2 | 0 | 0 | 0 | 0 | 0 | 0 | 0 |
| 2013 | 1 | 1 | 0 | 0 | 1 | 1 | 0 | 2 | 0 | 1 | 0 | 0 | 0 |
| 2013 | 1 | 2 | 0 | 0 | 1 | 0 | 0 | 1 | 0 | 0 | 0 | 0 | 0 |
| 2013 | 1 | 3 | 2 | 0 | 1 | 0 | 0 | 0 | 0 | 0 | 0 | 0 | 1 |
| 2013 | 0 | 2 | 4 | 0 | 1 | 0 | 0 | 0 | 0 | 0 | 0 | 0 | 0 |
| 2013 | 0 | 4 | 4 | 2 | 0 | 0 | 0 | 2 | 0 | 0 | 2 | 2 | 2 |
| 2013 | 0 | 1 | 0 | 1 | 1 | 4 | 0 | 0 | 0 | 0 | 0 | 0 | 0 |
| 2013 | 0 | 0 | 0 | 1 | 0 | 2 | 0 | 1 | 0 | 0 | 0 | 0 | 0 |
| 2013 | 1 | 1 | 0 | 1 | 1 | 0 | 0 | 0 | 1 | 0 | 0 | 0 | 0 |
| 2013 | 0 | 0 | 1 | 0 | 0 | 1 | 0 | 0 | 0 | 1 | 0 | 0 | 0 |
| 2013 | 0 | 1 | 0 | 1 | 1 | 0 | 0 | 0 | 0 | 0 | 0 | 0 | 0 |
| 2013 | 1 | 1 | 0 | 1 | 2 | 0 | 0 | 0 | 0 | 0 | 2 | 2 | 2 |
| 2013 | 1 | 2 | 1 | 1 | 0 | 1 | 0 | 0 | 0 | 1 | 1 | 0 | 0 |
| 2013 | 1 | 2 | 1 | 2 | 1 | 0 | 0 | 0 | 1 | 0 | 0 | 0 | 0 |
| 2013 | 1 | 4 | 1 | 2 | 0 | 2 | 0 | 2 | 0 | 1 | 2 | 2 | 2 |
| 2013 | 1 | 1 | 1 | 1 | 0 | 2 | 0 | 0 | 0 | 0 | 0 | 0 | 0 |
| 2013 | 1 | 1 | 4 | 1 | 0 | 3 | 0 | 0 | 0 | 0 | 0 | 0 | 0 |
| 2013 | 1 | 1 | 0 | 0 | 1 | 0 | 0 | 0 | 1 | 1 | 0 | 0 | 0 |
| 2013 | 1 | 3 | 0 | 0 | 0 | 2 | 0 | 0 | 0 | 1 | 0 | 0 | 0 |
| 2013 | 0 | 3 | 4 | 0 | 1 | 2 | 0 | 0 | 1 | 1 | 0 | 0 | 0 |
| 2013 | 0 | 4 | 4 | 1 | 2 | 1 | 0 | 2 | 2 | 2 | 1 | 0 | 0 |
| 2013 | 1 | 1 | 1 | 0 | 0 | 1 | 0 | 0 | 0 | 1 | 0 | 0 | 0 |
| 2013 | 0 | 3 | 4 | 0 | 1 | 0 | 0 | 0 | 1 | 0 | 2 | 2 | 2 |
| 2013 | 0 | 1 | 1 | 0 | 0 | 3 | 0 | 0 | 0 | 1 | 0 | 0 | 0 |
| 2013 | 0 | 2 | 1 | 0 | 0 | 1 | 0 | 2 | 0 | 1 | 0 | 0 | 0 |
| 2013 | 0 | 3 | 1 | 0 | 1 | 1 | 0 | 2 | 0 | 1 | 0 | 0 | 0 |
| 2013 | 0 | 0 | 0 | 1 | 1 | 2 | 0 | 1 | 0 | 0 | 0 | 0 | 0 |
| 2013 | 0 | 3 | 1 | 0 | 1 | 3 | 0 | 0 | 0 | 0 | 0 | 0 | 0 |
| 2013 | 0 | 1 | 2 | 1 | 1 | 1 | 0 | 0 | 0 | 1 | 0 | 0 | 0 |
| 2013 | 1 | 1 | 0 | 0 | 1 | 3 | 0 | 0 | 0 | 0 | 0 | 0 | 0 |
| 2013 | 1 | 3 | 4 | 0 | 1 | 0 | 0 | 0 | 1 | 0 | 0 | 0 | 0 |
| 2013 | 1 | 3 | 2 | 1 | 0 | 4 | 0 | 0 | 0 | 1 | 0 | 0 | 0 |
| 2013 | 1 | 3 | 4 | 0 | 1 | 0 | 0 | 0 | 1 | 0 | 0 | 0 | 0 |
| 2013 | 0 | 2 | 1 | 1 | 1 | 1 | 0 | 0 | 0 | 2 | 0 | 0 | 0 |
| 2013 | 0 | 3 | 4 | 1 | 0 | 0 | 0 | 0 | 0 | 1 | 0 | 0 | 0 |
| 2013 | 0 | 2 | 4 | 2 | 0 | 2 | 0 | 2 | 0 | 1 | 2 | 2 | 2 |
| 2013 | 1 | 2 | 1 | 0 | 1 | 0 | 0 | 0 | 1 | 0 | 0 | 0 | 0 |
| 2013 | 0 | 3 | 1 | 1 | 1 | 0 | 0 | 0 | 0 | 1 | 0 | 0 | 0 |
| 2013 | 0 | 0 | 0 | 0 | 0 | 2 | 0 | 0 | 0 | 1 | 0 | 0 | 0 |
| 2013 | 0 | 0 | 0 | 1 | 0 | 3 | 0 | 0 | 0 | 0 | 0 | 0 | 0 |
| 2013 | 0 | 1 | 1 | 0 | 0 | 1 | 0 | 0 | 0 | 1 | 0 | 0 | 1 |
| 2013 | 0 | 2 | 1 | 0 | 1 | 4 | 0 | 0 | 0 | 0 | 0 | 0 | 0 |
| 2013 | 0 | 0 | 2 | 1 | 0 | 1 | 0 | 0 | 0 | 1 | 0 | 0 | 1 |
| 2013 | 1 | 1 | 2 | 0 | 0 | 1 | 0 | 0 | 0 | 0 | 0 | 0 | 0 |
| 2013 | 0 | 0 | 1 | 1 | 0 | 1 | 0 | 2 | 0 | 1 | 0 | 0 | 0 |
| 2013 | 0 | 3 | 0 | 0 | 1 | 0 | 0 | 0 | 0 | 1 | 0 | 0 | 0 |
| 2013 | 0 | 1 | 0 | 0 | 1 | 0 | 0 | 0 | 0 | 1 | 0 | 0 | 1 |
| 2013 | 1 | 2 | 2 | 0 | 0 | 1 | 0 | 0 | 0 | 1 | 0 | 0 | 1 |
| 2013 | 1 | 3 | 0 | 1 | 1 | 0 | 0 | 0 | 0 | 1 | 0 | 0 | 0 |
| 2013 | 0 | 1 | 0 | 0 | 0 | 0 | 0 | 0 | 0 | 0 | 0 | 0 | 0 |
| 2013 | 0 | 1 | 4 | 0 | 1 | 0 | 0 | 0 | 0 | 1 | 0 | 0 | 0 |
| 2013 | 0 | 0 | 2 | 0 | 0 | 1 | 0 | 2 | 0 | 1 | 0 | 0 | 0 |
| 2013 | 0 | 1 | 3 | 1 | 0 | 2 | 0 | 0 | 0 | 1 | 0 | 0 | 0 |
| 2013 | 1 | 1 | 0 | 0 | 1 | 0 | 0 | 0 | 1 | 1 | 0 | 0 | 0 |
| 2013 | 1 | 1 | 1 | 0 | 0 | 1 | 0 | 2 | 0 | 1 | 0 | 0 | 1 |
| 2013 | 1 | 2 | 1 | 0 | 0 | 2 | 0 | 0 | 0 | 0 | 0 | 0 | 0 |
| 2013 | 1 | 2 | 1 | 2 | 1 | 1 | 0 | 2 | 0 | 1 | 2 | 2 | 2 |
| 2013 | 0 | 2 | 1 | 0 | 1 | 0 | 0 | 2 | 0 | 1 | 0 | 0 | 1 |
| 2013 | 0 | 1 | 1 | 0 | 0 | 2 | 0 | 0 | 0 | 1 | 2 | 2 | 2 |
| 2013 | 0 | 3 | 1 | 1 | 0 | 3 | 0 | 0 | 0 | 1 | 0 | 0 | 1 |
| 2013 | 0 | 1 | 2 | 1 | 0 | 1 | 0 | 0 | 0 | 1 | 0 | 0 | 0 |
| 2013 | 0 | 1 | 1 | 0 | 0 | 3 | 0 | 0 | 0 | 0 | 0 | 0 | 0 |
| 2013 | 0 | 1 | 1 | 0 | 0 | 2 | 0 | 0 | 0 | 0 | 0 | 0 | 0 |
| 2013 | 1 | 1 | 1 | 0 | 0 | 2 | 0 | 2 | 0 | 0 | 0 | 0 | 0 |
| 2013 | 1 | 3 | 0 | 0 | 1 | 1 | 0 | 0 | 0 | 0 | 0 | 0 | 0 |
| 2013 | 1 | 2 | 1 | 1 | 2 | 1 | 0 | 2 | 0 | 1 | 0 | 0 | 0 |
| 2013 | 1 | 1 | 1 | 0 | 1 | 0 | 0 | 2 | 0 | 1 | 0 | 0 | 0 |
| 2013 | 0 | 1 | 1 | 0 | 0 | 1 | 0 | 2 | 0 | 0 | 0 | 0 | 0 |
| 2013 | 1 | 3 | 1 | 0 | 0 | 0 | 0 | 0 | 0 | 1 | 0 | 0 | 1 |
| 2013 | 0 | 0 | 0 | 1 | 1 | 2 | 0 | 2 | 0 | 0 | 0 | 0 | 1 |
| 2013 | 1 | 1 | 4 | 1 | 0 | 2 | 0 | 0 | 0 | 1 | 2 | 2 | 2 |
| 2013 | 1 | 0 | 2 | 0 | 0 | 0 | 0 | 0 | 0 | 1 | 0 | 0 | 0 |
| 2013 | 1 | 1 | 2 | 1 | 0 | 1 | 0 | 0 | 0 | 1 | 0 | 0 | 0 |
| 2013 | 0 | 2 | 1 | 0 | 1 | 1 | 0 | 0 | 0 | 1 | 2 | 2 | 2 |
| 2013 | 1 | 1 | 0 | 1 | 0 | 2 | 0 | 0 | 0 | 0 | 0 | 0 | 1 |
| 2013 | 1 | 1 | 0 | 1 | 0 | 1 | 0 | 0 | 0 | 0 | 0 | 0 | 0 |
| 2013 | 0 | 2 | 0 | 1 | 0 | 0 | 0 | 0 | 0 | 0 | 0 | 0 | 1 |
| 2013 | 1 | 3 | 0 | 1 | 1 | 3 | 0 | 0 | 0 | 1 | 0 | 0 | 0 |
| 2013 | 0 | 0 | 4 | 1 | 1 | 2 | 0 | 0 | 0 | 0 | 0 | 0 | 0 |
| 2013 | 0 | 0 | 4 | 1 | 1 | 3 | 0 | 2 | 0 | 0 | 0 | 0 | 0 |
| 2013 | 0 | 3 | 4 | 2 | 0 | 3 | 0 | 0 | 0 | 1 | 0 | 0 | 0 |
| 2013 | 0 | 1 | 2 | 0 | 0 | 2 | 0 | 0 | 0 | 0 | 2 | 2 | 2 |
| 2013 | 0 | 1 | 0 | 0 | 0 | 1 | 0 | 0 | 0 | 0 | 0 | 0 | 0 |
| 2013 | 1 | 1 | 0 | 0 | 0 | 2 | 0 | 2 | 0 | 0 | 0 | 0 | 1 |
| 2013 | 0 | 0 | 0 | 0 | 1 | 2 | 0 | 2 | 0 | 0 | 0 | 0 | 0 |
| 2013 | 1 | 1 | 1 | 0 | 0 | 2 | 0 | 0 | 0 | 1 | 0 | 0 | 0 |
| 2013 | 1 | 1 | 1 | 0 | 0 | 2 | 0 | 0 | 0 | 0 | 0 | 0 | 0 |
| 2013 | 0 | 4 | 1 | 0 | 1 | 3 | 0 | 0 | 0 | 1 | 0 | 0 | 1 |
| 2013 | 1 | 3 | 3 | 1 | 2 | 2 | 0 | 2 | 0 | 1 | 0 | 0 | 1 |
| 2013 | 1 | 1 | 0 | 0 | 0 | 2 | 0 | 2 | 0 | 0 | 0 | 0 | 0 |
| 2013 | 1 | 0 | 0 | 0 | 1 | 2 | 0 | 0 | 0 | 0 | 0 | 0 | 0 |
| 2013 | 1 | 1 | 0 | 0 | 0 | 3 | 0 | 0 | 0 | 0 | 0 | 0 | 0 |
| 2013 | 1 | 2 | 0 | 1 | 1 | 3 | 0 | 0 | 0 | 1 | 0 | 0 | 0 |
| 2013 | 1 | 4 | 0 | 2 | 0 | 2 | 0 | 0 | 0 | 0 | 0 | 0 | 0 |
| 2013 | 1 | 2 | 0 | 1 | 0 | 3 | 0 | 0 | 0 | 1 | 0 | 0 | 0 |
| 2013 | 0 | 4 | 0 | 1 | 1 | 3 | 0 | 2 | 0 | 0 | 0 | 0 | 0 |
| 2013 | 0 | 3 | 1 | 0 | 1 | 3 | 0 | 0 | 0 | 0 | 0 | 0 | 0 |
| 2013 | 0 | 2 | 1 | 0 | 0 | 1 | 0 | 0 | 0 | 1 | 0 | 0 | 1 |
| 2013 | 0 | 1 | 0 | 1 | 0 | 3 | 0 | 2 | 0 | 0 | 0 | 0 | 0 |
| 2013 | 1 | 3 | 2 | 2 | 0 | 4 | 0 | 2 | 0 | 0 | 0 | 0 | 0 |
| 2013 | 0 | 3 | 0 | 1 | 2 | 0 | 0 | 0 | 0 | 1 | 1 | 0 | 0 |
| 2013 | 0 | 2 | 1 | 1 | 0 | 3 | 0 | 2 | 0 | 0 | 0 | 0 | 0 |
| 2013 | 0 | 3 | 1 | 0 | 0 | 2 | 0 | 0 | 0 | 1 | 0 | 0 | 0 |
| 2013 | 0 | 1 | 4 | 1 | 0 | 3 | 0 | 0 | 0 | 0 | 0 | 0 | 0 |
| 2013 | 1 | 3 | 1 | 0 | 0 | 3 | 0 | 0 | 0 | 1 | 0 | 0 | 0 |
| 2013 | 1 | 4 | 1 | 1 | 0 | 3 | 0 | 0 | 0 | 1 | 0 | 0 | 0 |
| 2013 | 0 | 4 | 4 | 2 | 0 | 3 | 0 | 2 | 2 | 0 | 2 | 2 | 2 |
| 2013 | 2 | 1 | 1 | 0 | 2 | 3 | 0 | 0 | 0 | 1 | 0 | 0 | 0 |
| 2013 | 0 | 1 | 0 | 1 | 1 | 1 | 0 | 0 | 0 | 0 | 0 | 0 | 0 |
| 2013 | 1 | 0 | 0 | 0 | 1 | 2 | 0 | 0 | 0 | 0 | 0 | 0 | 0 |
| 2013 | 0 | 1 | 0 | 1 | 0 | 0 | 0 | 0 | 0 | 0 | 0 | 0 | 0 |
| 2013 | 1 | 2 | 1 | 1 | 1 | 2 | 0 | 0 | 0 | 1 | 0 | 0 | 0 |
| 2013 | 1 | 1 | 0 | 0 | 0 | 1 | 0 | 0 | 0 | 0 | 0 | 0 | 0 |
| 2013 | 0 | 0 | 1 | 1 | 0 | 1 | 0 | 0 | 0 | 0 | 0 | 0 | 0 |
| 2013 | 1 | 0 | 0 | 1 | 1 | 4 | 0 | 0 | 0 | 0 | 0 | 1 | 0 |
| 2013 | 1 | 1 | 1 | 0 | 0 | 3 | 0 | 0 | 0 | 0 | 0 | 0 | 0 |
| 2013 | 0 | 1 | 1 | 0 | 0 | 0 | 0 | 0 | 0 | 1 | 0 | 0 | 0 |
| 2013 | 1 | 0 | 1 | 1 | 0 | 1 | 0 | 0 | 0 | 1 | 0 | 0 | 0 |
| 2013 | 0 | 1 | 0 | 1 | 0 | 2 | 0 | 2 | 1 | 0 | 0 | 0 | 0 |
| 2013 | 1 | 4 | 0 | 0 | 1 | 3 | 0 | 0 | 0 | 1 | 0 | 0 | 0 |
| 2013 | 0 | 3 | 0 | 0 | 1 | 2 | 0 | 2 | 0 | 0 | 0 | 0 | 0 |
| 2013 | 1 | 0 | 4 | 1 | 0 | 3 | 0 | 0 | 0 | 0 | 0 | 0 | 0 |
| 2013 | 0 | 3 | 0 | 0 | 0 | 0 | 0 | 1 | 0 | 0 | 0 | 0 | 0 |
| 2013 | 0 | 1 | 2 | 0 | 0 | 1 | 0 | 2 | 0 | 1 | 0 | 0 | 0 |
| 2013 | 1 | 3 | 0 | 1 | 0 | 0 | 0 | 0 | 0 | 0 | 2 | 2 | 2 |
| 2013 | 1 | 1 | 1 | 0 | 0 | 1 | 0 | 0 | 0 | 1 | 0 | 0 | 0 |
| 2013 | 0 | 3 | 0 | 1 | 1 | 0 | 0 | 0 | 0 | 1 | 0 | 0 | 0 |
| 2013 | 0 | 3 | 4 | 1 | 0 | 0 | 0 | 2 | 0 | 1 | 0 | 0 | 0 |
| 2013 | 0 | 1 | 0 | 0 | 1 | 2 | 0 | 0 | 0 | 1 | 0 | 0 | 0 |
| 2013 | 1 | 1 | 0 | 2 | 0 | 0 | 0 | 2 | 0 | 1 | 2 | 2 | 2 |
| 2013 | 0 | 3 | 4 | 1 | 0 | 1 | 0 | 0 | 0 | 0 | 0 | 0 | 0 |
| 2013 | 0 | 1 | 0 | 0 | 1 | 1 | 0 | 2 | 0 | 0 | 0 | 0 | 0 |
| 2013 | 0 | 0 | 0 | 1 | 0 | 3 | 0 | 0 | 0 | 0 | 0 | 0 | 0 |
| 2013 | 1 | 2 | 1 | 1 | 0 | 3 | 0 | 0 | 0 | 1 | 0 | 0 | 0 |
| 2013 | 1 | 0 | 1 | 0 | 1 | 0 | 0 | 2 | 0 | 0 | 0 | 0 | 0 |
| 2013 | 0 | 3 | 0 | 0 | 1 | 3 | 0 | 0 | 0 | 0 | 0 | 1 | 0 |
| 2013 | 1 | 0 | 1 | 0 | 0 | 1 | 0 | 0 | 0 | 1 | 0 | 0 | 0 |
| 2013 | 1 | 2 | 4 | 2 | 0 | 2 | 0 | 2 | 0 | 0 | 2 | 2 | 2 |
| 2013 | 0 | 1 | 1 | 2 | 0 | 3 | 0 | 0 | 0 | 2 | 0 | 0 | 0 |
| 2013 | 1 | 1 | 1 | 1 | 0 | 4 | 0 | 2 | 0 | 0 | 0 | 0 | 0 |
| 2013 | 1 | 1 | 1 | 1 | 0 | 1 | 0 | 0 | 0 | 0 | 0 | 0 | 0 |
| 2013 | 1 | 0 | 0 | 0 | 0 | 2 | 0 | 2 | 0 | 0 | 0 | 0 | 0 |
| 2013 | 0 | 0 | 1 | 1 | 0 | 2 | 0 | 0 | 0 | 1 | 0 | 0 | 0 |
| 2013 | 1 | 3 | 1 | 0 | 0 | 2 | 0 | 0 | 0 | 1 | 0 | 0 | 0 |
| 2013 | 0 | 1 | 4 | 0 | 0 | 2 | 0 | 0 | 0 | 1 | 0 | 0 | 1 |
| 2013 | 1 | 0 | 1 | 2 | 0 | 1 | 0 | 2 | 0 | 0 | 2 | 2 | 2 |
| 2013 | 1 | 1 | 0 | 0 | 0 | 1 | 0 | 0 | 0 | 0 | 0 | 0 | 0 |
| 2013 | 1 | 3 | 3 | 1 | 0 | 2 | 0 | 0 | 0 | 0 | 0 | 0 | 0 |
| 2013 | 1 | 2 | 2 | 0 | 0 | 0 | 0 | 2 | 0 | 1 | 0 | 0 | 0 |
| 2013 | 1 | 1 | 0 | 1 | 0 | 2 | 0 | 0 | 0 | 0 | 0 | 0 | 0 |
| 2013 | 0 | 3 | 0 | 0 | 1 | 0 | 0 | 2 | 0 | 0 | 0 | 1 | 0 |
| 2013 | 1 | 3 | 0 | 0 | 0 | 2 | 0 | 0 | 0 | 0 | 0 | 0 | 0 |
| 2013 | 0 | 0 | 1 | 0 | 1 | 1 | 0 | 0 | 0 | 0 | 0 | 0 | 0 |
| 2013 | 1 | 0 | 1 | 2 | 0 | 0 | 0 | 0 | 0 | 0 | 0 | 0 | 0 |
| 2013 | 1 | 1 | 1 | 1 | 0 | 2 | 0 | 0 | 0 | 1 | 0 | 0 | 0 |
| 2013 | 0 | 1 | 1 | 0 | 0 | 3 | 0 | 0 | 0 | 1 | 0 | 1 | 0 |
| 2013 | 0 | 3 | 1 | 0 | 0 | 0 | 0 | 0 | 0 | 0 | 0 | 0 | 0 |
| 2013 | 0 | 1 | 4 | 0 | 0 | 2 | 0 | 0 | 0 | 0 | 0 | 0 | 0 |
| 2013 | 1 | 3 | 0 | 2 | 1 | 2 | 0 | 2 | 0 | 0 | 2 | 2 | 2 |
| 2013 | 0 | 2 | 0 | 0 | 1 | 0 | 0 | 2 | 1 | 0 | 1 | 0 | 0 |
| 2013 | 0 | 1 | 2 | 0 | 0 | 0 | 0 | 2 | 0 | 1 | 0 | 0 | 0 |
| 2013 | 1 | 1 | 1 | 1 | 0 | 2 | 0 | 0 | 0 | 0 | 0 | 0 | 0 |
| 2013 | 0 | 1 | 1 | 0 | 0 | 0 | 0 | 2 | 0 | 1 | 0 | 0 | 1 |
| 2013 | 1 | 3 | 1 | 0 | 0 | 2 | 0 | 0 | 0 | 0 | 0 | 0 | 0 |
| 2013 | 1 | 2 | 1 | 1 | 0 | 2 | 0 | 1 | 0 | 1 | 0 | 0 | 1 |
| 2013 | 0 | 3 | 1 | 0 | 0 | 0 | 0 | 0 | 0 | 0 | 0 | 0 | 0 |
| 2013 | 1 | 3 | 4 | 1 | 0 | 2 | 0 | 0 | 0 | 1 | 0 | 0 | 0 |
| 2013 | 1 | 3 | 1 | 1 | 0 | 1 | 0 | 2 | 0 | 1 | 0 | 0 | 0 |
| 2013 | 1 | 1 | 4 | 0 | 0 | 0 | 0 | 0 | 0 | 0 | 0 | 1 | 0 |
| 2013 | 1 | 3 | 0 | 0 | 0 | 1 | 0 | 0 | 0 | 1 | 0 | 0 | 0 |
| 2013 | 0 | 3 | 1 | 1 | 0 | 2 | 0 | 2 | 0 | 1 | 0 | 0 | 0 |
| 2013 | 1 | 1 | 0 | 0 | 0 | 1 | 0 | 2 | 0 | 1 | 0 | 0 | 0 |
| 2013 | 1 | 2 | 4 | 1 | 2 | 1 | 0 | 0 | 0 | 1 | 0 | 0 | 0 |
| 2013 | 1 | 2 | 0 | 0 | 1 | 2 | 1 | 0 | 0 | 0 | 0 | 0 | 0 |
| 2013 | 0 | 1 | 0 | 2 | 0 | 3 | 1 | 2 | 0 | 0 | 2 | 2 | 2 |
| 2013 | 0 | 0 | 0 | 2 | 0 | 1 | 1 | 2 | 0 | 0 | 2 | 2 | 2 |
| 2013 | 1 | 1 | 0 | 1 | 0 | 3 | 1 | 2 | 0 | 0 | 0 | 1 | 0 |
| 2013 | 1 | 0 | 1 | 1 | 0 | 3 | 1 | 0 | 0 | 1 | 0 | 0 | 0 |
| 2013 | 1 | 3 | 0 | 1 | 0 | 3 | 1 | 0 | 0 | 0 | 0 | 0 | 0 |
| 2013 | 1 | 1 | 0 | 1 | 1 | 3 | 1 | 0 | 0 | 0 | 1 | 0 | 0 |
| 2013 | 0 | 0 | 1 | 0 | 1 | 2 | 1 | 1 | 0 | 1 | 0 | 0 | 0 |
| 2013 | 1 | 1 | 0 | 0 | 0 | 3 | 1 | 0 | 0 | 0 | 0 | 1 | 0 |
| 2013 | 1 | 3 | 2 | 0 | 0 | 1 | 1 | 0 | 0 | 1 | 0 | 0 | 0 |
| 2013 | 1 | 3 | 0 | 1 | 0 | 3 | 1 | 0 | 0 | 0 | 0 | 0 | 0 |
| 2013 | 0 | 0 | 0 | 1 | 0 | 3 | 1 | 1 | 0 | 0 | 0 | 0 | 1 |
| 2013 | 1 | 2 | 0 | 2 | 1 | 4 | 1 | 2 | 0 | 0 | 0 | 0 | 0 |
| 2013 | 1 | 2 | 1 | 1 | 1 | 0 | 1 | 1 | 0 | 1 | 0 | 0 | 0 |
| 2013 | 1 | 0 | 2 | 0 | 2 | 2 | 1 | 0 | 0 | 1 | 0 | 0 | 0 |
| 2013 | 0 | 4 | 1 | 2 | 0 | 1 | 1 | 2 | 0 | 1 | 2 | 2 | 2 |
| 2013 | 0 | 3 | 0 | 0 | 1 | 2 | 1 | 1 | 0 | 0 | 0 | 0 | 0 |
| 2013 | 0 | 1 | 0 | 0 | 0 | 3 | 1 | 2 | 0 | 0 | 0 | 0 | 0 |
| 2013 | 1 | 0 | 1 | 1 | 0 | 2 | 1 | 0 | 0 | 0 | 0 | 0 | 0 |
| 2013 | 0 | 1 | 2 | 0 | 0 | 1 | 1 | 0 | 0 | 0 | 0 | 0 | 0 |
| 2013 | 0 | 1 | 0 | 1 | 0 | 3 | 1 | 0 | 0 | 1 | 0 | 0 | 0 |
| 2013 | 1 | 1 | 1 | 1 | 1 | 3 | 1 | 2 | 0 | 0 | 0 | 1 | 0 |
| 2013 | 1 | 1 | 0 | 2 | 1 | 0 | 0 | 2 | 1 | 2 | 0 | 0 | 0 |
| 2013 | 0 | 0 | 0 | 0 | 0 | 1 | 0 | 0 | 0 | 0 | 0 | 0 | 0 |
| 2013 | 1 | 0 | 1 | 2 | 0 | 2 | 0 | 0 | 0 | 2 | 2 | 2 | 2 |
| 2013 | 1 | 4 | 1 | 1 | 2 | 1 | 0 | 0 | 0 | 0 | 0 | 0 | 0 |
| 2013 | 0 | 1 | 1 | 0 | 0 | 3 | 0 | 2 | 0 | 2 | 2 | 2 | 2 |
| 2013 | 1 | 1 | 2 | 2 | 2 | 3 | 0 | 2 | 0 | 1 | 2 | 2 | 2 |
| 2013 | 0 | 1 | 2 | 2 | 0 | 2 | 1 | 2 | 0 | 0 | 0 | 1 | 0 |
| 2013 | 1 | 3 | 1 | 1 | 0 | 0 | 0 | 1 | 0 | 2 | 1 | 0 | 0 |
| 2013 | 0 | 2 | 1 | 0 | 0 | 0 | 0 | 0 | 0 | 0 | 0 | 0 | 0 |
| 2013 | 0 | 3 | 2 | 1 | 0 | 2 | 1 | 0 | 0 | 1 | 0 | 0 | 0 |
| 2013 | 0 | 2 | 1 | 1 | 2 | 1 | 0 | 0 | 0 | 1 | 0 | 0 | 0 |
| 2013 | 1 | 1 | 3 | 1 | 0 | 1 | 0 | 0 | 0 | 1 | 0 | 0 | 0 |
| 2013 | 0 | 2 | 0 | 1 | 0 | 3 | 0 | 2 | 0 | 0 | 0 | 0 | 0 |
| 2013 | 0 | 0 | 4 | 2 | 0 | 2 | 0 | 2 | 0 | 1 | 2 | 2 | 2 |
| 2013 | 0 | 2 | 0 | 1 | 0 | 4 | 0 | 0 | 0 | 0 | 0 | 0 | 0 |
| 2013 | 0 | 2 | 1 | 1 | 1 | 3 | 0 | 0 | 0 | 1 | 0 | 0 | 0 |
| 2013 | 1 | 2 | 1 | 1 | 0 | 3 | 0 | 2 | 0 | 0 | 0 | 0 | 0 |
| 2013 | 1 | 0 | 1 | 1 | 1 | 1 | 0 | 0 | 0 | 1 | 0 | 0 | 0 |
| 2013 | 0 | 3 | 3 | 1 | 1 | 2 | 0 | 0 | 0 | 2 | 0 | 0 | 0 |
| 2013 | 0 | 3 | 2 | 2 | 0 | 0 | 0 | 2 | 0 | 1 | 2 | 2 | 2 |
| 2013 | 0 | 3 | 0 | 2 | 0 | 2 | 0 | 2 | 0 | 0 | 2 | 2 | 2 |
| 2013 | 1 | 3 | 4 | 1 | 0 | 3 | 0 | 1 | 0 | 0 | 0 | 0 | 0 |
| 2013 | 1 | 3 | 2 | 2 | 0 | 4 | 0 | 2 | 0 | 0 | 2 | 2 | 2 |
| 2013 | 0 | 2 | 2 | 2 | 0 | 4 | 0 | 2 | 0 | 0 | 2 | 2 | 2 |
| 2013 | 1 | 0 | 0 | 1 | 0 | 1 | 0 | 0 | 0 | 0 | 0 | 0 | 0 |
| 2013 | 0 | 1 | 1 | 2 | 1 | 1 | 0 | 1 | 0 | 2 | 2 | 2 | 2 |
| 2013 | 0 | 2 | 0 | 1 | 1 | 3 | 0 | 0 | 0 | 0 | 0 | 0 | 0 |
| 2013 | 1 | 1 | 0 | 2 | 0 | 2 | 1 | 2 | 0 | 1 | 2 | 2 | 2 |
| 2013 | 0 | 4 | 0 | 2 | 0 | 1 | 0 | 0 | 0 | 1 | 2 | 2 | 2 |
| 2013 | 2 | 1 | 4 | 1 | 0 | 1 | 0 | 2 | 0 | 0 | 0 | 0 | 0 |
| 2013 | 1 | 0 | 4 | 2 | 1 | 1 | 1 | 2 | 0 | 2 | 0 | 0 | 0 |
| 2013 | 1 | 2 | 1 | 1 | 0 | 2 | 1 | 0 | 0 | 1 | 0 | 0 | 0 |
| 2013 | 1 | 0 | 0 | 0 | 0 | 2 | 0 | 0 | 0 | 0 | 2 | 2 | 2 |
| 2013 | 1 | 1 | 1 | 0 | 1 | 0 | 0 | 0 | 0 | 1 | 0 | 0 | 0 |
| 2013 | 1 | 2 | 1 | 1 | 1 | 3 | 0 | 0 | 0 | 0 | 0 | 0 | 0 |
| 2013 | 1 | 0 | 1 | 1 | 0 | 2 | 1 | 0 | 0 | 1 | 0 | 0 | 0 |
| 2013 | 1 | 2 | 1 | 0 | 1 | 1 | 0 | 0 | 0 | 1 | 0 | 1 | 0 |
| 2013 | 0 | 4 | 1 | 0 | 0 | 2 | 0 | 0 | 0 | 0 | 2 | 2 | 2 |
| 2013 | 1 | 0 | 0 | 1 | 0 | 3 | 1 | 0 | 0 | 0 | 0 | 0 | 0 |
| 2014 | 0 | 0 | 1 | 2 | 0 | 3 | 1 | 2 | 0 | 0 | 2 | 2 | 2 |
| 2014 | 1 | 2 | 0 | 1 | 1 | 0 | 0 | 0 | 0 | 1 | 0 | 0 | 0 |
| 2014 | 0 | 0 | 2 | 0 | 0 | 2 | 0 | 2 | 0 | 2 | 0 | 0 | 0 |
| 2014 | 0 | 0 | 0 | 0 | 1 | 3 | 0 | 2 | 0 | 0 | 0 | 0 | 1 |
| 2014 | 0 | 4 | 4 | 2 | 0 | 0 | 1 | 2 | 2 | 2 | 2 | 2 | 2 |
| 2014 | 1 | 0 | 0 | 1 | 0 | 2 | 0 | 0 | 0 | 0 | 0 | 0 | 0 |
| 2014 | 0 | 1 | 2 | 1 | 0 | 1 | 0 | 1 | 0 | 1 | 0 | 1 | 0 |
| 2014 | 0 | 1 | 0 | 0 | 0 | 2 | 0 | 1 | 0 | 0 | 0 | 1 | 0 |
| 2014 | 0 | 0 | 0 | 1 | 0 | 1 | 0 | 0 | 0 | 0 | 2 | 2 | 2 |
| 2014 | 0 | 2 | 4 | 1 | 1 | 3 | 0 | 2 | 0 | 0 | 0 | 0 | 0 |
| 2014 | 1 | 1 | 0 | 1 | 1 | 3 | 0 | 0 | 0 | 0 | 0 | 0 | 0 |
| 2014 | 0 | 3 | 1 | 2 | 1 | 3 | 0 | 2 | 0 | 1 | 2 | 2 | 2 |
| 2014 | 1 | 3 | 4 | 1 | 0 | 2 | 0 | 2 | 0 | 0 | 0 | 0 | 1 |
| 2014 | 1 | 0 | 1 | 1 | 0 | 2 | 0 | 0 | 0 | 1 | 0 | 0 | 0 |
| 2014 | 0 | 1 | 0 | 0 | 0 | 2 | 0 | 0 | 0 | 0 | 0 | 0 | 0 |
| 2014 | 0 | 0 | 1 | 1 | 0 | 2 | 0 | 0 | 0 | 1 | 2 | 2 | 2 |
| 2014 | 0 | 0 | 0 | 0 | 0 | 2 | 0 | 0 | 0 | 0 | 0 | 0 | 0 |
| 2014 | 1 | 3 | 1 | 1 | 0 | 2 | 0 | 0 | 0 | 1 | 0 | 0 | 0 |
| 2014 | 0 | 1 | 0 | 0 | 1 | 3 | 0 | 1 | 0 | 0 | 0 | 0 | 0 |
| 2014 | 0 | 1 | 2 | 0 | 0 | 2 | 0 | 0 | 0 | 1 | 0 | 0 | 0 |
| 2014 | 1 | 1 | 4 | 0 | 1 | 0 | 0 | 0 | 0 | 2 | 2 | 2 | 2 |
| 2014 | 1 | 0 | 0 | 1 | 0 | 4 | 0 | 0 | 0 | 0 | 0 | 0 | 0 |
| 2014 | 1 | 1 | 1 | 1 | 1 | 1 | 0 | 2 | 0 | 1 | 0 | 0 | 0 |
| 2014 | 1 | 0 | 1 | 1 | 0 | 2 | 0 | 0 | 0 | 1 | 0 | 0 | 0 |
| 2014 | 0 | 1 | 1 | 1 | 0 | 4 | 0 | 0 | 0 | 0 | 0 | 0 | 0 |
| 2014 | 1 | 1 | 4 | 1 | 1 | 0 | 0 | 0 | 0 | 0 | 0 | 0 | 0 |
| 2014 | 1 | 0 | 1 | 1 | 0 | 4 | 1 | 0 | 0 | 0 | 0 | 0 | 0 |
| 2014 | 0 | 2 | 1 | 2 | 0 | 2 | 1 | 0 | 0 | 0 | 2 | 2 | 2 |
| 2014 | 1 | 0 | 1 | 0 | 0 | 2 | 1 | 2 | 0 | 0 | 0 | 0 | 0 |
| 2014 | 1 | 0 | 0 | 1 | 0 | 2 | 1 | 0 | 0 | 0 | 0 | 0 | 0 |
| 2014 | 0 | 0 | 1 | 1 | 1 | 2 | 1 | 0 | 0 | 0 | 0 | 0 | 0 |
| 2014 | 1 | 0 | 1 | 1 | 0 | 3 | 1 | 0 | 0 | 0 | 0 | 0 | 0 |
| 2014 | 1 | 1 | 2 | 1 | 0 | 2 | 1 | 1 | 0 | 1 | 1 | 0 | 0 |
| 2014 | 1 | 1 | 0 | 0 | 1 | 1 | 1 | 0 | 0 | 0 | 0 | 0 | 0 |
| 2014 | 1 | 1 | 1 | 0 | 1 | 3 | 1 | 0 | 0 | 1 | 0 | 0 | 0 |
| 2014 | 1 | 3 | 4 | 0 | 0 | 1 | 1 | 0 | 0 | 1 | 0 | 0 | 0 |
| 2014 | 0 | 1 | 0 | 1 | 0 | 4 | 1 | 0 | 0 | 0 | 0 | 0 | 0 |
| 2014 | 1 | 4 | 4 | 2 | 2 | 2 | 1 | 2 | 0 | 2 | 2 | 2 | 2 |
| 2014 | 0 | 1 | 1 | 1 | 0 | 2 | 1 | 2 | 0 | 1 | 0 | 0 | 0 |
| 2014 | 1 | 3 | 1 | 1 | 0 | 3 | 1 | 0 | 0 | 0 | 0 | 0 | 0 |
| 2014 | 1 | 0 | 1 | 1 | 0 | 2 | 1 | 0 | 0 | 0 | 0 | 1 | 0 |
| 2014 | 1 | 0 | 0 | 1 | 0 | 2 | 1 | 0 | 0 | 0 | 0 | 0 | 0 |
| 2014 | 0 | 1 | 1 | 0 | 0 | 3 | 0 | 0 | 0 | 0 | 0 | 0 | 0 |
| 2014 | 0 | 2 | 0 | 1 | 0 | 3 | 0 | 2 | 0 | 1 | 0 | 0 | 0 |
| 2014 | 1 | 3 | 0 | 0 | 1 | 3 | 0 | 0 | 0 | 0 | 0 | 0 | 1 |
| 2014 | 1 | 3 | 3 | 1 | 0 | 2 | 0 | 0 | 0 | 1 | 0 | 0 | 0 |
| 2014 | 0 | 3 | 1 | 1 | 0 | 2 | 0 | 2 | 0 | 0 | 0 | 0 | 0 |
| 2014 | 0 | 3 | 2 | 1 | 0 | 2 | 0 | 0 | 0 | 1 | 0 | 0 | 0 |
| 2014 | 0 | 3 | 3 | 1 | 0 | 3 | 0 | 2 | 0 | 1 | 0 | 0 | 0 |
| 2014 | 1 | 1 | 1 | 1 | 0 | 3 | 0 | 0 | 0 | 0 | 0 | 0 | 0 |
| 2014 | 1 | 3 | 1 | 0 | 1 | 1 | 0 | 0 | 0 | 0 | 0 | 0 | 0 |
| 2014 | 1 | 0 | 1 | 1 | 0 | 3 | 0 | 0 | 0 | 0 | 0 | 0 | 0 |
| 2014 | 0 | 3 | 1 | 1 | 1 | 2 | 0 | 0 | 0 | 1 | 0 | 0 | 0 |
| 2014 | 0 | 1 | 4 | 1 | 0 | 1 | 0 | 0 | 0 | 1 | 0 | 0 | 0 |
| 2014 | 1 | 3 | 0 | 1 | 0 | 4 | 0 | 0 | 0 | 0 | 0 | 0 | 0 |
| 2014 | 0 | 0 | 0 | 1 | 0 | 4 | 0 | 0 | 0 | 0 | 0 | 0 | 0 |
| 2014 | 1 | 3 | 2 | 1 | 1 | 2 | 0 | 0 | 0 | 1 | 0 | 0 | 0 |
| 2014 | 1 | 1 | 0 | 1 | 0 | 4 | 0 | 0 | 0 | 0 | 0 | 0 | 0 |
| 2014 | 1 | 2 | 1 | 1 | 0 | 0 | 0 | 0 | 0 | 2 | 0 | 0 | 0 |
| 2014 | 1 | 3 | 2 | 0 | 2 | 3 | 0 | 0 | 0 | 1 | 0 | 0 | 1 |
| 2014 | 1 | 0 | 1 | 1 | 0 | 2 | 0 | 2 | 0 | 0 | 0 | 0 | 0 |
| 2014 | 0 | 3 | 3 | 1 | 1 | 0 | 0 | 0 | 1 | 1 | 0 | 0 | 0 |
| 2014 | 1 | 0 | 4 | 1 | 2 | 4 | 0 | 2 | 0 | 0 | 0 | 0 | 0 |
| 2014 | 1 | 3 | 0 | 0 | 0 | 3 | 0 | 2 | 0 | 0 | 0 | 1 | 0 |
| 2014 | 1 | 3 | 1 | 1 | 1 | 0 | 0 | 2 | 0 | 1 | 0 | 0 | 0 |
| 2014 | 0 | 1 | 1 | 1 | 0 | 2 | 0 | 0 | 0 | 1 | 0 | 0 | 0 |
| 2014 | 0 | 0 | 0 | 0 | 0 | 3 | 0 | 0 | 0 | 1 | 0 | 0 | 0 |
| 2014 | 0 | 1 | 1 | 1 | 0 | 2 | 0 | 0 | 0 | 0 | 0 | 0 | 0 |
| 2014 | 1 | 1 | 0 | 0 | 0 | 3 | 0 | 0 | 0 | 1 | 0 | 0 | 0 |
| 2014 | 1 | 3 | 0 | 0 | 1 | 0 | 0 | 2 | 0 | 1 | 0 | 0 | 0 |
| 2014 | 0 | 0 | 1 | 1 | 0 | 2 | 0 | 2 | 0 | 0 | 0 | 0 | 0 |
| 2014 | 0 | 0 | 0 | 1 | 1 | 1 | 0 | 0 | 0 | 0 | 0 | 0 | 0 |
| 2014 | 1 | 3 | 1 | 2 | 1 | 0 | 0 | 2 | 0 | 1 | 2 | 2 | 2 |
| 2014 | 0 | 0 | 1 | 0 | 1 | 2 | 0 | 2 | 0 | 0 | 0 | 0 | 0 |
| 2014 | 1 | 1 | 0 | 1 | 0 | 2 | 0 | 2 | 0 | 0 | 0 | 0 | 0 |
| 2014 | 0 | 1 | 2 | 0 | 0 | 0 | 0 | 0 | 0 | 1 | 0 | 0 | 0 |
| 2014 | 0 | 1 | 4 | 2 | 0 | 2 | 0 | 2 | 0 | 0 | 2 | 2 | 2 |
| 2014 | 0 | 3 | 3 | 1 | 0 | 1 | 0 | 0 | 0 | 1 | 0 | 0 | 0 |
| 2014 | 0 | 1 | 4 | 0 | 1 | 0 | 0 | 0 | 0 | 0 | 0 | 0 | 0 |
| 2014 | 1 | 2 | 3 | 1 | 0 | 1 | 0 | 0 | 0 | 1 | 0 | 0 | 0 |
| 2014 | 1 | 1 | 0 | 1 | 0 | 4 | 0 | 0 | 0 | 0 | 0 | 0 | 0 |
| 2014 | 0 | 1 | 1 | 1 | 0 | 3 | 0 | 1 | 0 | 0 | 0 | 0 | 0 |
| 2014 | 0 | 0 | 1 | 1 | 0 | 2 | 0 | 1 | 0 | 1 | 0 | 0 | 0 |
| 2014 | 0 | 4 | 2 | 2 | 0 | 3 | 0 | 0 | 0 | 1 | 0 | 0 | 0 |
| 2014 | 0 | 0 | 1 | 1 | 0 | 3 | 0 | 0 | 0 | 0 | 0 | 0 | 0 |
| 2014 | 0 | 3 | 4 | 2 | 0 | 1 | 0 | 0 | 0 | 1 | 0 | 0 | 1 |
| 2014 | 0 | 3 | 1 | 0 | 1 | 2 | 0 | 0 | 0 | 0 | 0 | 0 | 1 |
| 2014 | 1 | 0 | 0 | 1 | 0 | 4 | 0 | 0 | 0 | 2 | 2 | 2 | 2 |
| 2014 | 0 | 3 | 1 | 1 | 0 | 2 | 0 | 0 | 0 | 1 | 0 | 0 | 0 |
| 2014 | 0 | 0 | 1 | 1 | 0 | 2 | 0 | 0 | 0 | 1 | 0 | 0 | 0 |
| 2014 | 1 | 3 | 3 | 1 | 0 | 2 | 0 | 0 | 0 | 1 | 0 | 0 | 0 |
| 2014 | 0 | 0 | 2 | 2 | 0 | 1 | 0 | 2 | 0 | 1 | 0 | 0 | 1 |
| 2014 | 1 | 0 | 3 | 1 | 0 | 1 | 0 | 2 | 0 | 1 | 0 | 0 | 0 |
| 2014 | 0 | 1 | 4 | 1 | 0 | 0 | 0 | 0 | 0 | 1 | 0 | 0 | 0 |
| 2014 | 1 | 3 | 1 | 1 | 0 | 2 | 0 | 1 | 0 | 1 | 0 | 0 | 0 |
| 2014 | 0 | 1 | 0 | 1 | 1 | 1 | 0 | 0 | 0 | 0 | 0 | 0 | 0 |
| 2014 | 1 | 3 | 3 | 1 | 1 | 0 | 0 | 0 | 1 | 1 | 0 | 0 | 0 |
| 2014 | 0 | 0 | 4 | 2 | 0 | 2 | 0 | 0 | 0 | 0 | 0 | 0 | 0 |
| 2014 | 1 | 2 | 1 | 1 | 0 | 4 | 0 | 0 | 0 | 0 | 0 | 0 | 0 |
| 2014 | 0 | 1 | 1 | 0 | 0 | 2 | 0 | 1 | 0 | 1 | 0 | 1 | 0 |
| 2014 | 0 | 3 | 0 | 0 | 1 | 0 | 0 | 0 | 0 | 0 | 0 | 0 | 0 |
| 2014 | 1 | 1 | 1 | 1 | 0 | 2 | 0 | 0 | 0 | 1 | 0 | 0 | 0 |
| 2014 | 0 | 1 | 0 | 0 | 1 | 0 | 0 | 0 | 0 | 0 | 2 | 2 | 2 |
| 2014 | 0 | 1 | 1 | 1 | 0 | 3 | 0 | 2 | 0 | 0 | 0 | 0 | 0 |
| 2014 | 1 | 3 | 1 | 0 | 1 | 2 | 0 | 0 | 0 | 1 | 0 | 0 | 0 |
| 2014 | 0 | 2 | 3 | 0 | 1 | 0 | 0 | 1 | 1 | 1 | 0 | 1 | 0 |
| 2014 | 0 | 3 | 4 | 2 | 1 | 1 | 0 | 0 | 0 | 1 | 0 | 0 | 0 |
| 2014 | 1 | 1 | 1 | 0 | 0 | 3 | 0 | 0 | 0 | 1 | 0 | 0 | 0 |
| 2014 | 0 | 3 | 1 | 0 | 0 | 0 | 0 | 0 | 0 | 0 | 0 | 0 | 0 |
| 2014 | 1 | 3 | 1 | 1 | 1 | 0 | 0 | 0 | 0 | 1 | 2 | 2 | 2 |
| 2014 | 0 | 1 | 2 | 0 | 0 | 2 | 0 | 0 | 0 | 0 | 0 | 0 | 0 |
| 2014 | 0 | 1 | 1 | 1 | 0 | 2 | 0 | 0 | 0 | 0 | 0 | 0 | 1 |
| 2014 | 0 | 0 | 1 | 1 | 1 | 3 | 0 | 0 | 0 | 1 | 0 | 0 | 0 |
| 2014 | 1 | 1 | 1 | 0 | 0 | 2 | 0 | 2 | 0 | 1 | 0 | 0 | 0 |
| 2014 | 1 | 1 | 1 | 1 | 0 | 2 | 0 | 0 | 0 | 2 | 0 | 0 | 0 |
| 2014 | 0 | 3 | 4 | 0 | 1 | 0 | 0 | 2 | 1 | 0 | 0 | 0 | 0 |
| 2014 | 0 | 3 | 4 | 2 | 1 | 1 | 0 | 2 | 0 | 1 | 2 | 2 | 2 |
| 2014 | 2 | 1 | 1 | 0 | 0 | 3 | 0 | 0 | 0 | 1 | 0 | 0 | 0 |
| 2014 | 0 | 4 | 0 | 1 | 2 | 2 | 0 | 0 | 0 | 0 | 0 | 0 | 0 |
| 2014 | 1 | 1 | 4 | 1 | 0 | 4 | 0 | 0 | 0 | 0 | 0 | 0 | 0 |
| 2014 | 1 | 1 | 0 | 0 | 2 | 2 | 0 | 0 | 0 | 0 | 0 | 0 | 1 |
| 2014 | 1 | 1 | 1 | 1 | 0 | 2 | 0 | 1 | 0 | 1 | 0 | 0 | 0 |
| 2014 | 0 | 3 | 0 | 1 | 0 | 0 | 0 | 2 | 0 | 1 | 0 | 0 | 0 |
| 2014 | 1 | 0 | 4 | 1 | 0 | 3 | 0 | 0 | 0 | 2 | 0 | 0 | 0 |
| 2014 | 0 | 1 | 1 | 1 | 1 | 0 | 0 | 0 | 0 | 1 | 0 | 0 | 0 |
| 2014 | 0 | 3 | 0 | 1 | 1 | 0 | 0 | 2 | 1 | 0 | 0 | 0 | 0 |
| 2014 | 0 | 0 | 4 | 2 | 1 | 0 | 0 | 2 | 0 | 0 | 2 | 2 | 2 |
| 2014 | 1 | 1 | 4 | 1 | 0 | 3 | 0 | 0 | 0 | 0 | 0 | 0 | 1 |
| 2014 | 2 | 1 | 2 | 1 | 0 | 3 | 0 | 0 | 0 | 0 | 0 | 0 | 0 |
| 2014 | 0 | 3 | 1 | 0 | 0 | 0 | 0 | 2 | 0 | 1 | 0 | 0 | 0 |
| 2014 | 0 | 2 | 0 | 1 | 1 | 0 | 0 | 0 | 1 | 1 | 0 | 0 | 0 |
| 2014 | 1 | 2 | 1 | 1 | 0 | 2 | 0 | 2 | 0 | 1 | 2 | 2 | 2 |
| 2014 | 1 | 3 | 4 | 2 | 1 | 0 | 0 | 2 | 1 | 1 | 0 | 0 | 0 |
| 2014 | 0 | 1 | 1 | 0 | 0 | 0 | 0 | 0 | 0 | 0 | 0 | 0 | 0 |
| 2014 | 0 | 3 | 3 | 1 | 0 | 1 | 0 | 2 | 0 | 1 | 2 | 2 | 2 |
| 2014 | 1 | 3 | 2 | 1 | 0 | 3 | 0 | 0 | 0 | 0 | 0 | 0 | 0 |
| 2014 | 1 | 1 | 1 | 1 | 0 | 1 | 0 | 0 | 0 | 1 | 0 | 0 | 0 |
| 2014 | 0 | 3 | 0 | 0 | 1 | 0 | 0 | 2 | 0 | 1 | 0 | 0 | 0 |
| 2014 | 1 | 1 | 1 | 0 | 0 | 2 | 0 | 0 | 0 | 1 | 0 | 0 | 0 |
| 2014 | 0 | 2 | 1 | 0 | 0 | 0 | 0 | 0 | 0 | 0 | 0 | 0 | 0 |
| 2014 | 0 | 1 | 0 | 0 | 1 | 2 | 0 | 0 | 0 | 1 | 2 | 2 | 2 |
| 2014 | 0 | 0 | 0 | 0 | 0 | 3 | 0 | 1 | 0 | 1 | 0 | 0 | 0 |
| 2014 | 0 | 1 | 1 | 1 | 1 | 0 | 0 | 0 | 0 | 1 | 0 | 0 | 0 |
| 2014 | 0 | 1 | 0 | 1 | 1 | 4 | 0 | 0 | 0 | 0 | 0 | 0 | 0 |
| 2014 | 0 | 1 | 1 | 0 | 2 | 2 | 0 | 2 | 0 | 1 | 0 | 0 | 0 |
| 2014 | 0 | 0 | 1 | 1 | 0 | 2 | 0 | 1 | 0 | 1 | 2 | 2 | 2 |
| 2014 | 0 | 3 | 1 | 1 | 0 | 0 | 0 | 1 | 0 | 0 | 2 | 2 | 2 |
| 2014 | 1 | 1 | 0 | 0 | 0 | 1 | 0 | 0 | 0 | 0 | 0 | 0 | 0 |
| 2014 | 1 | 1 | 1 | 1 | 0 | 3 | 0 | 0 | 0 | 2 | 0 | 0 | 0 |
| 2014 | 1 | 1 | 0 | 2 | 0 | 2 | 0 | 0 | 0 | 0 | 0 | 0 | 0 |
| 2014 | 1 | 3 | 2 | 1 | 2 | 0 | 0 | 0 | 0 | 1 | 0 | 0 | 0 |
| 2014 | 1 | 0 | 0 | 1 | 1 | 1 | 0 | 0 | 0 | 0 | 0 | 0 | 0 |
| 2014 | 0 | 3 | 1 | 1 | 0 | 2 | 0 | 0 | 0 | 2 | 0 | 0 | 0 |
| 2014 | 0 | 0 | 0 | 1 | 0 | 4 | 0 | 0 | 0 | 0 | 0 | 0 | 0 |
| 2014 | 0 | 3 | 1 | 1 | 0 | 2 | 0 | 0 | 0 | 1 | 0 | 0 | 0 |
| 2014 | 1 | 1 | 4 | 2 | 0 | 1 | 0 | 2 | 0 | 1 | 0 | 0 | 0 |
| 2014 | 0 | 1 | 1 | 2 | 1 | 2 | 0 | 0 | 0 | 1 | 0 | 0 | 0 |
| 2014 | 1 | 1 | 1 | 1 | 1 | 0 | 0 | 0 | 0 | 1 | 2 | 2 | 2 |
| 2014 | 0 | 1 | 1 | 1 | 0 | 1 | 0 | 0 | 0 | 2 | 0 | 0 | 0 |
| 2014 | 0 | 1 | 4 | 2 | 0 | 1 | 0 | 2 | 0 | 0 | 0 | 0 | 0 |
| 2014 | 0 | 3 | 4 | 2 | 0 | 0 | 0 | 2 | 0 | 0 | 2 | 2 | 2 |
| 2014 | 1 | 0 | 0 | 1 | 0 | 4 | 0 | 0 | 0 | 0 | 0 | 0 | 0 |
| 2014 | 0 | 1 | 0 | 0 | 0 | 3 | 0 | 2 | 0 | 0 | 0 | 0 | 0 |
| 2014 | 0 | 0 | 3 | 1 | 0 | 1 | 0 | 2 | 0 | 1 | 0 | 0 | 0 |
| 2014 | 0 | 0 | 4 | 1 | 0 | 3 | 0 | 0 | 0 | 0 | 0 | 0 | 0 |
| 2014 | 0 | 3 | 1 | 1 | 1 | 0 | 0 | 2 | 0 | 1 | 0 | 0 | 1 |
| 2014 | 0 | 4 | 1 | 1 | 0 | 1 | 0 | 2 | 0 | 1 | 0 | 0 | 0 |
| 2014 | 0 | 0 | 2 | 0 | 0 | 3 | 0 | 0 | 0 | 0 | 0 | 0 | 1 |
| 2014 | 1 | 3 | 3 | 1 | 0 | 1 | 0 | 0 | 0 | 1 | 0 | 0 | 0 |
| 2014 | 1 | 3 | 3 | 1 | 0 | 3 | 0 | 0 | 0 | 1 | 0 | 0 | 0 |
| 2014 | 1 | 2 | 0 | 1 | 0 | 3 | 0 | 0 | 0 | 0 | 0 | 0 | 0 |
| 2014 | 0 | 4 | 1 | 1 | 1 | 0 | 0 | 0 | 0 | 0 | 0 | 0 | 0 |
| 2014 | 0 | 4 | 4 | 2 | 0 | 0 | 0 | 0 | 0 | 1 | 0 | 0 | 0 |
| 2014 | 1 | 2 | 1 | 1 | 1 | 0 | 0 | 2 | 1 | 1 | 0 | 0 | 0 |
| 2014 | 0 | 3 | 0 | 1 | 0 | 2 | 0 | 0 | 0 | 1 | 0 | 0 | 0 |
| 2014 | 0 | 1 | 1 | 2 | 1 | 2 | 0 | 0 | 0 | 1 | 0 | 0 | 0 |
| 2014 | 0 | 3 | 1 | 1 | 1 | 0 | 0 | 0 | 1 | 1 | 0 | 0 | 0 |
| 2014 | 0 | 3 | 1 | 0 | 0 | 2 | 0 | 1 | 0 | 1 | 0 | 0 | 0 |
| 2014 | 1 | 3 | 0 | 1 | 0 | 0 | 0 | 2 | 0 | 1 | 0 | 0 | 0 |
| 2014 | 0 | 0 | 0 | 1 | 0 | 3 | 0 | 0 | 0 | 1 | 0 | 0 | 0 |
| 2014 | 1 | 0 | 4 | 1 | 0 | 4 | 0 | 2 | 0 | 2 | 2 | 2 | 2 |
| 2014 | 0 | 3 | 4 | 1 | 1 | 3 | 0 | 2 | 0 | 2 | 2 | 2 | 2 |
| 2014 | 1 | 2 | 4 | 1 | 0 | 4 | 0 | 2 | 0 | 2 | 2 | 2 | 2 |
| 2014 | 0 | 1 | 4 | 2 | 0 | 1 | 0 | 2 | 0 | 2 | 2 | 2 | 2 |
| 2014 | 1 | 1 | 4 | 1 | 0 | 1 | 0 | 2 | 0 | 2 | 2 | 2 | 2 |
| 2014 | 0 | 0 | 4 | 2 | 0 | 2 | 0 | 2 | 0 | 2 | 2 | 2 | 2 |
| 2014 | 1 | 2 | 4 | 1 | 0 | 2 | 0 | 2 | 0 | 2 | 2 | 2 | 2 |
| 2014 | 1 | 3 | 4 | 1 | 0 | 4 | 0 | 2 | 0 | 2 | 2 | 2 | 2 |
| 2014 | 1 | 1 | 4 | 2 | 0 | 3 | 1 | 2 | 0 | 2 | 2 | 2 | 2 |
| 2014 | 1 | 2 | 4 | 2 | 0 | 1 | 0 | 2 | 0 | 2 | 2 | 2 | 2 |
| 2014 | 1 | 2 | 4 | 1 | 0 | 3 | 0 | 2 | 0 | 2 | 2 | 2 | 2 |
| 2014 | 0 | 0 | 4 | 2 | 0 | 1 | 0 | 2 | 0 | 2 | 2 | 2 | 2 |
| 2014 | 0 | 0 | 4 | 1 | 1 | 4 | 1 | 2 | 0 | 2 | 2 | 2 | 2 |
| 2014 | 1 | 2 | 4 | 1 | 1 | 0 | 0 | 2 | 0 | 2 | 2 | 2 | 2 |
| 2014 | 0 | 3 | 4 | 2 | 1 | 0 | 0 | 2 | 1 | 2 | 2 | 2 | 2 |
| 2014 | 1 | 0 | 4 | 2 | 0 | 1 | 0 | 2 | 0 | 2 | 2 | 2 | 2 |
| 2014 | 0 | 0 | 4 | 2 | 1 | 2 | 0 | 2 | 0 | 2 | 2 | 2 | 2 |
| 2014 | 1 | 1 | 4 | 2 | 1 | 0 | 0 | 2 | 0 | 2 | 2 | 2 | 2 |
| 2014 | 1 | 2 | 4 | 2 | 0 | 2 | 0 | 2 | 0 | 2 | 2 | 2 | 2 |
| 2014 | 1 | 2 | 4 | 1 | 0 | 3 | 0 | 2 | 0 | 2 | 2 | 2 | 2 |
| 2014 | 1 | 1 | 4 | 2 | 1 | 2 | 0 | 2 | 0 | 2 | 2 | 2 | 2 |
| 2014 | 0 | 1 | 4 | 1 | 1 | 0 | 0 | 2 | 0 | 2 | 2 | 2 | 2 |
| 2014 | 1 | 2 | 4 | 1 | 2 | 1 | 0 | 2 | 0 | 2 | 2 | 2 | 2 |
| 2014 | 1 | 2 | 4 | 2 | 0 | 3 | 0 | 2 | 0 | 2 | 2 | 2 | 2 |
| 2014 | 1 | 0 | 4 | 2 | 1 | 3 | 0 | 2 | 0 | 2 | 2 | 2 | 2 |
| 2014 | 1 | 2 | 4 | 1 | 0 | 4 | 0 | 2 | 0 | 2 | 2 | 2 | 2 |
| 2014 | 1 | 3 | 4 | 1 | 1 | 0 | 0 | 2 | 1 | 2 | 2 | 2 | 2 |
| 2014 | 1 | 2 | 4 | 1 | 0 | 2 | 0 | 2 | 0 | 2 | 2 | 2 | 2 |
| 2014 | 0 | 2 | 4 | 2 | 0 | 3 | 0 | 2 | 0 | 2 | 2 | 2 | 2 |
| 2014 | 0 | 0 | 4 | 2 | 0 | 4 | 0 | 2 | 0 | 2 | 2 | 2 | 2 |
| 2014 | 0 | 3 | 4 | 1 | 1 | 1 | 0 | 2 | 0 | 2 | 2 | 2 | 2 |
| 2014 | 1 | 1 | 4 | 1 | 0 | 1 | 0 | 2 | 0 | 2 | 2 | 2 | 2 |
| 2014 | 1 | 1 | 4 | 2 | 0 | 2 | 0 | 2 | 0 | 2 | 2 | 2 | 2 |
| 2014 | 0 | 1 | 4 | 2 | 0 | 1 | 0 | 2 | 0 | 2 | 2 | 2 | 2 |
| 2014 | 1 | 3 | 4 | 1 | 0 | 3 | 0 | 2 | 0 | 2 | 2 | 2 | 2 |
| 2014 | 1 | 2 | 4 | 2 | 1 | 3 | 1 | 2 | 0 | 2 | 2 | 2 | 2 |
| 2014 | 1 | 1 | 4 | 1 | 1 | 0 | 0 | 2 | 0 | 2 | 2 | 2 | 2 |
| 2014 | 0 | 3 | 4 | 2 | 0 | 3 | 0 | 2 | 0 | 2 | 2 | 2 | 2 |
| 2014 | 0 | 0 | 4 | 2 | 0 | 2 | 0 | 2 | 0 | 2 | 2 | 2 | 2 |
| 2014 | 1 | 3 | 4 | 1 | 0 | 4 | 0 | 2 | 0 | 2 | 2 | 2 | 2 |
| 2014 | 1 | 1 | 4 | 2 | 0 | 2 | 0 | 2 | 0 | 2 | 2 | 2 | 2 |
| 2014 | 1 | 1 | 4 | 2 | 0 | 2 | 0 | 2 | 0 | 2 | 2 | 2 | 2 |
| 2014 | 0 | 0 | 4 | 1 | 0 | 4 | 0 | 2 | 0 | 2 | 2 | 2 | 2 |
| 2014 | 1 | 1 | 4 | 2 | 1 | 0 | 0 | 2 | 0 | 2 | 2 | 2 | 2 |
| 2014 | 0 | 0 | 4 | 1 | 1 | 3 | 0 | 2 | 0 | 2 | 2 | 2 | 2 |
| 2014 | 0 | 0 | 4 | 1 | 1 | 1 | 0 | 2 | 0 | 2 | 2 | 2 | 2 |
| 2014 | 0 | 1 | 4 | 2 | 1 | 1 | 0 | 2 | 0 | 2 | 2 | 2 | 2 |
| 2014 | 1 | 1 | 4 | 2 | 1 | 1 | 0 | 2 | 0 | 2 | 2 | 2 | 2 |
| 2014 | 1 | 2 | 4 | 2 | 1 | 1 | 0 | 1 | 0 | 2 | 2 | 2 | 2 |
| 2014 | 1 | 0 | 4 | 1 | 0 | 4 | 0 | 2 | 0 | 2 | 2 | 2 | 2 |
| 2014 | 0 | 1 | 4 | 1 | 0 | 2 | 0 | 2 | 0 | 2 | 2 | 2 | 2 |
| 2014 | 0 | 0 | 0 | 0 | 0 | 3 | 1 | 1 | 0 | 2 | 2 | 2 | 2 |
| 2014 | 1 | 0 | 0 | 1 | 1 | 2 | 0 | 0 | 0 | 0 | 0 | 0 | 0 |
| 2014 | 0 | 2 | 1 | 0 | 0 | 2 | 0 | 0 | 0 | 1 | 2 | 2 | 2 |
| 2014 | 0 | 1 | 0 | 1 | 0 | 3 | 0 | 0 | 0 | 0 | 0 | 1 | 1 |
| 2014 | 1 | 3 | 2 | 1 | 1 | 3 | 0 | 0 | 1 | 1 | 0 | 0 | 0 |
| 2014 | 1 | 4 | 4 | 2 | 2 | 4 | 0 | 2 | 0 | 0 | 0 | 0 | 0 |
| 2014 | 0 | 1 | 4 | 1 | 0 | 2 | 0 | 0 | 0 | 0 | 0 | 0 | 0 |
| 2014 | 1 | 1 | 2 | 1 | 0 | 2 | 0 | 0 | 0 | 1 | 0 | 0 | 0 |
| 2014 | 1 | 4 | 4 | 2 | 0 | 0 | 0 | 2 | 0 | 2 | 0 | 0 | 0 |
| 2014 | 0 | 1 | 1 | 1 | 1 | 3 | 0 | 0 | 0 | 1 | 0 | 0 | 0 |
| 2014 | 0 | 1 | 3 | 1 | 0 | 2 | 0 | 0 | 0 | 0 | 0 | 0 | 1 |
| 2014 | 0 | 4 | 4 | 2 | 0 | 2 | 0 | 2 | 0 | 2 | 0 | 0 | 0 |
| 2014 | 0 | 0 | 0 | 1 | 0 | 2 | 0 | 0 | 0 | 1 | 0 | 0 | 0 |
| 2014 | 1 | 3 | 0 | 1 | 0 | 4 | 0 | 0 | 0 | 0 | 0 | 0 | 0 |
| 2014 | 1 | 1 | 4 | 1 | 0 | 3 | 0 | 0 | 0 | 2 | 0 | 0 | 0 |
| 2014 | 1 | 2 | 0 | 1 | 1 | 3 | 0 | 2 | 0 | 0 | 0 | 0 | 0 |
| 2014 | 1 | 1 | 1 | 1 | 0 | 3 | 0 | 2 | 0 | 0 | 2 | 2 | 2 |
| 2014 | 1 | 1 | 4 | 0 | 1 | 1 | 0 | 0 | 0 | 0 | 0 | 0 | 0 |
| 2014 | 0 | 3 | 2 | 1 | 1 | 1 | 0 | 2 | 0 | 1 | 0 | 0 | 0 |
| 2014 | 1 | 3 | 4 | 1 | 1 | 0 | 0 | 1 | 0 | 1 | 0 | 0 | 0 |
| 2014 | 0 | 1 | 0 | 1 | 1 | 3 | 0 | 0 | 0 | 0 | 0 | 0 | 0 |
| 2014 | 1 | 4 | 4 | 2 | 0 | 1 | 0 | 2 | 0 | 0 | 0 | 0 | 0 |
| 2014 | 1 | 3 | 2 | 1 | 1 | 4 | 0 | 2 | 0 | 0 | 0 | 0 | 0 |
| 2014 | 0 | 1 | 0 | 1 | 0 | 3 | 0 | 0 | 0 | 0 | 0 | 0 | 0 |
| 2014 | 1 | 3 | 0 | 1 | 1 | 2 | 0 | 0 | 0 | 0 | 0 | 0 | 0 |
| 2014 | 1 | 2 | 1 | 0 | 1 | 0 | 0 | 2 | 1 | 0 | 0 | 0 | 0 |
| 2014 | 1 | 2 | 2 | 1 | 0 | 2 | 0 | 0 | 0 | 1 | 0 | 0 | 0 |
| 2014 | 1 | 1 | 1 | 1 | 0 | 3 | 0 | 0 | 0 | 1 | 0 | 0 | 0 |
| 2014 | 0 | 0 | 4 | 1 | 0 | 1 | 0 | 0 | 0 | 0 | 2 | 2 | 2 |
| 2014 | 0 | 0 | 4 | 1 | 0 | 2 | 0 | 0 | 0 | 0 | 0 | 0 | 0 |
| 2014 | 0 | 3 | 2 | 2 | 1 | 1 | 0 | 2 | 0 | 1 | 2 | 2 | 2 |
| 2014 | 0 | 1 | 2 | 1 | 0 | 2 | 0 | 2 | 0 | 1 | 0 | 0 | 0 |
| 2014 | 0 | 3 | 4 | 1 | 0 | 2 | 0 | 0 | 0 | 0 | 0 | 0 | 0 |
| 2014 | 0 | 0 | 1 | 1 | 0 | 3 | 0 | 0 | 0 | 0 | 0 | 0 | 0 |
| 2014 | 1 | 2 | 3 | 1 | 0 | 3 | 0 | 0 | 0 | 0 | 2 | 2 | 2 |
| 2014 | 0 | 1 | 4 | 2 | 0 | 2 | 0 | 0 | 0 | 1 | 0 | 0 | 0 |
| 2014 | 0 | 1 | 1 | 1 | 0 | 3 | 0 | 0 | 0 | 1 | 0 | 0 | 0 |
| 2014 | 0 | 1 | 4 | 1 | 0 | 1 | 0 | 1 | 0 | 0 | 0 | 0 | 0 |
| 2014 | 1 | 3 | 1 | 2 | 1 | 2 | 0 | 2 | 0 | 2 | 2 | 2 | 2 |
| 2014 | 1 | 0 | 1 | 2 | 0 | 2 | 0 | 0 | 0 | 1 | 0 | 0 | 0 |
| 2014 | 1 | 3 | 0 | 0 | 0 | 3 | 0 | 0 | 0 | 0 | 0 | 0 | 0 |
| 2014 | 0 | 2 | 1 | 1 | 1 | 0 | 0 | 0 | 0 | 0 | 2 | 2 | 2 |
| 2014 | 1 | 3 | 4 | 1 | 0 | 3 | 0 | 0 | 0 | 1 | 0 | 0 | 0 |
| 2014 | 0 | 4 | 4 | 1 | 2 | 3 | 0 | 0 | 0 | 0 | 0 | 0 | 0 |
| 2014 | 1 | 3 | 2 | 2 | 0 | 2 | 0 | 1 | 0 | 0 | 2 | 2 | 2 |
| 2014 | 0 | 4 | 4 | 1 | 1 | 3 | 0 | 0 | 0 | 0 | 0 | 0 | 0 |
| 2014 | 0 | 0 | 1 | 1 | 0 | 1 | 0 | 0 | 0 | 1 | 0 | 0 | 1 |
| 2014 | 0 | 0 | 1 | 1 | 0 | 4 | 0 | 0 | 0 | 0 | 0 | 0 | 0 |
| 2014 | 1 | 3 | 1 | 1 | 1 | 2 | 0 | 0 | 0 | 0 | 0 | 0 | 0 |
| 2014 | 1 | 1 | 0 | 1 | 1 | 1 | 0 | 2 | 2 | 0 | 0 | 0 | 0 |
| 2014 | 1 | 1 | 1 | 0 | 0 | 0 | 0 | 0 | 0 | 1 | 0 | 0 | 0 |
| 2014 | 0 | 3 | 3 | 2 | 0 | 1 | 0 | 2 | 0 | 0 | 2 | 2 | 2 |
| 2014 | 0 | 3 | 4 | 1 | 2 | 4 | 0 | 0 | 0 | 2 | 0 | 0 | 0 |
| 2014 | 1 | 2 | 2 | 1 | 0 | 1 | 0 | 0 | 0 | 1 | 0 | 0 | 0 |
| 2014 | 1 | 2 | 1 | 0 | 0 | 3 | 0 | 0 | 0 | 1 | 0 | 0 | 0 |
| 2014 | 1 | 3 | 1 | 1 | 0 | 1 | 0 | 0 | 0 | 1 | 2 | 2 | 2 |
| 2014 | 0 | 3 | 3 | 1 | 0 | 0 | 0 | 0 | 0 | 1 | 0 | 0 | 0 |
| 2014 | 1 | 0 | 1 | 0 | 0 | 1 | 0 | 0 | 0 | 1 | 0 | 0 | 0 |
| 2014 | 0 | 0 | 1 | 1 | 0 | 0 | 0 | 0 | 0 | 1 | 0 | 0 | 0 |
| 2014 | 0 | 0 | 1 | 1 | 0 | 1 | 0 | 2 | 0 | 1 | 0 | 0 | 0 |
| 2014 | 1 | 0 | 1 | 1 | 0 | 2 | 0 | 1 | 0 | 1 | 0 | 0 | 0 |
| 2014 | 0 | 3 | 2 | 0 | 1 | 2 | 0 | 0 | 0 | 1 | 0 | 0 | 0 |
| 2014 | 1 | 4 | 0 | 0 | 0 | 1 | 0 | 0 | 0 | 0 | 0 | 0 | 0 |
| 2014 | 0 | 1 | 2 | 1 | 0 | 1 | 0 | 0 | 0 | 1 | 0 | 0 | 0 |
| 2014 | 1 | 0 | 0 | 1 | 1 | 2 | 0 | 0 | 0 | 0 | 0 | 0 | 0 |
| 2014 | 1 | 1 | 2 | 1 | 0 | 3 | 0 | 0 | 0 | 0 | 0 | 0 | 0 |
| 2014 | 1 | 0 | 0 | 1 | 0 | 4 | 0 | 0 | 0 | 0 | 0 | 0 | 0 |
| 2014 | 0 | 0 | 2 | 1 | 0 | 3 | 0 | 2 | 0 | 1 | 2 | 2 | 2 |
| 2014 | 1 | 3 | 4 | 1 | 0 | 2 | 0 | 0 | 0 | 1 | 0 | 0 | 0 |
| 2014 | 0 | 1 | 2 | 0 | 0 | 1 | 0 | 0 | 0 | 0 | 0 | 0 | 0 |
| 2014 | 0 | 3 | 2 | 1 | 2 | 3 | 0 | 1 | 0 | 0 | 0 | 0 | 0 |
| 2014 | 0 | 1 | 2 | 1 | 0 | 2 | 0 | 0 | 0 | 1 | 0 | 0 | 0 |
| 2014 | 0 | 3 | 0 | 1 | 0 | 2 | 0 | 0 | 0 | 0 | 0 | 0 | 0 |
| 2014 | 0 | 1 | 1 | 0 | 1 | 2 | 0 | 0 | 0 | 1 | 2 | 2 | 2 |
| 2014 | 0 | 2 | 3 | 1 | 0 | 2 | 0 | 0 | 0 | 0 | 0 | 0 | 0 |
| 2014 | 0 | 1 | 1 | 1 | 0 | 0 | 0 | 0 | 0 | 0 | 0 | 0 | 0 |
| 2014 | 1 | 4 | 4 | 2 | 0 | 3 | 0 | 2 | 2 | 0 | 2 | 2 | 2 |
| 2014 | 0 | 1 | 2 | 1 | 0 | 1 | 0 | 2 | 0 | 0 | 0 | 0 | 0 |
| 2014 | 0 | 3 | 0 | 1 | 1 | 0 | 0 | 0 | 0 | 0 | 0 | 0 | 0 |
| 2014 | 1 | 2 | 2 | 1 | 0 | 2 | 0 | 0 | 0 | 1 | 0 | 0 | 0 |
| 2014 | 1 | 1 | 1 | 1 | 0 | 0 | 0 | 0 | 0 | 1 | 0 | 0 | 0 |
| 2014 | 1 | 0 | 1 | 1 | 0 | 3 | 0 | 0 | 0 | 1 | 0 | 0 | 0 |
| 2014 | 0 | 2 | 1 | 1 | 0 | 3 | 0 | 0 | 0 | 2 | 0 | 0 | 0 |
| 2014 | 1 | 2 | 1 | 1 | 0 | 4 | 0 | 0 | 0 | 0 | 0 | 0 | 0 |
| 2014 | 0 | 3 | 4 | 2 | 1 | 1 | 0 | 2 | 0 | 0 | 2 | 2 | 2 |
| 2014 | 0 | 1 | 1 | 1 | 1 | 2 | 0 | 1 | 0 | 1 | 0 | 0 | 0 |
| 2014 | 1 | 3 | 2 | 1 | 0 | 2 | 1 | 2 | 0 | 1 | 0 | 0 | 0 |
| 2014 | 0 | 0 | 1 | 0 | 0 | 1 | 0 | 2 | 0 | 0 | 0 | 0 | 0 |
| 2014 | 0 | 3 | 1 | 1 | 0 | 1 | 0 | 0 | 0 | 0 | 0 | 0 | 0 |
| 2014 | 0 | 0 | 0 | 1 | 1 | 4 | 0 | 0 | 0 | 0 | 0 | 0 | 0 |
| 2014 | 0 | 1 | 0 | 1 | 0 | 2 | 0 | 0 | 0 | 0 | 2 | 2 | 2 |
| 2014 | 0 | 4 | 4 | 2 | 2 | 1 | 0 | 2 | 2 | 2 | 2 | 2 | 2 |
| 2014 | 0 | 0 | 1 | 1 | 0 | 2 | 0 | 0 | 0 | 0 | 0 | 0 | 0 |
| 2014 | 1 | 1 | 1 | 1 | 0 | 4 | 0 | 0 | 0 | 1 | 2 | 2 | 2 |
| 2014 | 0 | 0 | 0 | 1 | 1 | 3 | 0 | 2 | 0 | 0 | 0 | 0 | 0 |
| 2014 | 1 | 3 | 2 | 1 | 1 | 2 | 0 | 0 | 0 | 1 | 0 | 0 | 0 |
| 2014 | 0 | 0 | 0 | 1 | 0 | 2 | 0 | 0 | 0 | 0 | 2 | 2 | 2 |
| 2014 | 0 | 2 | 0 | 1 | 1 | 0 | 0 | 0 | 0 | 0 | 2 | 2 | 2 |
| 2014 | 1 | 1 | 0 | 1 | 0 | 0 | 0 | 0 | 0 | 0 | 2 | 2 | 2 |
| 2014 | 1 | 1 | 2 | 1 | 0 | 1 | 0 | 0 | 0 | 1 | 0 | 0 | 0 |
| 2014 | 1 | 3 | 0 | 1 | 1 | 4 | 0 | 0 | 0 | 0 | 0 | 0 | 1 |
| 2014 | 1 | 3 | 0 | 1 | 0 | 3 | 0 | 2 | 0 | 0 | 0 | 0 | 0 |
| 2014 | 0 | 3 | 1 | 1 | 0 | 3 | 0 | 2 | 0 | 0 | 0 | 0 | 0 |
| 2014 | 1 | 1 | 2 | 0 | 1 | 0 | 1 | 2 | 0 | 1 | 0 | 0 | 0 |
| 2014 | 1 | 4 | 1 | 0 | 0 | 0 | 0 | 2 | 0 | 1 | 2 | 2 | 2 |
| 2014 | 0 | 3 | 1 | 1 | 1 | 3 | 0 | 0 | 0 | 0 | 0 | 0 | 0 |
| 2014 | 0 | 1 | 1 | 0 | 1 | 1 | 0 | 0 | 0 | 0 | 2 | 2 | 2 |
| 2014 | 0 | 2 | 1 | 2 | 0 | 3 | 0 | 2 | 0 | 0 | 1 | 0 | 0 |
| 2014 | 0 | 4 | 4 | 2 | 1 | 3 | 0 | 0 | 0 | 0 | 0 | 1 | 0 |
| 2014 | 1 | 2 | 1 | 1 | 0 | 2 | 0 | 2 | 0 | 1 | 0 | 1 | 0 |
| 2014 | 1 | 2 | 1 | 1 | 1 | 2 | 0 | 0 | 0 | 1 | 2 | 2 | 2 |
| 2014 | 0 | 4 | 4 | 2 | 1 | 2 | 0 | 0 | 0 | 0 | 2 | 2 | 2 |
| 2014 | 1 | 3 | 3 | 1 | 2 | 2 | 0 | 2 | 0 | 2 | 0 | 0 | 0 |
| 2014 | 0 | 1 | 0 | 2 | 0 | 1 | 0 | 2 | 0 | 0 | 2 | 2 | 2 |
| 2014 | 0 | 0 | 4 | 0 | 0 | 1 | 0 | 0 | 0 | 2 | 2 | 2 | 2 |
| 2014 | 0 | 3 | 1 | 0 | 1 | 1 | 0 | 1 | 0 | 1 | 0 | 0 | 0 |
| 2014 | 0 | 1 | 3 | 1 | 0 | 1 | 0 | 2 | 0 | 1 | 2 | 2 | 2 |
| 2014 | 1 | 3 | 3 | 1 | 0 | 0 | 0 | 0 | 0 | 1 | 2 | 2 | 2 |
| 2014 | 0 | 4 | 4 | 1 | 0 | 3 | 0 | 0 | 0 | 0 | 0 | 0 | 0 |
| 2014 | 0 | 1 | 0 | 2 | 1 | 1 | 0 | 2 | 0 | 1 | 0 | 0 | 0 |
| 2014 | 1 | 2 | 1 | 2 | 1 | 0 | 0 | 0 | 1 | 1 | 0 | 0 | 0 |
| 2014 | 0 | 2 | 0 | 0 | 1 | 2 | 0 | 1 | 0 | 0 | 2 | 2 | 2 |
| 2014 | 0 | 3 | 3 | 1 | 0 | 1 | 0 | 0 | 0 | 0 | 0 | 0 | 0 |
| 2014 | 0 | 1 | 4 | 1 | 1 | 3 | 0 | 0 | 0 | 2 | 0 | 0 | 0 |
| 2014 | 1 | 2 | 2 | 0 | 0 | 2 | 0 | 0 | 0 | 1 | 2 | 2 | 2 |
| 2014 | 1 | 0 | 1 | 1 | 0 | 1 | 0 | 0 | 0 | 1 | 0 | 0 | 0 |
| 2014 | 1 | 1 | 1 | 1 | 1 | 0 | 0 | 0 | 0 | 1 | 0 | 0 | 0 |
| 2014 | 0 | 4 | 4 | 1 | 0 | 4 | 0 | 0 | 0 | 2 | 2 | 2 | 2 |
| 2014 | 1 | 2 | 2 | 1 | 1 | 0 | 0 | 0 | 1 | 0 | 0 | 1 | 0 |
| 2014 | 0 | 1 | 0 | 0 | 1 | 3 | 0 | 0 | 0 | 0 | 2 | 2 | 2 |
| 2014 | 0 | 0 | 3 | 1 | 0 | 2 | 0 | 1 | 0 | 0 | 2 | 2 | 2 |
| 2014 | 1 | 1 | 2 | 1 | 0 | 2 | 0 | 0 | 0 | 1 | 0 | 1 | 0 |
| 2014 | 0 | 0 | 4 | 0 | 0 | 2 | 0 | 0 | 0 | 0 | 2 | 2 | 2 |
| 2014 | 0 | 3 | 3 | 1 | 0 | 0 | 0 | 2 | 0 | 1 | 2 | 2 | 2 |
| 2014 | 0 | 1 | 4 | 1 | 0 | 1 | 0 | 0 | 0 | 0 | 2 | 2 | 2 |
| 2014 | 1 | 3 | 2 | 0 | 1 | 0 | 0 | 0 | 2 | 1 | 2 | 2 | 2 |
| 2014 | 0 | 4 | 1 | 1 | 0 | 1 | 0 | 0 | 0 | 1 | 2 | 2 | 2 |
| 2014 | 1 | 3 | 4 | 1 | 0 | 3 | 0 | 0 | 0 | 0 | 0 | 0 | 0 |
| 2014 | 0 | 1 | 4 | 1 | 0 | 0 | 0 | 0 | 0 | 0 | 2 | 2 | 2 |
| 2014 | 1 | 0 | 0 | 1 | 1 | 3 | 0 | 0 | 0 | 0 | 2 | 2 | 2 |
| 2015 | 0 | 0 | 0 | 1 | 0 | 4 | 0 | 2 | 0 | 1 | 0 | 0 | 0 |
| 2015 | 0 | 1 | 1 | 1 | 0 | 0 | 0 | 2 | 0 | 1 | 0 | 0 | 0 |
| 2015 | 1 | 0 | 2 | 1 | 0 | 3 | 0 | 0 | 0 | 0 | 0 | 0 | 0 |
| 2015 | 1 | 3 | 3 | 1 | 0 | 1 | 0 | 0 | 0 | 1 | 0 | 0 | 0 |
| 2015 | 0 | 0 | 0 | 1 | 0 | 0 | 0 | 0 | 0 | 1 | 0 | 0 | 0 |
| 2015 | 0 | 2 | 2 | 1 | 0 | 2 | 0 | 0 | 0 | 1 | 0 | 0 | 0 |
| 2015 | 0 | 1 | 0 | 1 | 0 | 4 | 0 | 0 | 0 | 0 | 0 | 1 | 0 |
| 2015 | 1 | 1 | 0 | 2 | 1 | 0 | 0 | 0 | 0 | 0 | 0 | 0 | 0 |
| 2015 | 0 | 4 | 4 | 1 | 0 | 3 | 0 | 0 | 0 | 0 | 0 | 0 | 0 |
| 2015 | 0 | 1 | 4 | 2 | 2 | 3 | 0 | 0 | 0 | 0 | 0 | 0 | 1 |
| 2015 | 0 | 0 | 4 | 0 | 2 | 3 | 0 | 2 | 0 | 0 | 0 | 0 | 0 |
| 2015 | 0 | 0 | 0 | 1 | 1 | 1 | 0 | 0 | 0 | 1 | 0 | 0 | 0 |
| 2015 | 0 | 1 | 2 | 1 | 0 | 2 | 0 | 2 | 0 | 1 | 0 | 0 | 0 |
| 2015 | 1 | 1 | 0 | 1 | 0 | 1 | 0 | 0 | 0 | 1 | 0 | 1 | 0 |
| 2015 | 1 | 3 | 1 | 0 | 1 | 1 | 0 | 2 | 0 | 1 | 0 | 0 | 0 |
| 2015 | 0 | 0 | 0 | 1 | 1 | 3 | 0 | 0 | 0 | 0 | 0 | 0 | 0 |
| 2015 | 1 | 3 | 1 | 1 | 0 | 2 | 0 | 0 | 0 | 1 | 0 | 0 | 0 |
| 2015 | 0 | 3 | 3 | 1 | 0 | 1 | 0 | 0 | 0 | 1 | 0 | 0 | 0 |
| 2015 | 0 | 1 | 1 | 1 | 1 | 1 | 0 | 0 | 0 | 1 | 0 | 0 | 0 |
| 2015 | 1 | 3 | 2 | 1 | 0 | 2 | 0 | 0 | 0 | 1 | 0 | 1 | 0 |
| 2015 | 1 | 3 | 1 | 1 | 0 | 1 | 0 | 0 | 0 | 1 | 0 | 0 | 1 |
| 2015 | 1 | 1 | 3 | 1 | 0 | 3 | 1 | 0 | 0 | 0 | 0 | 0 | 0 |
| 2015 | 1 | 4 | 4 | 1 | 0 | 2 | 1 | 0 | 0 | 1 | 0 | 0 | 0 |
| 2015 | 0 | 3 | 1 | 0 | 0 | 3 | 1 | 2 | 0 | 0 | 0 | 0 | 0 |
| 2015 | 1 | 3 | 0 | 2 | 1 | 0 | 1 | 2 | 1 | 1 | 2 | 2 | 2 |
| 2015 | 1 | 1 | 1 | 0 | 1 | 2 | 1 | 2 | 0 | 1 | 0 | 0 | 0 |
| 2015 | 0 | 1 | 0 | 1 | 0 | 2 | 1 | 0 | 0 | 1 | 0 | 0 | 0 |
| 2015 | 1 | 1 | 4 | 1 | 0 | 4 | 1 | 0 | 0 | 0 | 0 | 0 | 0 |
| 2015 | 0 | 0 | 1 | 1 | 0 | 2 | 1 | 1 | 0 | 0 | 0 | 0 | 0 |
| 2015 | 1 | 1 | 1 | 1 | 0 | 2 | 1 | 0 | 0 | 1 | 0 | 0 | 0 |
| 2015 | 1 | 1 | 2 | 1 | 0 | 2 | 1 | 0 | 0 | 1 | 0 | 0 | 0 |
| 2015 | 1 | 2 | 1 | 1 | 0 | 2 | 1 | 0 | 0 | 1 | 0 | 1 | 0 |
| 2015 | 1 | 1 | 2 | 1 | 0 | 2 | 1 | 0 | 0 | 1 | 0 | 0 | 0 |
| 2015 | 1 | 2 | 2 | 1 | 0 | 2 | 1 | 1 | 0 | 0 | 0 | 0 | 0 |
| 2015 | 0 | 2 | 3 | 1 | 0 | 2 | 0 | 0 | 0 | 1 | 0 | 0 | 0 |
| 2015 | 1 | 1 | 4 | 1 | 0 | 4 | 0 | 0 | 0 | 0 | 2 | 2 | 2 |
| 2015 | 0 | 4 | 4 | 1 | 1 | 4 | 0 | 0 | 0 | 0 | 0 | 0 | 0 |
| 2015 | 1 | 0 | 1 | 1 | 0 | 4 | 0 | 0 | 0 | 0 | 0 | 0 | 0 |
| 2015 | 0 | 1 | 1 | 1 | 1 | 3 | 0 | 0 | 0 | 1 | 0 | 0 | 0 |
| 2015 | 1 | 3 | 1 | 1 | 1 | 4 | 0 | 2 | 0 | 0 | 0 | 0 | 0 |
| 2015 | 1 | 3 | 1 | 1 | 0 | 2 | 0 | 0 | 0 | 1 | 0 | 0 | 0 |
| 2015 | 1 | 1 | 1 | 1 | 0 | 2 | 0 | 0 | 0 | 1 | 0 | 0 | 0 |
| 2015 | 1 | 4 | 0 | 1 | 2 | 4 | 0 | 0 | 0 | 0 | 0 | 0 | 0 |
| 2015 | 0 | 1 | 1 | 2 | 0 | 3 | 0 | 0 | 0 | 0 | 0 | 0 | 0 |
| 2015 | 1 | 2 | 1 | 1 | 0 | 3 | 0 | 0 | 0 | 0 | 0 | 0 | 0 |
| 2015 | 0 | 1 | 0 | 0 | 0 | 0 | 0 | 0 | 0 | 2 | 0 | 0 | 0 |
| 2015 | 0 | 2 | 4 | 2 | 0 | 2 | 0 | 2 | 0 | 1 | 0 | 0 | 0 |
| 2015 | 0 | 0 | 2 | 1 | 0 | 2 | 0 | 0 | 0 | 1 | 0 | 0 | 0 |
| 2015 | 1 | 3 | 1 | 1 | 1 | 1 | 0 | 0 | 0 | 1 | 0 | 0 | 0 |
| 2015 | 0 | 0 | 0 | 0 | 0 | 3 | 0 | 2 | 0 | 0 | 0 | 0 | 0 |
| 2015 | 1 | 3 | 4 | 1 | 0 | 4 | 0 | 0 | 0 | 1 | 0 | 0 | 0 |
| 2015 | 1 | 1 | 0 | 1 | 0 | 3 | 0 | 2 | 0 | 0 | 0 | 0 | 0 |
| 2015 | 0 | 1 | 1 | 1 | 1 | 3 | 0 | 2 | 0 | 1 | 0 | 0 | 0 |
| 2015 | 1 | 4 | 4 | 0 | 1 | 1 | 0 | 0 | 0 | 1 | 2 | 2 | 2 |
| 2015 | 1 | 3 | 2 | 1 | 0 | 1 | 0 | 2 | 0 | 0 | 0 | 0 | 0 |
| 2015 | 1 | 0 | 0 | 1 | 0 | 2 | 0 | 0 | 0 | 2 | 0 | 0 | 0 |
| 2015 | 0 | 1 | 2 | 1 | 0 | 2 | 0 | 0 | 0 | 1 | 0 | 0 | 0 |
| 2015 | 1 | 0 | 1 | 1 | 0 | 2 | 0 | 2 | 0 | 1 | 0 | 1 | 0 |
| 2015 | 1 | 3 | 1 | 1 | 0 | 3 | 0 | 0 | 0 | 0 | 0 | 0 | 0 |
| 2015 | 1 | 3 | 3 | 1 | 2 | 3 | 0 | 0 | 0 | 1 | 0 | 0 | 0 |
| 2015 | 0 | 1 | 1 | 1 | 1 | 3 | 0 | 0 | 0 | 1 | 0 | 0 | 0 |
| 2015 | 0 | 2 | 1 | 1 | 0 | 3 | 0 | 0 | 0 | 0 | 0 | 0 | 0 |
| 2015 | 0 | 3 | 2 | 1 | 2 | 1 | 0 | 2 | 0 | 1 | 0 | 0 | 0 |
| 2015 | 0 | 3 | 0 | 1 | 0 | 3 | 0 | 2 | 0 | 1 | 0 | 0 | 0 |
| 2015 | 0 | 1 | 1 | 0 | 0 | 1 | 0 | 0 | 0 | 0 | 0 | 0 | 0 |
| 2015 | 1 | 2 | 3 | 1 | 0 | 2 | 0 | 0 | 0 | 1 | 0 | 1 | 0 |
| 2015 | 1 | 0 | 1 | 1 | 0 | 2 | 0 | 0 | 0 | 1 | 0 | 0 | 0 |
| 2015 | 1 | 3 | 2 | 1 | 0 | 4 | 0 | 0 | 0 | 0 | 0 | 0 | 0 |
| 2015 | 0 | 3 | 2 | 1 | 1 | 1 | 0 | 0 | 0 | 1 | 0 | 0 | 0 |
| 2015 | 0 | 3 | 2 | 1 | 1 | 3 | 0 | 2 | 0 | 0 | 0 | 0 | 0 |
| 2015 | 0 | 1 | 0 | 1 | 0 | 2 | 0 | 0 | 0 | 0 | 0 | 0 | 1 |
| 2015 | 0 | 0 | 0 | 1 | 0 | 2 | 0 | 0 | 0 | 0 | 0 | 0 | 0 |
| 2015 | 0 | 0 | 0 | 1 | 2 | 0 | 0 | 0 | 0 | 0 | 0 | 0 | 0 |
| 2015 | 1 | 1 | 0 | 0 | 0 | 2 | 0 | 0 | 0 | 0 | 0 | 0 | 0 |
| 2015 | 0 | 1 | 3 | 1 | 0 | 1 | 0 | 0 | 0 | 1 | 0 | 0 | 0 |
| 2015 | 0 | 2 | 1 | 1 | 0 | 1 | 0 | 0 | 0 | 1 | 0 | 0 | 0 |
| 2015 | 1 | 1 | 0 | 1 | 0 | 4 | 0 | 0 | 0 | 0 | 0 | 0 | 0 |
| 2015 | 0 | 2 | 0 | 1 | 0 | 2 | 0 | 1 | 0 | 1 | 0 | 0 | 0 |
| 2015 | 1 | 2 | 4 | 2 | 0 | 2 | 0 | 2 | 0 | 0 | 0 | 0 | 0 |
| 2015 | 0 | 1 | 1 | 1 | 1 | 2 | 0 | 2 | 0 | 1 | 0 | 0 | 0 |
| 2015 | 0 | 1 | 4 | 1 | 0 | 3 | 0 | 1 | 0 | 1 | 0 | 0 | 0 |
| 2015 | 1 | 1 | 0 | 1 | 0 | 2 | 0 | 0 | 0 | 0 | 0 | 0 | 0 |
| 2015 | 1 | 0 | 4 | 2 | 0 | 1 | 0 | 2 | 0 | 0 | 2 | 2 | 2 |
| 2015 | 0 | 3 | 4 | 1 | 0 | 4 | 0 | 0 | 0 | 0 | 0 | 0 | 0 |
| 2015 | 1 | 0 | 2 | 1 | 0 | 3 | 0 | 0 | 0 | 1 | 0 | 0 | 0 |
| 2015 | 1 | 2 | 0 | 0 | 1 | 0 | 0 | 1 | 0 | 0 | 0 | 0 | 0 |
| 2015 | 1 | 1 | 1 | 1 | 0 | 2 | 0 | 0 | 0 | 1 | 0 | 0 | 0 |
| 2015 | 1 | 1 | 1 | 1 | 0 | 3 | 0 | 2 | 0 | 0 | 0 | 0 | 0 |
| 2015 | 0 | 0 | 1 | 1 | 0 | 3 | 0 | 0 | 0 | 0 | 0 | 0 | 0 |
| 2015 | 0 | 0 | 0 | 1 | 0 | 3 | 0 | 0 | 0 | 0 | 0 | 0 | 0 |
| 2015 | 1 | 1 | 1 | 1 | 1 | 3 | 0 | 0 | 0 | 1 | 0 | 0 | 0 |
| 2015 | 1 | 1 | 1 | 1 | 0 | 2 | 0 | 0 | 0 | 1 | 0 | 0 | 0 |
| 2015 | 0 | 3 | 1 | 1 | 1 | 3 | 0 | 0 | 0 | 2 | 0 | 0 | 0 |
| 2015 | 0 | 3 | 3 | 1 | 0 | 2 | 0 | 0 | 0 | 1 | 0 | 0 | 0 |
| 2015 | 1 | 1 | 1 | 1 | 0 | 1 | 0 | 2 | 0 | 1 | 0 | 0 | 1 |
| 2015 | 0 | 0 | 1 | 1 | 0 | 3 | 0 | 2 | 0 | 1 | 0 | 0 | 0 |
| 2015 | 0 | 3 | 1 | 1 | 0 | 2 | 0 | 0 | 0 | 0 | 0 | 0 | 0 |
| 2015 | 1 | 3 | 0 | 1 | 1 | 1 | 0 | 2 | 0 | 0 | 0 | 0 | 0 |
| 2015 | 0 | 0 | 1 | 1 | 2 | 2 | 0 | 0 | 0 | 1 | 0 | 0 | 0 |
| 2015 | 0 | 2 | 2 | 1 | 0 | 3 | 0 | 0 | 0 | 1 | 0 | 0 | 0 |
| 2015 | 0 | 1 | 1 | 1 | 0 | 2 | 0 | 2 | 0 | 1 | 0 | 0 | 0 |
| 2015 | 0 | 1 | 1 | 1 | 0 | 3 | 0 | 0 | 0 | 1 | 0 | 0 | 0 |
| 2015 | 0 | 1 | 1 | 1 | 0 | 4 | 0 | 2 | 0 | 0 | 0 | 0 | 0 |
| 2015 | 1 | 2 | 1 | 1 | 0 | 2 | 0 | 0 | 0 | 1 | 0 | 0 | 0 |
| 2015 | 1 | 3 | 2 | 1 | 0 | 1 | 0 | 0 | 0 | 1 | 0 | 0 | 0 |
| 2015 | 1 | 1 | 4 | 1 | 0 | 1 | 0 | 0 | 0 | 1 | 0 | 0 | 0 |
| 2015 | 0 | 0 | 0 | 1 | 1 | 2 | 0 | 0 | 0 | 1 | 0 | 0 | 0 |
| 2015 | 0 | 1 | 4 | 1 | 0 | 3 | 0 | 0 | 0 | 0 | 0 | 0 | 0 |
| 2015 | 1 | 0 | 1 | 0 | 0 | 2 | 0 | 0 | 0 | 1 | 0 | 0 | 0 |
| 2015 | 1 | 1 | 1 | 1 | 1 | 1 | 0 | 0 | 0 | 1 | 0 | 0 | 0 |
| 2015 | 1 | 2 | 4 | 1 | 0 | 2 | 0 | 0 | 0 | 1 | 0 | 0 | 0 |
| 2015 | 1 | 3 | 3 | 1 | 0 | 2 | 0 | 2 | 0 | 1 | 0 | 0 | 0 |
| 2015 | 0 | 2 | 0 | 1 | 0 | 3 | 0 | 0 | 0 | 0 | 0 | 0 | 0 |
| 2015 | 1 | 3 | 3 | 1 | 2 | 2 | 0 | 0 | 0 | 1 | 0 | 0 | 0 |
| 2015 | 1 | 3 | 4 | 2 | 1 | 1 | 0 | 0 | 0 | 0 | 2 | 2 | 2 |
| 2015 | 0 | 3 | 1 | 1 | 1 | 2 | 0 | 0 | 0 | 1 | 0 | 0 | 0 |
| 2015 | 0 | 1 | 1 | 1 | 1 | 0 | 0 | 0 | 0 | 1 | 0 | 0 | 0 |
| 2015 | 0 | 3 | 4 | 1 | 0 | 1 | 0 | 0 | 0 | 0 | 0 | 0 | 0 |
| 2015 | 0 | 0 | 1 | 1 | 0 | 1 | 0 | 0 | 0 | 0 | 0 | 0 | 0 |
| 2015 | 1 | 3 | 1 | 1 | 0 | 3 | 0 | 0 | 0 | 1 | 0 | 0 | 0 |
| 2015 | 0 | 3 | 0 | 0 | 0 | 0 | 0 | 0 | 0 | 1 | 0 | 0 | 0 |
| 2015 | 1 | 3 | 2 | 0 | 0 | 1 | 0 | 0 | 0 | 1 | 0 | 0 | 0 |
| 2015 | 1 | 1 | 2 | 1 | 0 | 1 | 0 | 0 | 0 | 1 | 0 | 0 | 0 |
| 2015 | 0 | 1 | 3 | 1 | 0 | 3 | 0 | 0 | 0 | 1 | 0 | 0 | 0 |
| 2015 | 1 | 4 | 0 | 1 | 0 | 4 | 0 | 2 | 0 | 0 | 2 | 2 | 2 |
| 2015 | 0 | 1 | 0 | 1 | 0 | 1 | 0 | 0 | 0 | 0 | 0 | 0 | 0 |
| 2015 | 0 | 0 | 1 | 1 | 0 | 1 | 0 | 0 | 0 | 1 | 0 | 0 | 0 |
| 2015 | 0 | 1 | 4 | 2 | 0 | 1 | 0 | 0 | 0 | 1 | 0 | 0 | 0 |
| 2015 | 0 | 1 | 1 | 1 | 0 | 1 | 0 | 0 | 0 | 0 | 0 | 0 | 0 |
| 2015 | 0 | 0 | 2 | 1 | 2 | 1 | 0 | 0 | 0 | 0 | 0 | 0 | 0 |
| 2015 | 0 | 0 | 1 | 1 | 0 | 1 | 0 | 0 | 0 | 0 | 0 | 0 | 0 |
| 2015 | 0 | 0 | 1 | 1 | 0 | 3 | 0 | 2 | 0 | 0 | 0 | 0 | 0 |
| 2015 | 0 | 3 | 3 | 1 | 0 | 1 | 0 | 1 | 0 | 0 | 0 | 0 | 0 |
| 2015 | 0 | 3 | 1 | 1 | 0 | 2 | 0 | 1 | 0 | 0 | 0 | 0 | 0 |
| 2015 | 0 | 2 | 3 | 1 | 1 | 0 | 0 | 0 | 1 | 0 | 0 | 0 | 0 |
| 2015 | 0 | 1 | 1 | 1 | 1 | 1 | 0 | 0 | 0 | 1 | 0 | 0 | 1 |
| 2015 | 1 | 2 | 1 | 1 | 1 | 0 | 0 | 2 | 1 | 1 | 0 | 0 | 0 |
| 2015 | 1 | 0 | 1 | 1 | 0 | 3 | 0 | 2 | 0 | 1 | 0 | 0 | 0 |
| 2015 | 1 | 3 | 2 | 1 | 0 | 2 | 0 | 0 | 0 | 1 | 0 | 0 | 0 |
| 2015 | 1 | 3 | 1 | 1 | 0 | 3 | 0 | 0 | 0 | 0 | 0 | 0 | 0 |
| 2015 | 1 | 3 | 2 | 0 | 1 | 0 | 0 | 2 | 0 | 1 | 0 | 0 | 0 |
| 2015 | 0 | 3 | 4 | 1 | 1 | 0 | 0 | 1 | 0 | 1 | 0 | 0 | 0 |
| 2015 | 1 | 2 | 2 | 1 | 0 | 2 | 0 | 0 | 0 | 1 | 0 | 0 | 0 |
| 2015 | 0 | 3 | 0 | 1 | 1 | 0 | 0 | 2 | 0 | 0 | 0 | 0 | 0 |
| 2015 | 0 | 1 | 4 | 0 | 1 | 2 | 0 | 0 | 0 | 0 | 0 | 0 | 0 |
| 2015 | 0 | 2 | 0 | 1 | 0 | 2 | 0 | 2 | 0 | 1 | 0 | 0 | 0 |
| 2015 | 0 | 3 | 2 | 1 | 0 | 1 | 0 | 0 | 0 | 1 | 0 | 0 | 0 |
| 2015 | 1 | 1 | 3 | 1 | 0 | 1 | 0 | 0 | 0 | 1 | 0 | 0 | 0 |
| 2015 | 0 | 1 | 1 | 1 | 0 | 1 | 0 | 0 | 0 | 1 | 0 | 0 | 0 |
| 2015 | 1 | 2 | 2 | 1 | 1 | 0 | 0 | 1 | 0 | 0 | 0 | 0 | 0 |
| 2015 | 0 | 2 | 0 | 1 | 1 | 2 | 0 | 1 | 0 | 1 | 0 | 0 | 0 |
| 2015 | 0 | 1 | 1 | 0 | 0 | 2 | 0 | 0 | 0 | 0 | 0 | 0 | 1 |
| 2015 | 1 | 0 | 0 | 1 | 0 | 3 | 0 | 0 | 0 | 0 | 0 | 0 | 0 |
| 2015 | 0 | 2 | 3 | 1 | 1 | 0 | 0 | 0 | 0 | 1 | 0 | 0 | 0 |
| 2015 | 1 | 1 | 0 | 1 | 0 | 1 | 0 | 0 | 0 | 0 | 2 | 2 | 2 |
| 2015 | 0 | 0 | 0 | 0 | 2 | 2 | 0 | 0 | 0 | 1 | 0 | 0 | 0 |
| 2015 | 0 | 3 | 1 | 1 | 0 | 0 | 0 | 0 | 0 | 1 | 0 | 0 | 0 |
| 2015 | 0 | 1 | 2 | 1 | 0 | 1 | 0 | 2 | 0 | 0 | 0 | 0 | 0 |
| 2015 | 0 | 2 | 0 | 1 | 0 | 3 | 0 | 2 | 0 | 0 | 0 | 0 | 0 |
| 2015 | 0 | 2 | 4 | 1 | 1 | 0 | 0 | 2 | 0 | 0 | 0 | 0 | 1 |
| 2015 | 1 | 2 | 0 | 1 | 0 | 4 | 0 | 2 | 0 | 0 | 0 | 0 | 0 |
| 2015 | 0 | 0 | 3 | 1 | 0 | 2 | 0 | 0 | 0 | 0 | 0 | 0 | 0 |
| 2015 | 1 | 1 | 2 | 1 | 0 | 1 | 0 | 0 | 0 | 1 | 0 | 0 | 0 |
| 2015 | 0 | 1 | 1 | 1 | 2 | 3 | 0 | 0 | 0 | 0 | 0 | 0 | 0 |
| 2015 | 0 | 2 | 3 | 1 | 0 | 0 | 0 | 0 | 0 | 0 | 0 | 0 | 0 |
| 2015 | 0 | 2 | 2 | 1 | 0 | 1 | 0 | 1 | 0 | 1 | 0 | 0 | 0 |
| 2015 | 1 | 2 | 2 | 1 | 1 | 1 | 0 | 0 | 0 | 1 | 0 | 0 | 0 |
| 2015 | 0 | 0 | 2 | 1 | 0 | 1 | 0 | 0 | 0 | 1 | 0 | 0 | 1 |
| 2015 | 0 | 4 | 1 | 1 | 2 | 2 | 0 | 0 | 0 | 1 | 0 | 0 | 0 |
| 2015 | 0 | 1 | 4 | 1 | 0 | 0 | 0 | 2 | 0 | 1 | 0 | 0 | 0 |
| 2015 | 0 | 1 | 1 | 1 | 2 | 0 | 0 | 0 | 0 | 1 | 0 | 0 | 1 |
| 2015 | 0 | 2 | 0 | 1 | 1 | 1 | 0 | 0 | 0 | 1 | 0 | 0 | 0 |
| 2015 | 1 | 1 | 0 | 1 | 0 | 4 | 0 | 2 | 0 | 0 | 0 | 0 | 0 |
| 2015 | 0 | 2 | 1 | 1 | 0 | 4 | 0 | 0 | 0 | 0 | 0 | 0 | 0 |
| 2015 | 1 | 1 | 1 | 1 | 0 | 4 | 0 | 0 | 0 | 0 | 0 | 0 | 0 |
| 2015 | 0 | 2 | 1 | 1 | 0 | 3 | 0 | 0 | 0 | 0 | 0 | 0 | 0 |
| 2015 | 0 | 2 | 1 | 1 | 1 | 4 | 0 | 2 | 0 | 1 | 0 | 0 | 0 |
| 2015 | 0 | 3 | 1 | 1 | 0 | 3 | 0 | 0 | 0 | 1 | 0 | 0 | 0 |
| 2015 | 1 | 0 | 2 | 1 | 0 | 1 | 0 | 1 | 0 | 1 | 0 | 0 | 0 |
| 2015 | 1 | 1 | 2 | 1 | 0 | 1 | 0 | 2 | 0 | 1 | 0 | 0 | 0 |
| 2015 | 0 | 4 | 4 | 2 | 0 | 4 | 0 | 2 | 0 | 1 | 2 | 2 | 2 |
| 2015 | 0 | 1 | 1 | 1 | 1 | 1 | 0 | 0 | 0 | 1 | 0 | 0 | 0 |
| 2015 | 1 | 3 | 1 | 0 | 1 | 2 | 0 | 0 | 0 | 1 | 0 | 0 | 0 |
| 2015 | 0 | 3 | 1 | 1 | 0 | 2 | 0 | 2 | 0 | 1 | 0 | 0 | 0 |
| 2015 | 0 | 1 | 3 | 1 | 0 | 2 | 0 | 0 | 0 | 1 | 0 | 0 | 0 |
| 2015 | 1 | 1 | 1 | 1 | 1 | 0 | 0 | 0 | 0 | 1 | 1 | 0 | 0 |
| 2015 | 0 | 1 | 1 | 1 | 0 | 3 | 0 | 0 | 0 | 0 | 0 | 0 | 0 |
| 2015 | 0 | 2 | 3 | 1 | 0 | 1 | 0 | 0 | 0 | 1 | 0 | 0 | 0 |
| 2015 | 0 | 2 | 0 | 1 | 1 | 0 | 0 | 0 | 0 | 0 | 0 | 0 | 0 |
| 2015 | 1 | 1 | 2 | 1 | 0 | 2 | 0 | 2 | 0 | 1 | 0 | 1 | 0 |
| 2015 | 0 | 1 | 2 | 1 | 0 | 1 | 0 | 0 | 0 | 1 | 0 | 0 | 0 |
| 2015 | 0 | 1 | 0 | 1 | 1 | 0 | 0 | 0 | 0 | 1 | 0 | 0 | 0 |
| 2015 | 0 | 0 | 1 | 1 | 0 | 3 | 0 | 0 | 0 | 0 | 0 | 0 | 0 |
| 2015 | 1 | 1 | 1 | 1 | 0 | 2 | 0 | 0 | 0 | 1 | 0 | 0 | 0 |
| 2015 | 0 | 4 | 0 | 1 | 1 | 3 | 0 | 0 | 0 | 0 | 0 | 0 | 1 |
| 2015 | 1 | 1 | 1 | 1 | 0 | 2 | 0 | 0 | 0 | 1 | 0 | 0 | 0 |
| 2015 | 0 | 0 | 0 | 1 | 0 | 0 | 0 | 0 | 0 | 1 | 0 | 0 | 1 |
| 2015 | 1 | 3 | 3 | 1 | 1 | 2 | 0 | 0 | 0 | 1 | 0 | 0 | 0 |
| 2015 | 1 | 1 | 0 | 1 | 0 | 2 | 0 | 2 | 0 | 1 | 0 | 0 | 0 |
| 2015 | 1 | 1 | 0 | 1 | 2 | 3 | 0 | 2 | 0 | 0 | 2 | 2 | 2 |
| 2015 | 0 | 0 | 4 | 0 | 0 | 1 | 0 | 2 | 0 | 2 | 0 | 0 | 0 |
| 2015 | 1 | 1 | 2 | 1 | 0 | 0 | 0 | 0 | 0 | 1 | 0 | 0 | 0 |
| 2015 | 0 | 1 | 0 | 1 | 0 | 3 | 0 | 2 | 0 | 0 | 1 | 0 | 0 |
| 2015 | 0 | 3 | 3 | 1 | 0 | 1 | 0 | 2 | 0 | 1 | 0 | 0 | 0 |
| 2015 | 0 | 1 | 4 | 1 | 2 | 3 | 0 | 2 | 0 | 1 | 0 | 0 | 0 |
| 2015 | 0 | 2 | 4 | 1 | 0 | 1 | 0 | 2 | 0 | 2 | 0 | 0 | 0 |
| 2015 | 1 | 3 | 1 | 1 | 0 | 1 | 0 | 0 | 0 | 1 | 0 | 0 | 0 |
| 2015 | 1 | 0 | 4 | 1 | 2 | 4 | 0 | 2 | 0 | 0 | 0 | 0 | 0 |
| 2015 | 1 | 3 | 1 | 0 | 0 | 0 | 0 | 0 | 0 | 0 | 0 | 0 | 0 |
| 2015 | 0 | 1 | 0 | 0 | 0 | 2 | 1 | 0 | 0 | 0 | 0 | 0 | 0 |
| 2015 | 1 | 0 | 0 | 1 | 1 | 2 | 1 | 1 | 0 | 0 | 0 | 1 | 0 |
| 2015 | 0 | 1 | 1 | 0 | 0 | 2 | 1 | 1 | 0 | 1 | 0 | 0 | 0 |
| 2015 | 1 | 0 | 2 | 1 | 0 | 3 | 1 | 2 | 0 | 1 | 0 | 1 | 0 |
| 2015 | 0 | 4 | 4 | 2 | 0 | 4 | 1 | 2 | 0 | 0 | 0 | 0 | 0 |
| 2015 | 1 | 1 | 1 | 1 | 2 | 0 | 1 | 1 | 0 | 0 | 0 | 0 | 0 |
| 2015 | 0 | 1 | 4 | 2 | 1 | 1 | 1 | 0 | 0 | 0 | 0 | 0 | 0 |
| 2015 | 1 | 3 | 4 | 1 | 0 | 4 | 1 | 0 | 0 | 0 | 0 | 0 | 0 |
| 2015 | 1 | 1 | 2 | 1 | 0 | 3 | 1 | 0 | 0 | 1 | 0 | 0 | 0 |
| 2015 | 1 | 3 | 1 | 1 | 0 | 4 | 1 | 2 | 0 | 0 | 0 | 0 | 0 |
| 2015 | 1 | 3 | 1 | 1 | 0 | 4 | 1 | 0 | 0 | 0 | 0 | 0 | 0 |
| 2015 | 1 | 3 | 2 | 0 | 2 | 2 | 1 | 0 | 0 | 0 | 0 | 0 | 0 |
| 2015 | 1 | 2 | 2 | 0 | 0 | 3 | 1 | 1 | 0 | 0 | 0 | 0 | 0 |
| 2015 | 1 | 1 | 0 | 1 | 1 | 4 | 0 | 2 | 0 | 0 | 0 | 0 | 0 |
| 2015 | 0 | 0 | 4 | 1 | 0 | 2 | 0 | 0 | 0 | 1 | 0 | 0 | 1 |
| 2015 | 0 | 1 | 1 | 1 | 0 | 1 | 0 | 0 | 0 | 1 | 0 | 0 | 0 |
| 2015 | 0 | 1 | 4 | 1 | 0 | 1 | 0 | 0 | 0 | 0 | 0 | 0 | 0 |
| 2015 | 0 | 1 | 1 | 1 | 2 | 3 | 0 | 1 | 0 | 0 | 0 | 0 | 0 |
| 2015 | 0 | 1 | 0 | 1 | 1 | 0 | 0 | 1 | 0 | 0 | 0 | 0 | 0 |
| 2015 | 1 | 3 | 3 | 0 | 0 | 2 | 0 | 0 | 0 | 0 | 0 | 0 | 0 |
| 2015 | 0 | 3 | 2 | 1 | 0 | 3 | 0 | 0 | 0 | 0 | 0 | 0 | 0 |
| 2015 | 0 | 1 | 4 | 1 | 2 | 3 | 0 | 0 | 0 | 0 | 2 | 2 | 2 |
| 2015 | 0 | 1 | 4 | 1 | 1 | 3 | 0 | 0 | 0 | 1 | 0 | 0 | 0 |
| 2015 | 1 | 1 | 4 | 2 | 0 | 2 | 0 | 0 | 0 | 0 | 0 | 0 | 0 |
| 2015 | 1 | 1 | 3 | 1 | 0 | 2 | 0 | 0 | 0 | 1 | 0 | 0 | 0 |
| 2015 | 1 | 1 | 4 | 2 | 0 | 2 | 0 | 0 | 0 | 0 | 0 | 0 | 0 |
| 2015 | 0 | 0 | 4 | 2 | 0 | 1 | 0 | 2 | 0 | 0 | 0 | 0 | 0 |
| 2015 | 0 | 2 | 0 | 1 | 1 | 2 | 0 | 0 | 0 | 0 | 0 | 0 | 0 |
| 2015 | 1 | 0 | 0 | 1 | 2 | 3 | 0 | 0 | 0 | 0 | 0 | 0 | 0 |
| 2015 | 0 | 3 | 4 | 1 | 0 | 3 | 0 | 0 | 0 | 0 | 0 | 0 | 0 |
| 2015 | 0 | 2 | 0 | 1 | 0 | 4 | 0 | 0 | 0 | 0 | 2 | 2 | 2 |
| 2015 | 1 | 0 | 1 | 1 | 0 | 4 | 0 | 0 | 0 | 0 | 0 | 0 | 0 |
| 2015 | 1 | 3 | 0 | 0 | 1 | 3 | 0 | 2 | 0 | 0 | 0 | 0 | 0 |
| 2015 | 0 | 1 | 0 | 1 | 1 | 2 | 0 | 0 | 0 | 1 | 0 | 0 | 1 |
| 2015 | 1 | 1 | 1 | 1 | 0 | 4 | 0 | 0 | 0 | 0 | 0 | 0 | 0 |
| 2015 | 1 | 1 | 2 | 2 | 0 | 1 | 0 | 0 | 0 | 1 | 0 | 0 | 0 |
| 2015 | 1 | 4 | 4 | 2 | 0 | 1 | 0 | 0 | 0 | 1 | 2 | 2 | 2 |
| 2015 | 0 | 0 | 0 | 1 | 2 | 0 | 0 | 1 | 0 | 0 | 0 | 0 | 0 |
| 2015 | 1 | 0 | 1 | 1 | 0 | 1 | 0 | 0 | 0 | 1 | 0 | 0 | 0 |
| 2015 | 0 | 0 | 0 | 1 | 0 | 2 | 0 | 2 | 0 | 0 | 0 | 0 | 0 |
| 2015 | 0 | 0 | 0 | 1 | 0 | 1 | 0 | 0 | 0 | 1 | 0 | 0 | 0 |
| 2015 | 0 | 0 | 0 | 1 | 1 | 0 | 0 | 0 | 0 | 0 | 0 | 0 | 0 |
| 2015 | 1 | 3 | 2 | 1 | 0 | 1 | 0 | 2 | 0 | 1 | 0 | 0 | 0 |
| 2015 | 0 | 4 | 4 | 1 | 2 | 1 | 0 | 0 | 0 | 2 | 1 | 0 | 0 |
| 2015 | 0 | 0 | 0 | 1 | 1 | 2 | 0 | 0 | 0 | 2 | 0 | 0 | 0 |
| 2015 | 0 | 0 | 1 | 1 | 2 | 3 | 0 | 2 | 0 | 0 | 2 | 2 | 2 |
| 2015 | 1 | 4 | 4 | 1 | 0 | 1 | 0 | 2 | 0 | 2 | 0 | 0 | 0 |
| 2015 | 0 | 2 | 1 | 2 | 1 | 2 | 0 | 0 | 0 | 1 | 0 | 0 | 0 |
| 2015 | 0 | 3 | 1 | 1 | 1 | 2 | 0 | 0 | 0 | 1 | 0 | 0 | 0 |
| 2015 | 0 | 4 | 4 | 1 | 0 | 1 | 0 | 0 | 0 | 1 | 2 | 2 | 2 |
| 2015 | 0 | 0 | 0 | 1 | 1 | 3 | 0 | 0 | 0 | 1 | 0 | 0 | 0 |
| 2015 | 0 | 0 | 1 | 1 | 0 | 4 | 0 | 0 | 0 | 0 | 0 | 0 | 0 |
| 2015 | 0 | 1 | 1 | 1 | 2 | 3 | 0 | 1 | 0 | 2 | 0 | 0 | 0 |
| 2015 | 1 | 4 | 1 | 1 | 1 | 4 | 0 | 2 | 0 | 0 | 0 | 0 | 0 |
| 2015 | 0 | 1 | 1 | 2 | 1 | 0 | 0 | 0 | 0 | 0 | 0 | 0 | 0 |
| 2015 | 0 | 2 | 1 | 1 | 0 | 1 | 0 | 0 | 0 | 1 | 0 | 0 | 0 |
| 2015 | 0 | 1 | 4 | 1 | 0 | 2 | 0 | 0 | 0 | 0 | 0 | 0 | 0 |
| 2015 | 2 | 4 | 4 | 1 | 2 | 3 | 0 | 0 | 0 | 0 | 1 | 0 | 0 |
| 2015 | 0 | 0 | 1 | 1 | 1 | 2 | 0 | 0 | 0 | 1 | 0 | 0 | 0 |
| 2015 | 0 | 1 | 1 | 0 | 0 | 0 | 0 | 0 | 0 | 1 | 0 | 0 | 0 |
| 2015 | 0 | 1 | 0 | 1 | 1 | 3 | 0 | 0 | 0 | 0 | 0 | 0 | 0 |
| 2015 | 0 | 1 | 2 | 0 | 0 | 2 | 0 | 0 | 0 | 1 | 0 | 0 | 0 |
| 2015 | 0 | 2 | 4 | 1 | 0 | 2 | 0 | 0 | 0 | 1 | 0 | 0 | 0 |
| 2015 | 1 | 3 | 1 | 1 | 1 | 3 | 0 | 0 | 0 | 0 | 0 | 0 | 0 |
| 2015 | 1 | 0 | 1 | 1 | 0 | 1 | 0 | 2 | 0 | 1 | 0 | 0 | 0 |
| 2015 | 1 | 3 | 3 | 1 | 0 | 2 | 0 | 0 | 0 | 1 | 0 | 0 | 0 |
| 2015 | 1 | 1 | 2 | 1 | 0 | 2 | 0 | 0 | 0 | 1 | 0 | 0 | 0 |
| 2015 | 1 | 1 | 4 | 1 | 1 | 0 | 0 | 0 | 0 | 0 | 0 | 0 | 0 |
| 2015 | 1 | 3 | 1 | 1 | 1 | 0 | 0 | 0 | 0 | 1 | 0 | 0 | 0 |
| 2015 | 0 | 0 | 1 | 1 | 0 | 4 | 0 | 2 | 0 | 0 | 0 | 0 | 0 |
| 2015 | 0 | 4 | 0 | 1 | 0 | 1 | 0 | 2 | 0 | 0 | 0 | 0 | 0 |
| 2015 | 0 | 2 | 2 | 1 | 0 | 2 | 0 | 0 | 0 | 1 | 0 | 0 | 1 |
| 2015 | 0 | 2 | 4 | 1 | 1 | 0 | 0 | 1 | 0 | 1 | 0 | 0 | 0 |
| 2015 | 0 | 1 | 0 | 1 | 0 | 3 | 0 | 2 | 0 | 0 | 0 | 0 | 0 |
| 2015 | 0 | 1 | 1 | 1 | 2 | 3 | 0 | 0 | 2 | 0 | 0 | 0 | 0 |
| 2015 | 0 | 2 | 4 | 1 | 0 | 3 | 0 | 0 | 0 | 0 | 0 | 0 | 0 |
| 2015 | 0 | 3 | 2 | 1 | 1 | 0 | 0 | 0 | 0 | 1 | 0 | 0 | 0 |
| 2015 | 0 | 0 | 1 | 1 | 0 | 1 | 0 | 0 | 0 | 0 | 0 | 0 | 1 |
| 2015 | 1 | 1 | 1 | 0 | 0 | 2 | 0 | 0 | 0 | 1 | 0 | 0 | 0 |
| 2015 | 0 | 2 | 3 | 1 | 0 | 0 | 0 | 2 | 0 | 1 | 0 | 0 | 0 |
| 2015 | 1 | 2 | 1 | 1 | 0 | 1 | 0 | 2 | 0 | 1 | 0 | 0 | 0 |
| 2015 | 1 | 0 | 1 | 1 | 0 | 2 | 0 | 0 | 0 | 1 | 0 | 0 | 0 |
| 2015 | 0 | 1 | 2 | 1 | 0 | 1 | 0 | 2 | 0 | 0 | 0 | 0 | 0 |
| 2015 | 0 | 0 | 3 | 1 | 0 | 1 | 0 | 0 | 0 | 1 | 0 | 0 | 0 |
| 2015 | 1 | 1 | 4 | 1 | 2 | 0 | 0 | 0 | 0 | 0 | 0 | 0 | 0 |
| 2015 | 0 | 0 | 4 | 0 | 0 | 1 | 0 | 0 | 0 | 0 | 0 | 0 | 0 |
| 2015 | 0 | 3 | 4 | 0 | 0 | 2 | 0 | 0 | 0 | 0 | 0 | 0 | 0 |
| 2015 | 0 | 1 | 0 | 0 | 0 | 2 | 0 | 2 | 0 | 0 | 0 | 0 | 0 |
| 2015 | 0 | 3 | 4 | 1 | 0 | 1 | 0 | 0 | 0 | 0 | 0 | 0 | 0 |
| 2015 | 0 | 2 | 0 | 1 | 1 | 0 | 0 | 0 | 1 | 0 | 0 | 0 | 0 |
| 2015 | 0 | 1 | 1 | 1 | 1 | 2 | 0 | 0 | 0 | 1 | 0 | 0 | 0 |
| 2015 | 0 | 3 | 1 | 1 | 1 | 0 | 0 | 0 | 0 | 0 | 0 | 0 | 0 |
| 2015 | 1 | 3 | 4 | 2 | 1 | 0 | 0 | 0 | 0 | 1 | 0 | 0 | 0 |
| 2015 | 1 | 3 | 0 | 1 | 1 | 0 | 0 | 2 | 0 | 0 | 0 | 0 | 0 |
| 2015 | 0 | 1 | 4 | 1 | 1 | 3 | 0 | 2 | 0 | 0 | 0 | 0 | 0 |
| 2015 | 0 | 0 | 4 | 1 | 0 | 1 | 0 | 0 | 0 | 0 | 0 | 0 | 0 |
| 2015 | 0 | 2 | 0 | 1 | 0 | 2 | 0 | 2 | 0 | 1 | 0 | 0 | 0 |
| 2015 | 1 | 3 | 1 | 1 | 0 | 4 | 0 | 0 | 0 | 0 | 0 | 0 | 0 |
| 2015 | 1 | 1 | 1 | 1 | 0 | 2 | 0 | 0 | 0 | 1 | 0 | 0 | 0 |
| 2015 | 0 | 1 | 4 | 1 | 0 | 0 | 0 | 1 | 0 | 0 | 0 | 0 | 0 |
| 2015 | 0 | 3 | 1 | 1 | 0 | 3 | 0 | 0 | 0 | 0 | 0 | 0 | 0 |
| 2015 | 1 | 2 | 2 | 0 | 0 | 1 | 0 | 0 | 0 | 1 | 0 | 0 | 0 |
| 2015 | 1 | 1 | 0 | 1 | 0 | 2 | 0 | 0 | 0 | 0 | 0 | 0 | 0 |
| 2015 | 0 | 0 | 0 | 1 | 0 | 1 | 0 | 0 | 0 | 0 | 0 | 0 | 0 |
| 2015 | 0 | 0 | 4 | 1 | 0 | 2 | 0 | 0 | 0 | 1 | 0 | 0 | 0 |
| 2015 | 0 | 3 | 4 | 1 | 1 | 1 | 0 | 0 | 0 | 1 | 0 | 0 | 0 |
| 2015 | 1 | 1 | 2 | 1 | 0 | 2 | 0 | 2 | 0 | 1 | 0 | 0 | 0 |
| 2015 | 0 | 2 | 4 | 1 | 2 | 0 | 0 | 2 | 0 | 2 | 0 | 0 | 0 |
| 2015 | 1 | 2 | 4 | 1 | 1 | 4 | 0 | 0 | 0 | 0 | 0 | 0 | 0 |
| 2015 | 0 | 3 | 0 | 1 | 0 | 3 | 0 | 1 | 0 | 0 | 0 | 0 | 0 |
| 2015 | 0 | 2 | 4 | 1 | 0 | 2 | 0 | 0 | 0 | 0 | 0 | 0 | 0 |
| 2015 | 1 | 3 | 0 | 1 | 0 | 3 | 0 | 0 | 0 | 0 | 0 | 0 | 0 |
| 2015 | 0 | 1 | 1 | 1 | 0 | 1 | 0 | 0 | 0 | 1 | 0 | 0 | 0 |
| 2015 | 0 | 3 | 1 | 0 | 0 | 2 | 0 | 2 | 0 | 0 | 0 | 0 | 0 |
| 2015 | 0 | 3 | 2 | 1 | 0 | 1 | 0 | 0 | 0 | 1 | 0 | 0 | 0 |
| 2015 | 0 | 3 | 1 | 1 | 0 | 3 | 0 | 0 | 0 | 0 | 0 | 0 | 0 |
| 2015 | 0 | 1 | 0 | 1 | 0 | 3 | 0 | 0 | 0 | 0 | 0 | 0 | 0 |
| 2015 | 1 | 3 | 4 | 0 | 1 | 0 | 0 | 0 | 1 | 1 | 0 | 0 | 1 |
| 2015 | 0 | 1 | 0 | 1 | 0 | 2 | 0 | 2 | 0 | 1 | 0 | 0 | 0 |
| 2015 | 1 | 3 | 0 | 1 | 1 | 0 | 0 | 0 | 0 | 0 | 0 | 0 | 0 |
| 2015 | 0 | 1 | 4 | 0 | 0 | 0 | 0 | 2 | 0 | 0 | 0 | 0 | 0 |
| 2015 | 0 | 2 | 4 | 1 | 2 | 4 | 0 | 0 | 0 | 2 | 0 | 0 | 0 |
| 2015 | 1 | 3 | 3 | 1 | 2 | 4 | 0 | 2 | 0 | 2 | 0 | 1 | 0 |
| 2015 | 1 | 0 | 3 | 1 | 0 | 1 | 0 | 0 | 0 | 1 | 0 | 0 | 0 |
| 2015 | 1 | 2 | 2 | 0 | 0 | 2 | 0 | 0 | 0 | 1 | 0 | 0 | 0 |
| 2015 | 1 | 0 | 4 | 1 | 1 | 3 | 0 | 0 | 0 | 0 | 0 | 0 | 0 |
| 2015 | 0 | 1 | 4 | 1 | 0 | 2 | 0 | 0 | 0 | 0 | 0 | 0 | 0 |
| 2015 | 1 | 2 | 0 | 1 | 1 | 4 | 0 | 0 | 0 | 0 | 0 | 0 | 0 |
| 2015 | 1 | 1 | 4 | 1 | 0 | 0 | 0 | 0 | 0 | 1 | 0 | 0 | 0 |
| 2015 | 1 | 3 | 4 | 1 | 0 | 1 | 0 | 0 | 0 | 1 | 0 | 0 | 0 |
| 2015 | 0 | 1 | 0 | 0 | 0 | 3 | 0 | 0 | 0 | 2 | 0 | 0 | 0 |
| 2015 | 1 | 2 | 0 | 1 | 0 | 2 | 0 | 0 | 0 | 0 | 0 | 0 | 0 |
| 2015 | 1 | 3 | 1 | 1 | 0 | 4 | 0 | 0 | 0 | 0 | 0 | 0 | 0 |
| 2015 | 0 | 1 | 4 | 1 | 1 | 3 | 0 | 0 | 0 | 0 | 0 | 0 | 0 |
| 2015 | 1 | 3 | 4 | 1 | 0 | 1 | 0 | 2 | 0 | 1 | 0 | 0 | 0 |
| 2015 | 1 | 0 | 4 | 1 | 0 | 3 | 0 | 0 | 0 | 0 | 0 | 0 | 0 |
| 2015 | 0 | 1 | 4 | 1 | 0 | 1 | 0 | 0 | 0 | 0 | 0 | 0 | 0 |
| 2015 | 1 | 3 | 0 | 1 | 1 | 2 | 0 | 0 | 0 | 0 | 0 | 0 | 1 |
| 2015 | 0 | 0 | 4 | 1 | 2 | 3 | 0 | 0 | 0 | 0 | 0 | 0 | 1 |
| 2015 | 1 | 4 | 4 | 1 | 2 | 3 | 0 | 0 | 0 | 0 | 2 | 2 | 2 |
| 2015 | 1 | 3 | 2 | 1 | 0 | 3 | 0 | 2 | 0 | 1 | 0 | 0 | 0 |
| 2015 | 1 | 0 | 1 | 1 | 2 | 3 | 0 | 0 | 0 | 0 | 1 | 0 | 0 |
| 2015 | 0 | 3 | 4 | 0 | 1 | 0 | 0 | 0 | 0 | 0 | 0 | 0 | 0 |
| 2015 | 0 | 0 | 4 | 1 | 1 | 0 | 0 | 0 | 0 | 0 | 0 | 0 | 0 |
| 2015 | 0 | 3 | 3 | 1 | 0 | 0 | 0 | 0 | 0 | 0 | 0 | 0 | 0 |
| 2015 | 1 | 3 | 0 | 1 | 0 | 4 | 0 | 0 | 0 | 0 | 0 | 0 | 0 |
| 2015 | 0 | 1 | 0 | 1 | 1 | 4 | 0 | 0 | 0 | 0 | 0 | 0 | 0 |
| 2015 | 0 | 0 | 0 | 1 | 0 | 4 | 0 | 0 | 0 | 0 | 0 | 0 | 0 |
| 2015 | 1 | 3 | 2 | 1 | 0 | 4 | 0 | 0 | 0 | 0 | 0 | 0 | 0 |
| 2015 | 0 | 3 | 1 | 1 | 1 | 3 | 0 | 0 | 0 | 0 | 0 | 0 | 0 |
| 2015 | 1 | 3 | 4 | 1 | 0 | 3 | 0 | 2 | 0 | 0 | 0 | 0 | 1 |
| 2015 | 0 | 1 | 0 | 1 | 1 | 2 | 0 | 0 | 0 | 1 | 0 | 0 | 0 |
| 2015 | 1 | 1 | 1 | 1 | 1 | 0 | 0 | 0 | 0 | 0 | 0 | 0 | 0 |
| 2015 | 1 | 1 | 1 | 2 | 1 | 0 | 0 | 0 | 1 | 0 | 0 | 0 | 0 |
| 2015 | 0 | 2 | 2 | 1 | 1 | 1 | 0 | 2 | 0 | 1 | 0 | 0 | 0 |
| 2015 | 1 | 0 | 1 | 1 | 0 | 2 | 0 | 0 | 0 | 1 | 0 | 0 | 0 |
| 2015 | 0 | 0 | 0 | 1 | 0 | 3 | 0 | 0 | 0 | 0 | 0 | 0 | 0 |
| 2015 | 1 | 3 | 2 | 1 | 1 | 0 | 0 | 2 | 1 | 1 | 0 | 0 | 0 |
| 2015 | 1 | 3 | 2 | 1 | 1 | 0 | 0 | 2 | 1 | 1 | 0 | 0 | 0 |
| 2015 | 1 | 3 | 2 | 1 | 1 | 0 | 0 | 2 | 1 | 1 | 0 | 0 | 0 |
| 2015 | 0 | 3 | 0 | 0 | 1 | 1 | 0 | 0 | 0 | 0 | 0 | 0 | 0 |
| 2015 | 1 | 2 | 4 | 1 | 2 | 0 | 0 | 1 | 1 | 2 | 0 | 0 | 0 |
| 2015 | 0 | 1 | 0 | 0 | 1 | 0 | 0 | 0 | 0 | 0 | 0 | 0 | 0 |
| 2015 | 0 | 1 | 4 | 0 | 1 | 2 | 0 | 0 | 0 | 1 | 0 | 0 | 0 |
| 2015 | 0 | 2 | 4 | 1 | 0 | 0 | 0 | 0 | 0 | 1 | 0 | 0 | 0 |
| 2015 | 0 | 2 | 4 | 1 | 2 | 1 | 0 | 0 | 0 | 1 | 0 | 0 | 0 |
| 2015 | 0 | 3 | 4 | 1 | 2 | 1 | 0 | 0 | 0 | 2 | 0 | 0 | 0 |
| 2015 | 0 | 4 | 4 | 2 | 0 | 0 | 0 | 0 | 0 | 2 | 2 | 2 | 2 |
| 2015 | 0 | 2 | 1 | 1 | 1 | 2 | 0 | 2 | 0 | 1 | 0 | 0 | 1 |
| 2015 | 1 | 3 | 0 | 0 | 1 | 2 | 0 | 0 | 0 | 0 | 0 | 1 | 0 |
| 2015 | 0 | 0 | 4 | 2 | 0 | 2 | 1 | 2 | 0 | 0 | 2 | 2 | 2 |
| 2015 | 1 | 3 | 1 | 1 | 0 | 1 | 1 | 0 | 0 | 1 | 1 | 0 | 0 |
| 2015 | 1 | 1 | 1 | 1 | 0 | 2 | 1 | 0 | 0 | 1 | 0 | 0 | 0 |
| 2015 | 1 | 2 | 0 | 1 | 0 | 4 | 1 | 0 | 0 | 0 | 0 | 0 | 0 |
| 2015 | 0 | 0 | 0 | 1 | 1 | 4 | 0 | 0 | 0 | 0 | 0 | 0 | 0 |
| 2015 | 0 | 0 | 1 | 1 | 0 | 4 | 0 | 0 | 0 | 2 | 0 | 0 | 0 |
| 2015 | 0 | 3 | 0 | 0 | 0 | 2 | 0 | 0 | 0 | 0 | 0 | 0 | 0 |
| 2015 | 1 | 3 | 3 | 1 | 1 | 0 | 0 | 2 | 1 | 0 | 2 | 2 | 2 |
| 2015 | 0 | 4 | 4 | 2 | 2 | 3 | 0 | 0 | 0 | 0 | 2 | 2 | 2 |
| 2015 | 0 | 0 | 0 | 0 | 0 | 3 | 0 | 0 | 0 | 0 | 0 | 0 | 0 |
| 2015 | 0 | 2 | 1 | 1 | 0 | 2 | 0 | 0 | 0 | 0 | 0 | 0 | 0 |
| 2015 | 0 | 3 | 1 | 0 | 0 | 4 | 0 | 0 | 0 | 0 | 0 | 0 | 0 |
| 2015 | 0 | 4 | 4 | 2 | 2 | 2 | 0 | 0 | 0 | 2 | 2 | 2 | 2 |
| 2015 | 1 | 3 | 4 | 2 | 0 | 4 | 0 | 0 | 0 | 0 | 2 | 2 | 2 |
| 2015 | 0 | 3 | 3 | 1 | 0 | 0 | 0 | 0 | 0 | 0 | 0 | 0 | 0 |
| 2015 | 0 | 1 | 2 | 1 | 0 | 2 | 0 | 0 | 0 | 1 | 0 | 0 | 0 |
| 2015 | 0 | 1 | 1 | 1 | 0 | 2 | 0 | 2 | 0 | 1 | 0 | 0 | 0 |
| 2015 | 1 | 3 | 3 | 1 | 2 | 4 | 0 | 0 | 0 | 1 | 0 | 0 | 0 |
| 2015 | 0 | 0 | 1 | 1 | 2 | 3 | 0 | 0 | 0 | 0 | 2 | 2 | 2 |
| 2015 | 0 | 3 | 2 | 1 | 0 | 1 | 0 | 0 | 0 | 1 | 0 | 0 | 0 |
| 2015 | 1 | 3 | 3 | 0 | 0 | 1 | 0 | 0 | 0 | 1 | 0 | 0 | 0 |
| 2015 | 1 | 2 | 0 | 1 | 2 | 4 | 0 | 0 | 0 | 0 | 2 | 2 | 2 |
| 2015 | 0 | 3 | 3 | 1 | 0 | 1 | 0 | 2 | 0 | 1 | 0 | 0 | 0 |
| 2015 | 0 | 3 | 1 | 0 | 0 | 1 | 0 | 0 | 0 | 0 | 0 | 0 | 0 |
| 2015 | 0 | 3 | 1 | 0 | 1 | 1 | 0 | 0 | 0 | 1 | 0 | 0 | 0 |
| 2015 | 0 | 4 | 4 | 1 | 1 | 0 | 0 | 0 | 2 | 2 | 2 | 2 | 2 |
| 2015 | 0 | 1 | 4 | 0 | 0 | 0 | 0 | 0 | 0 | 0 | 0 | 0 | 0 |
| 2015 | 1 | 3 | 1 | 1 | 0 | 0 | 0 | 0 | 0 | 1 | 0 | 0 | 0 |
| 2015 | 1 | 3 | 4 | 1 | 1 | 0 | 0 | 0 | 0 | 0 | 0 | 0 | 0 |
| 2016 | 0 | 3 | 2 | 1 | 1 | 3 | 0 | 2 | 0 | 0 | 0 | 0 | 0 |
| 2016 | 1 | 2 | 0 | 1 | 1 | 2 | 1 | 2 | 0 | 0 | 0 | 0 | 0 |
| 2016 | 1 | 1 | 1 | 2 | 0 | 3 | 0 | 2 | 0 | 1 | 0 | 0 | 0 |
| 2016 | 0 | 0 | 1 | 0 | 0 | 2 | 0 | 2 | 0 | 0 | 0 | 0 | 0 |
| 2016 | 1 | 1 | 4 | 1 | 0 | 4 | 0 | 2 | 0 | 0 | 0 | 1 | 0 |
| 2016 | 0 | 0 | 4 | 1 | 0 | 3 | 0 | 2 | 0 | 0 | 0 | 0 | 0 |
| 2016 | 0 | 1 | 1 | 1 | 0 | 2 | 0 | 2 | 0 | 1 | 0 | 0 | 0 |
| 2016 | 0 | 2 | 0 | 1 | 1 | 3 | 0 | 2 | 0 | 1 | 0 | 0 | 0 |
| 2016 | 0 | 2 | 1 | 1 | 1 | 0 | 0 | 2 | 0 | 0 | 0 | 0 | 0 |
| 2016 | 1 | 3 | 4 | 1 | 0 | 2 | 0 | 2 | 0 | 1 | 0 | 0 | 0 |
| 2016 | 1 | 0 | 0 | 1 | 0 | 4 | 0 | 2 | 0 | 0 | 0 | 0 | 0 |
| 2016 | 0 | 1 | 0 | 1 | 0 | 0 | 0 | 2 | 0 | 1 | 0 | 0 | 0 |
| 2016 | 1 | 0 | 4 | 1 | 0 | 2 | 0 | 2 | 0 | 0 | 0 | 0 | 0 |
| 2016 | 0 | 1 | 1 | 1 | 0 | 2 | 1 | 2 | 0 | 1 | 0 | 0 | 0 |
| 2016 | 1 | 1 | 1 | 1 | 0 | 2 | 0 | 2 | 0 | 1 | 0 | 0 | 0 |
| 2016 | 0 | 1 | 3 | 1 | 0 | 2 | 0 | 2 | 0 | 0 | 0 | 0 | 0 |
| 2016 | 1 | 2 | 1 | 1 | 0 | 4 | 0 | 2 | 0 | 0 | 0 | 0 | 0 |
| 2016 | 1 | 0 | 1 | 1 | 1 | 3 | 0 | 2 | 0 | 0 | 0 | 0 | 0 |
| 2016 | 1 | 2 | 2 | 1 | 1 | 0 | 0 | 2 | 0 | 1 | 0 | 0 | 0 |
| 2016 | 1 | 1 | 0 | 1 | 1 | 4 | 0 | 2 | 0 | 0 | 0 | 0 | 0 |
| 2016 | 1 | 2 | 1 | 1 | 0 | 3 | 0 | 2 | 0 | 0 | 0 | 0 | 0 |
| 2016 | 0 | 0 | 1 | 1 | 1 | 3 | 0 | 2 | 0 | 1 | 0 | 0 | 0 |
| 2016 | 1 | 3 | 0 | 1 | 1 | 3 | 0 | 2 | 0 | 0 | 0 | 0 | 0 |
| 2016 | 1 | 1 | 1 | 1 | 0 | 2 | 0 | 2 | 0 | 1 | 0 | 0 | 0 |
| 2016 | 0 | 3 | 1 | 1 | 0 | 2 | 0 | 2 | 0 | 1 | 0 | 0 | 0 |
| 2016 | 1 | 1 | 2 | 1 | 0 | 1 | 0 | 2 | 0 | 1 | 0 | 0 | 0 |
| 2016 | 1 | 3 | 2 | 1 | 0 | 0 | 0 | 2 | 0 | 1 | 0 | 0 | 0 |
| 2016 | 0 | 3 | 1 | 1 | 1 | 0 | 0 | 2 | 0 | 0 | 0 | 0 | 0 |
| 2016 | 1 | 1 | 4 | 1 | 1 | 4 | 0 | 2 | 0 | 0 | 0 | 0 | 0 |
| 2016 | 0 | 0 | 1 | 0 | 1 | 2 | 0 | 2 | 0 | 0 | 0 | 0 | 0 |
| 2016 | 0 | 3 | 0 | 1 | 1 | 0 | 0 | 2 | 0 | 1 | 0 | 0 | 0 |
| 2016 | 1 | 0 | 1 | 1 | 0 | 2 | 0 | 2 | 0 | 0 | 0 | 0 | 0 |
| 2016 | 1 | 0 | 0 | 1 | 0 | 3 | 0 | 2 | 0 | 1 | 0 | 0 | 0 |
| 2016 | 0 | 3 | 3 | 1 | 0 | 2 | 0 | 2 | 0 | 0 | 0 | 0 | 0 |
| 2016 | 1 | 4 | 2 | 0 | 0 | 1 | 1 | 2 | 0 | 1 | 0 | 0 | 0 |
| 2016 | 1 | 3 | 0 | 0 | 0 | 2 | 0 | 2 | 0 | 0 | 0 | 0 | 0 |
| 2016 | 1 | 0 | 0 | 1 | 1 | 1 | 0 | 2 | 0 | 0 | 0 | 0 | 0 |
| 2016 | 0 | 4 | 3 | 2 | 1 | 0 | 0 | 2 | 1 | 1 | 0 | 0 | 0 |
| 2016 | 0 | 4 | 4 | 2 | 0 | 3 | 0 | 2 | 0 | 0 | 0 | 0 | 0 |
| 2016 | 1 | 2 | 2 | 1 | 0 | 2 | 0 | 2 | 0 | 1 | 0 | 0 | 0 |
| 2016 | 1 | 3 | 0 | 1 | 1 | 4 | 0 | 2 | 0 | 0 | 0 | 0 | 0 |
| 2016 | 1 | 1 | 0 | 1 | 0 | 4 | 0 | 2 | 0 | 0 | 0 | 0 | 0 |
| 2016 | 0 | 1 | 0 | 1 | 0 | 4 | 0 | 2 | 0 | 0 | 0 | 0 | 0 |
| 2016 | 0 | 1 | 4 | 1 | 0 | 4 | 0 | 2 | 0 | 0 | 0 | 1 | 0 |
| 2016 | 0 | 0 | 0 | 1 | 0 | 4 | 0 | 2 | 0 | 0 | 0 | 0 | 0 |
| 2016 | 0 | 0 | 0 | 1 | 1 | 4 | 0 | 2 | 0 | 0 | 0 | 0 | 0 |
| 2016 | 1 | 3 | 0 | 1 | 0 | 4 | 0 | 2 | 0 | 0 | 0 | 0 | 0 |
| 2016 | 0 | 2 | 0 | 1 | 1 | 4 | 0 | 2 | 0 | 0 | 0 | 0 | 0 |
| 2016 | 1 | 1 | 1 | 1 | 0 | 4 | 0 | 2 | 0 | 0 | 0 | 0 | 0 |
| 2016 | 1 | 1 | 1 | 1 | 0 | 3 | 0 | 2 | 0 | 0 | 0 | 0 | 0 |
| 2016 | 0 | 2 | 0 | 0 | 1 | 3 | 0 | 2 | 0 | 0 | 0 | 0 | 0 |
| 2016 | 0 | 1 | 0 | 1 | 0 | 3 | 0 | 2 | 0 | 0 | 0 | 0 | 0 |
| 2016 | 0 | 3 | 0 | 1 | 1 | 3 | 0 | 2 | 0 | 0 | 0 | 0 | 0 |
| 2016 | 0 | 2 | 1 | 1 | 1 | 3 | 0 | 2 | 0 | 1 | 0 | 0 | 0 |
| 2016 | 0 | 0 | 0 | 1 | 0 | 3 | 0 | 2 | 0 | 0 | 0 | 0 | 0 |
| 2016 | 0 | 1 | 4 | 1 | 0 | 3 | 0 | 2 | 0 | 0 | 0 | 0 | 0 |
| 2016 | 1 | 4 | 4 | 1 | 0 | 3 | 0 | 2 | 0 | 0 | 0 | 0 | 0 |
| 2016 | 1 | 3 | 1 | 1 | 1 | 3 | 0 | 2 | 0 | 1 | 0 | 0 | 0 |
| 2016 | 1 | 1 | 0 | 1 | 0 | 3 | 0 | 2 | 0 | 0 | 0 | 0 | 0 |
| 2016 | 1 | 3 | 2 | 1 | 0 | 3 | 0 | 2 | 0 | 0 | 0 | 0 | 0 |
| 2016 | 1 | 2 | 2 | 2 | 0 | 3 | 0 | 2 | 0 | 1 | 0 | 0 | 0 |
| 2016 | 1 | 1 | 1 | 1 | 0 | 2 | 1 | 2 | 0 | 1 | 0 | 0 | 0 |
| 2016 | 1 | 2 | 1 | 1 | 0 | 2 | 0 | 2 | 0 | 1 | 0 | 0 | 0 |
| 2016 | 1 | 0 | 0 | 1 | 0 | 2 | 0 | 2 | 0 | 0 | 0 | 0 | 0 |
| 2016 | 0 | 1 | 0 | 1 | 1 | 2 | 0 | 2 | 0 | 0 | 0 | 0 | 0 |
| 2016 | 0 | 3 | 3 | 1 | 0 | 2 | 1 | 2 | 0 | 1 | 0 | 0 | 0 |
| 2016 | 0 | 0 | 1 | 1 | 0 | 2 | 0 | 2 | 0 | 1 | 0 | 1 | 0 |
| 2016 | 1 | 0 | 4 | 0 | 0 | 2 | 0 | 2 | 0 | 0 | 0 | 0 | 0 |
| 2016 | 1 | 2 | 4 | 1 | 0 | 1 | 0 | 2 | 0 | 0 | 0 | 0 | 0 |
| 2016 | 0 | 2 | 1 | 1 | 1 | 1 | 0 | 2 | 0 | 1 | 0 | 0 | 0 |
| 2016 | 1 | 3 | 3 | 1 | 0 | 1 | 0 | 2 | 0 | 1 | 0 | 0 | 0 |
| 2016 | 0 | 2 | 1 | 2 | 0 | 1 | 0 | 2 | 0 | 1 | 0 | 0 | 0 |
| 2016 | 0 | 0 | 1 | 1 | 0 | 1 | 0 | 2 | 0 | 0 | 0 | 0 | 0 |
| 2016 | 1 | 3 | 1 | 1 | 1 | 0 | 0 | 2 | 1 | 1 | 0 | 0 | 0 |
| 2016 | 0 | 3 | 4 | 1 | 1 | 0 | 0 | 2 | 0 | 0 | 0 | 0 | 0 |
| 2016 | 1 | 3 | 4 | 1 | 1 | 0 | 0 | 2 | 1 | 1 | 0 | 0 | 0 |
| 2016 | 0 | 1 | 0 | 1 | 1 | 0 | 0 | 2 | 0 | 0 | 0 | 0 | 0 |
| 2016 | 1 | 0 | 0 | 2 | 1 | 0 | 0 | 2 | 0 | 0 | 0 | 0 | 0 |
| 2016 | 0 | 1 | 0 | 1 | 0 | 4 | 0 | 2 | 0 | 0 | 0 | 0 | 0 |
| 2016 | 0 | 0 | 1 | 1 | 0 | 3 | 0 | 2 | 0 | 0 | 0 | 0 | 0 |
| 2016 | 0 | 0 | 0 | 1 | 0 | 1 | 0 | 2 | 0 | 1 | 0 | 0 | 0 |
| 2016 | 1 | 2 | 0 | 2 | 1 | 3 | 0 | 2 | 0 | 0 | 0 | 0 | 0 |
| 2016 | 0 | 1 | 1 | 1 | 0 | 3 | 0 | 2 | 0 | 1 | 0 | 0 | 0 |
| 2016 | 1 | 1 | 0 | 1 | 0 | 4 | 0 | 2 | 0 | 0 | 0 | 0 | 0 |
| 2016 | 0 | 1 | 0 | 2 | 0 | 4 | 0 | 2 | 0 | 0 | 0 | 0 | 0 |
| 2016 | 1 | 3 | 1 | 1 | 0 | 3 | 0 | 2 | 0 | 0 | 0 | 0 | 0 |
| 2016 | 1 | 2 | 1 | 1 | 0 | 3 | 0 | 2 | 0 | 0 | 0 | 0 | 0 |
| 2016 | 1 | 0 | 1 | 1 | 0 | 2 | 0 | 2 | 0 | 1 | 0 | 0 | 0 |
| 2016 | 0 | 0 | 1 | 1 | 0 | 1 | 0 | 2 | 0 | 1 | 0 | 0 | 0 |
| 2016 | 0 | 0 | 4 | 1 | 0 | 3 | 0 | 2 | 0 | 0 | 0 | 0 | 0 |
| 2016 | 1 | 3 | 4 | 2 | 0 | 1 | 0 | 2 | 0 | 1 | 0 | 0 | 0 |
| 2016 | 0 | 2 | 0 | 0 | 1 | 0 | 0 | 2 | 0 | 0 | 0 | 0 | 0 |
| 2016 | 1 | 1 | 1 | 1 | 1 | 0 | 0 | 2 | 0 | 1 | 0 | 0 | 0 |
| 2016 | 1 | 4 | 0 | 1 | 0 | 4 | 0 | 2 | 0 | 0 | 0 | 0 | 0 |
| 2016 | 1 | 3 | 0 | 2 | 0 | 3 | 0 | 2 | 0 | 0 | 0 | 0 | 0 |
| 2016 | 0 | 2 | 4 | 1 | 0 | 2 | 0 | 2 | 0 | 1 | 0 | 0 | 0 |
| 2016 | 0 | 0 | 1 | 0 | 0 | 1 | 0 | 2 | 0 | 1 | 0 | 0 | 0 |
| 2016 | 0 | 0 | 1 | 1 | 0 | 1 | 0 | 2 | 0 | 1 | 0 | 0 | 0 |
| 2016 | 1 | 1 | 0 | 1 | 0 | 4 | 0 | 2 | 0 | 0 | 0 | 0 | 0 |
| 2016 | 1 | 1 | 1 | 1 | 0 | 4 | 0 | 2 | 0 | 0 | 0 | 0 | 0 |
| 2016 | 1 | 1 | 1 | 1 | 0 | 2 | 0 | 2 | 0 | 1 | 0 | 0 | 0 |
| 2016 | 1 | 2 | 0 | 2 | 1 | 2 | 0 | 2 | 0 | 1 | 0 | 0 | 0 |
| 2016 | 0 | 0 | 1 | 2 | 0 | 2 | 0 | 2 | 0 | 0 | 0 | 0 | 0 |
| 2016 | 0 | 1 | 0 | 1 | 0 | 4 | 0 | 2 | 0 | 0 | 0 | 0 | 0 |
| 2016 | 0 | 2 | 1 | 0 | 1 | 3 | 0 | 2 | 0 | 0 | 0 | 0 | 0 |
| 2016 | 1 | 3 | 3 | 1 | 0 | 3 | 0 | 2 | 0 | 1 | 0 | 0 | 0 |
| 2016 | 0 | 3 | 1 | 1 | 1 | 3 | 0 | 2 | 0 | 0 | 0 | 0 | 0 |
| 2016 | 0 | 4 | 4 | 2 | 1 | 2 | 0 | 2 | 0 | 0 | 0 | 0 | 0 |
| 2016 | 1 | 3 | 3 | 1 | 0 | 2 | 0 | 2 | 0 | 1 | 0 | 0 | 0 |
| 2016 | 1 | 0 | 4 | 1 | 0 | 2 | 0 | 2 | 0 | 0 | 0 | 0 | 0 |
| 2016 | 0 | 1 | 1 | 0 | 0 | 2 | 0 | 2 | 0 | 0 | 0 | 0 | 0 |
| 2016 | 1 | 1 | 1 | 1 | 0 | 2 | 0 | 2 | 0 | 1 | 0 | 0 | 0 |
| 2016 | 0 | 1 | 1 | 1 | 0 | 2 | 0 | 2 | 0 | 1 | 0 | 0 | 0 |
| 2016 | 0 | 1 | 0 | 1 | 0 | 2 | 0 | 2 | 0 | 0 | 0 | 0 | 0 |
| 2016 | 1 | 0 | 1 | 2 | 1 | 2 | 0 | 2 | 0 | 0 | 0 | 0 | 0 |
| 2016 | 0 | 0 | 4 | 1 | 0 | 1 | 0 | 2 | 0 | 0 | 0 | 0 | 0 |
| 2016 | 0 | 4 | 4 | 1 | 0 | 1 | 0 | 2 | 0 | 1 | 0 | 0 | 0 |
| 2016 | 1 | 3 | 1 | 1 | 0 | 1 | 0 | 2 | 0 | 1 | 0 | 0 | 0 |
| 2016 | 1 | 1 | 2 | 1 | 0 | 1 | 0 | 2 | 0 | 1 | 0 | 0 | 0 |
| 2016 | 1 | 0 | 1 | 1 | 0 | 1 | 0 | 2 | 0 | 0 | 0 | 0 | 0 |
| 2016 | 1 | 3 | 0 | 1 | 1 | 0 | 0 | 2 | 0 | 1 | 0 | 0 | 0 |
| 2016 | 1 | 1 | 0 | 1 | 1 | 0 | 0 | 2 | 0 | 0 | 0 | 0 | 0 |
| 2016 | 0 | 2 | 1 | 1 | 1 | 0 | 0 | 2 | 0 | 1 | 1 | 0 | 0 |
| 2016 | 1 | 2 | 2 | 1 | 1 | 0 | 0 | 2 | 0 | 1 | 1 | 0 | 0 |
| 2016 | 0 | 0 | 0 | 1 | 0 | 3 | 0 | 2 | 0 | 0 | 0 | 0 | 0 |
| 2016 | 0 | 1 | 0 | 1 | 0 | 4 | 0 | 2 | 0 | 1 | 0 | 0 | 0 |
| 2016 | 0 | 2 | 4 | 1 | 0 | 1 | 0 | 2 | 0 | 1 | 0 | 0 | 0 |
| 2016 | 1 | 3 | 2 | 0 | 0 | 1 | 0 | 2 | 0 | 1 | 0 | 0 | 0 |
| 2016 | 1 | 2 | 0 | 1 | 0 | 3 | 0 | 2 | 0 | 0 | 0 | 0 | 0 |
| 2016 | 0 | 3 | 4 | 1 | 0 | 1 | 0 | 2 | 0 | 0 | 0 | 0 | 0 |
| 2016 | 1 | 1 | 0 | 1 | 0 | 4 | 0 | 2 | 0 | 0 | 0 | 0 | 0 |
| 2016 | 0 | 4 | 1 | 1 | 1 | 4 | 0 | 2 | 0 | 0 | 0 | 0 | 0 |
| 2016 | 1 | 1 | 4 | 1 | 0 | 3 | 0 | 2 | 0 | 0 | 0 | 0 | 0 |
| 2016 | 0 | 2 | 1 | 2 | 0 | 1 | 0 | 2 | 0 | 1 | 0 | 1 | 0 |
| 2016 | 0 | 3 | 3 | 1 | 1 | 1 | 0 | 2 | 0 | 1 | 0 | 0 | 0 |
| 2016 | 0 | 2 | 1 | 1 | 0 | 0 | 0 | 2 | 0 | 0 | 0 | 0 | 0 |
| 2016 | 0 | 2 | 3 | 1 | 0 | 2 | 0 | 2 | 0 | 0 | 0 | 0 | 0 |
| 2016 | 0 | 3 | 1 | 1 | 0 | 2 | 0 | 2 | 0 | 1 | 0 | 0 | 0 |
| 2016 | 0 | 1 | 0 | 1 | 1 | 1 | 0 | 2 | 0 | 1 | 0 | 0 | 0 |
| 2016 | 1 | 3 | 2 | 1 | 1 | 0 | 0 | 2 | 0 | 1 | 0 | 0 | 0 |
| 2016 | 1 | 1 | 4 | 2 | 0 | 2 | 0 | 2 | 0 | 0 | 0 | 0 | 0 |
| 2016 | 1 | 1 | 0 | 0 | 1 | 0 | 0 | 2 | 0 | 1 | 0 | 0 | 0 |
| 2016 | 1 | 3 | 2 | 1 | 0 | 2 | 0 | 2 | 0 | 1 | 0 | 1 | 0 |
| 2016 | 0 | 1 | 3 | 1 | 0 | 1 | 0 | 2 | 1 | 1 | 0 | 0 | 0 |
| 2016 | 1 | 2 | 2 | 1 | 0 | 1 | 0 | 2 | 0 | 1 | 0 | 0 | 0 |
| 2016 | 0 | 1 | 2 | 1 | 0 | 1 | 0 | 2 | 0 | 1 | 0 | 0 | 0 |
| 2016 | 0 | 3 | 1 | 1 | 1 | 1 | 0 | 2 | 0 | 1 | 0 | 0 | 0 |
| 2016 | 0 | 1 | 1 | 1 | 0 | 1 | 0 | 2 | 0 | 1 | 0 | 0 | 0 |
| 2016 | 1 | 2 | 3 | 1 | 0 | 2 | 0 | 2 | 1 | 1 | 0 | 0 | 0 |
| 2016 | 0 | 1 | 1 | 1 | 0 | 1 | 0 | 2 | 0 | 1 | 0 | 0 | 0 |
| 2016 | 0 | 2 | 3 | 1 | 0 | 1 | 0 | 2 | 0 | 1 | 0 | 0 | 0 |
| 2016 | 0 | 1 | 3 | 1 | 1 | 1 | 0 | 2 | 0 | 1 | 0 | 0 | 0 |
| 2016 | 0 | 1 | 3 | 1 | 0 | 1 | 0 | 2 | 0 | 1 | 0 | 0 | 0 |
| 2016 | 1 | 3 | 3 | 1 | 1 | 0 | 0 | 2 | 1 | 1 | 0 | 0 | 0 |
| 2016 | 0 | 2 | 3 | 1 | 0 | 1 | 0 | 2 | 1 | 1 | 0 | 0 | 0 |
| 2016 | 0 | 1 | 3 | 1 | 0 | 1 | 0 | 2 | 0 | 1 | 0 | 0 | 0 |
| 2016 | 1 | 1 | 2 | 1 | 0 | 2 | 0 | 2 | 0 | 1 | 0 | 0 | 0 |
| 2016 | 0 | 1 | 1 | 1 | 1 | 2 | 0 | 2 | 0 | 1 | 0 | 0 | 0 |
| 2016 | 0 | 2 | 3 | 1 | 0 | 2 | 0 | 2 | 1 | 1 | 0 | 0 | 0 |
| 2016 | 0 | 2 | 3 | 1 | 0 | 0 | 0 | 2 | 0 | 1 | 0 | 0 | 0 |
| 2016 | 0 | 1 | 3 | 1 | 0 | 2 | 0 | 2 | 0 | 1 | 0 | 0 | 0 |
| 2016 | 0 | 1 | 2 | 1 | 0 | 1 | 0 | 2 | 0 | 1 | 0 | 0 | 0 |
| 2016 | 0 | 1 | 2 | 1 | 0 | 2 | 0 | 2 | 0 | 1 | 0 | 0 | 0 |
| 2016 | 0 | 1 | 2 | 1 | 0 | 1 | 0 | 2 | 0 | 1 | 0 | 0 | 0 |
| 2016 | 1 | 2 | 3 | 1 | 0 | 2 | 0 | 2 | 1 | 1 | 0 | 0 | 0 |
| 2016 | 1 | 3 | 3 | 1 | 0 | 1 | 0 | 2 | 1 | 1 | 0 | 0 | 0 |
| 2016 | 0 | 1 | 3 | 1 | 1 | 2 | 0 | 2 | 0 | 1 | 0 | 0 | 0 |
| 2016 | 1 | 1 | 3 | 1 | 0 | 1 | 0 | 2 | 0 | 1 | 0 | 0 | 0 |
| 2016 | 0 | 3 | 1 | 1 | 0 | 1 | 0 | 2 | 0 | 1 | 0 | 0 | 0 |
| 2016 | 0 | 1 | 1 | 1 | 0 | 2 | 0 | 2 | 0 | 1 | 0 | 0 | 0 |
| 2016 | 0 | 1 | 2 | 1 | 0 | 1 | 0 | 2 | 0 | 1 | 0 | 0 | 0 |
| 2016 | 1 | 1 | 3 | 1 | 0 | 1 | 0 | 2 | 0 | 1 | 0 | 0 | 0 |
| 2016 | 0 | 0 | 3 | 1 | 0 | 1 | 0 | 2 | 0 | 1 | 0 | 0 | 0 |
| 2016 | 1 | 2 | 3 | 1 | 0 | 2 | 0 | 2 | 0 | 1 | 0 | 0 | 0 |
| 2016 | 0 | 1 | 1 | 1 | 0 | 2 | 0 | 2 | 0 | 1 | 0 | 0 | 0 |
| 2016 | 0 | 1 | 3 | 1 | 0 | 1 | 0 | 2 | 1 | 1 | 0 | 0 | 0 |
| 2016 | 1 | 3 | 3 | 1 | 0 | 2 | 0 | 2 | 1 | 1 | 0 | 0 | 0 |
| 2016 | 0 | 1 | 2 | 1 | 0 | 2 | 0 | 2 | 0 | 1 | 0 | 0 | 0 |
| 2016 | 0 | 1 | 2 | 1 | 0 | 1 | 0 | 2 | 0 | 1 | 0 | 0 | 0 |
| 2016 | 0 | 1 | 3 | 1 | 1 | 1 | 0 | 2 | 0 | 1 | 0 | 0 | 0 |
| 2016 | 1 | 2 | 1 | 1 | 0 | 1 | 0 | 2 | 0 | 1 | 0 | 0 | 0 |
| 2016 | 0 | 0 | 1 | 1 | 0 | 2 | 0 | 2 | 0 | 1 | 0 | 0 | 0 |
| 2016 | 1 | 0 | 1 | 1 | 0 | 1 | 0 | 2 | 0 | 1 | 0 | 0 | 0 |
| 2016 | 1 | 1 | 3 | 1 | 0 | 2 | 0 | 2 | 0 | 1 | 0 | 0 | 0 |
| 2016 | 0 | 1 | 3 | 1 | 0 | 1 | 0 | 2 | 0 | 1 | 0 | 0 | 0 |
| 2016 | 1 | 1 | 3 | 1 | 0 | 2 | 0 | 2 | 0 | 1 | 0 | 0 | 0 |
| 2016 | 0 | 1 | 3 | 1 | 0 | 1 | 0 | 2 | 0 | 1 | 0 | 0 | 0 |
| 2016 | 0 | 0 | 3 | 1 | 0 | 1 | 0 | 2 | 0 | 1 | 0 | 0 | 0 |
| 2016 | 1 | 1 | 3 | 1 | 1 | 2 | 0 | 2 | 0 | 1 | 0 | 0 | 0 |
| 2016 | 0 | 1 | 1 | 1 | 1 | 1 | 0 | 2 | 0 | 1 | 0 | 0 | 0 |
| 2016 | 0 | 1 | 1 | 1 | 0 | 1 | 0 | 2 | 0 | 1 | 0 | 0 | 0 |
| 2016 | 1 | 1 | 3 | 1 | 1 | 2 | 0 | 2 | 0 | 1 | 0 | 0 | 0 |
| 2016 | 0 | 1 | 3 | 1 | 0 | 3 | 0 | 2 | 0 | 1 | 0 | 0 | 0 |
| 2016 | 0 | 1 | 3 | 1 | 0 | 1 | 0 | 2 | 0 | 1 | 0 | 0 | 0 |
| 2016 | 1 | 2 | 3 | 1 | 0 | 1 | 0 | 2 | 0 | 1 | 0 | 0 | 0 |
| 2016 | 1 | 3 | 0 | 1 | 0 | 4 | 0 | 0 | 0 | 0 | 0 | 0 | 0 |
| 2016 | 0 | 3 | 3 | 1 | 1 | 3 | 0 | 0 | 0 | 1 | 0 | 0 | 0 |
| 2016 | 0 | 4 | 1 | 1 | 1 | 3 | 0 | 0 | 0 | 1 | 0 | 0 | 0 |
| 2016 | 0 | 1 | 4 | 1 | 0 | 3 | 0 | 0 | 0 | 0 | 0 | 0 | 0 |
| 2016 | 0 | 4 | 4 | 1 | 1 | 3 | 0 | 0 | 0 | 0 | 0 | 0 | 0 |
| 2016 | 0 | 1 | 1 | 1 | 0 | 2 | 0 | 1 | 0 | 0 | 0 | 0 | 0 |
| 2016 | 1 | 1 | 1 | 1 | 0 | 3 | 1 | 0 | 0 | 1 | 0 | 0 | 0 |
| 2016 | 1 | 3 | 1 | 1 | 0 | 0 | 0 | 1 | 0 | 1 | 0 | 0 | 0 |
| 2016 | 0 | 1 | 0 | 0 | 0 | 1 | 1 | 1 | 0 | 0 | 0 | 1 | 0 |
| 2016 | 0 | 3 | 1 | 1 | 0 | 0 | 0 | 0 | 0 | 0 | 0 | 0 | 0 |
| 2016 | 1 | 4 | 1 | 1 | 0 | 4 | 0 | 0 | 0 | 0 | 0 | 0 | 0 |
| 2016 | 0 | 1 | 1 | 1 | 0 | 2 | 0 | 0 | 0 | 0 | 0 | 0 | 0 |
| 2016 | 1 | 4 | 4 | 1 | 1 | 4 | 0 | 0 | 0 | 0 | 0 | 0 | 0 |
| 2016 | 0 | 1 | 0 | 1 | 0 | 3 | 0 | 0 | 0 | 0 | 0 | 0 | 0 |
| 2016 | 1 | 1 | 4 | 1 | 0 | 3 | 0 | 0 | 0 | 0 | 0 | 0 | 0 |
| 2016 | 0 | 1 | 0 | 1 | 1 | 2 | 0 | 0 | 0 | 0 | 0 | 0 | 0 |
| 2016 | 0 | 0 | 4 | 1 | 1 | 2 | 0 | 0 | 0 | 0 | 0 | 0 | 0 |
| 2016 | 0 | 3 | 1 | 1 | 0 | 0 | 0 | 0 | 0 | 1 | 0 | 0 | 0 |
| 2016 | 1 | 1 | 4 | 1 | 0 | 3 | 0 | 2 | 0 | 0 | 0 | 0 | 0 |
| 2016 | 0 | 1 | 4 | 1 | 0 | 3 | 0 | 2 | 0 | 0 | 0 | 0 | 0 |
| 2016 | 1 | 2 | 4 | 1 | 0 | 1 | 0 | 0 | 0 | 1 | 0 | 0 | 0 |
| 2016 | 1 | 3 | 4 | 1 | 0 | 4 | 0 | 0 | 0 | 0 | 0 | 0 | 0 |
| 2016 | 1 | 0 | 4 | 1 | 0 | 3 | 0 | 0 | 0 | 0 | 0 | 0 | 0 |
| 2016 | 0 | 1 | 1 | 1 | 0 | 3 | 0 | 0 | 0 | 0 | 0 | 0 | 0 |
| 2016 | 0 | 1 | 2 | 1 | 0 | 1 | 1 | 1 | 0 | 1 | 0 | 0 | 0 |
| 2016 | 1 | 1 | 0 | 1 | 1 | 4 | 0 | 0 | 0 | 0 | 0 | 0 | 0 |
| 2016 | 0 | 0 | 0 | 1 | 0 | 3 | 0 | 0 | 0 | 0 | 1 | 0 | 0 |
| 2016 | 0 | 0 | 1 | 1 | 1 | 3 | 0 | 2 | 0 | 0 | 0 | 0 | 0 |
| 2016 | 1 | 3 | 1 | 1 | 0 | 2 | 0 | 0 | 0 | 0 | 0 | 0 | 0 |
| 2016 | 1 | 3 | 4 | 1 | 0 | 2 | 0 | 0 | 0 | 0 | 0 | 0 | 0 |
| 2016 | 1 | 0 | 4 | 0 | 1 | 0 | 0 | 0 | 0 | 0 | 0 | 0 | 0 |
| 2016 | 0 | 3 | 1 | 1 | 0 | 4 | 0 | 0 | 0 | 0 | 0 | 0 | 0 |
| 2016 | 1 | 0 | 0 | 1 | 0 | 3 | 0 | 0 | 0 | 0 | 0 | 0 | 0 |
| 2016 | 0 | 3 | 4 | 1 | 0 | 3 | 0 | 0 | 0 | 1 | 0 | 0 | 0 |
| 2016 | 1 | 2 | 4 | 0 | 0 | 1 | 0 | 0 | 0 | 0 | 0 | 0 | 1 |
| 2016 | 0 | 3 | 1 | 0 | 0 | 1 | 0 | 0 | 0 | 1 | 0 | 0 | 0 |
| 2016 | 1 | 3 | 4 | 1 | 1 | 1 | 0 | 0 | 0 | 1 | 0 | 0 | 0 |
| 2016 | 1 | 0 | 0 | 0 | 0 | 0 | 0 | 0 | 0 | 0 | 0 | 0 | 0 |
| 2016 | 0 | 0 | 1 | 1 | 1 | 2 | 0 | 0 | 0 | 1 | 0 | 0 | 0 |
| 2016 | 1 | 3 | 1 | 0 | 1 | 0 | 0 | 1 | 0 | 1 | 0 | 0 | 0 |
| 2016 | 0 | 2 | 0 | 0 | 1 | 0 | 0 | 1 | 0 | 0 | 0 | 0 | 0 |
| 2016 | 1 | 0 | 0 | 1 | 0 | 2 | 0 | 0 | 0 | 1 | 0 | 0 | 0 |
| 2016 | 1 | 1 | 3 | 1 | 1 | 3 | 0 | 0 | 0 | 1 | 0 | 0 | 0 |
| 2016 | 1 | 1 | 4 | 1 | 0 | 0 | 0 | 0 | 0 | 0 | 0 | 0 | 0 |
| 2016 | 1 | 1 | 0 | 0 | 1 | 0 | 0 | 0 | 0 | 1 | 0 | 0 | 0 |
| 2016 | 0 | 0 | 4 | 1 | 0 | 4 | 0 | 2 | 0 | 0 | 0 | 0 | 1 |
| 2016 | 1 | 2 | 1 | 1 | 1 | 4 | 0 | 0 | 0 | 1 | 0 | 0 | 0 |
| 2016 | 0 | 2 | 0 | 1 | 1 | 3 | 0 | 0 | 0 | 0 | 1 | 0 | 0 |
| 2016 | 0 | 1 | 4 | 1 | 1 | 3 | 0 | 0 | 0 | 0 | 0 | 0 | 0 |
| 2016 | 1 | 2 | 1 | 1 | 1 | 3 | 0 | 0 | 0 | 0 | 0 | 0 | 0 |
| 2016 | 1 | 1 | 1 | 1 | 0 | 3 | 0 | 0 | 0 | 1 | 0 | 0 | 0 |
| 2016 | 1 | 2 | 1 | 1 | 0 | 3 | 0 | 0 | 0 | 1 | 0 | 0 | 0 |
| 2016 | 1 | 3 | 1 | 1 | 0 | 3 | 0 | 0 | 0 | 1 | 0 | 0 | 0 |
| 2016 | 1 | 1 | 4 | 1 | 0 | 3 | 1 | 0 | 0 | 1 | 0 | 0 | 0 |
| 2016 | 1 | 3 | 3 | 1 | 0 | 3 | 0 | 0 | 0 | 1 | 0 | 0 | 0 |
| 2016 | 0 | 3 | 0 | 0 | 0 | 2 | 0 | 0 | 0 | 0 | 0 | 0 | 1 |
| 2016 | 0 | 0 | 0 | 1 | 1 | 2 | 0 | 0 | 0 | 0 | 0 | 0 | 0 |
| 2016 | 1 | 2 | 4 | 1 | 1 | 2 | 0 | 0 | 0 | 1 | 0 | 0 | 0 |
| 2016 | 1 | 4 | 1 | 1 | 0 | 2 | 0 | 0 | 0 | 1 | 0 | 0 | 0 |
| 2016 | 0 | 2 | 0 | 1 | 0 | 2 | 0 | 0 | 0 | 1 | 0 | 0 | 1 |
| 2016 | 1 | 2 | 0 | 0 | 0 | 2 | 0 | 0 | 0 | 1 | 0 | 0 | 0 |
| 2016 | 1 | 0 | 1 | 1 | 0 | 1 | 0 | 0 | 0 | 1 | 0 | 0 | 0 |
| 2016 | 0 | 4 | 1 | 0 | 1 | 1 | 0 | 0 | 0 | 0 | 0 | 0 | 0 |
| 2016 | 1 | 2 | 1 | 0 | 0 | 0 | 0 | 0 | 0 | 0 | 0 | 0 | 0 |
| 2016 | 0 | 3 | 1 | 1 | 0 | 0 | 0 | 0 | 0 | 1 | 2 | 2 | 2 |
| 2016 | 0 | 2 | 1 | 1 | 0 | 0 | 0 | 0 | 0 | 0 | 1 | 0 | 0 |
| 2016 | 0 | 4 | 4 | 1 | 0 | 3 | 0 | 0 | 0 | 0 | 0 | 0 | 0 |
| 2016 | 0 | 0 | 0 | 1 | 0 | 3 | 0 | 0 | 0 | 0 | 0 | 0 | 0 |
| 2016 | 0 | 0 | 4 | 1 | 0 | 3 | 0 | 0 | 0 | 0 | 0 | 0 | 0 |
| 2016 | 0 | 4 | 4 | 1 | 0 | 3 | 0 | 2 | 0 | 0 | 0 | 0 | 0 |
| 2016 | 1 | 3 | 3 | 1 | 1 | 2 | 0 | 2 | 0 | 1 | 0 | 0 | 0 |
| 2016 | 0 | 4 | 4 | 0 | 1 | 0 | 0 | 0 | 0 | 1 | 0 | 0 | 0 |
| 2016 | 1 | 0 | 1 | 1 | 0 | 0 | 0 | 0 | 0 | 1 | 0 | 0 | 0 |
| 2016 | 1 | 2 | 4 | 0 | 0 | 1 | 0 | 0 | 0 | 0 | 0 | 0 | 1 |
| 2016 | 1 | 3 | 4 | 1 | 1 | 4 | 0 | 0 | 0 | 0 | 0 | 0 | 0 |
| 2016 | 0 | 4 | 4 | 1 | 0 | 4 | 0 | 2 | 0 | 0 | 0 | 0 | 0 |
| 2016 | 0 | 4 | 4 | 1 | 0 | 1 | 0 | 0 | 0 | 0 | 0 | 0 | 0 |
| 2016 | 1 | 1 | 4 | 1 | 1 | 0 | 0 | 0 | 0 | 0 | 0 | 0 | 0 |
| 2016 | 0 | 0 | 0 | 1 | 1 | 4 | 0 | 0 | 0 | 0 | 0 | 0 | 0 |
| 2016 | 0 | 3 | 1 | 1 | 1 | 2 | 0 | 0 | 0 | 1 | 0 | 0 | 0 |
| 2016 | 0 | 1 | 0 | 1 | 0 | 1 | 0 | 1 | 0 | 0 | 0 | 0 | 0 |
| 2016 | 0 | 1 | 4 | 1 | 0 | 4 | 0 | 0 | 0 | 0 | 0 | 0 | 0 |
| 2016 | 0 | 0 | 4 | 1 | 0 | 3 | 0 | 0 | 0 | 0 | 0 | 0 | 0 |
| 2016 | 1 | 2 | 1 | 1 | 0 | 3 | 0 | 0 | 0 | 0 | 0 | 0 | 0 |
| 2016 | 0 | 1 | 1 | 1 | 0 | 2 | 0 | 0 | 0 | 0 | 0 | 0 | 0 |
| 2016 | 1 | 0 | 0 | 1 | 1 | 2 | 0 | 0 | 0 | 0 | 0 | 0 | 0 |
| 2016 | 1 | 3 | 1 | 1 | 0 | 1 | 0 | 2 | 0 | 1 | 0 | 0 | 0 |
| 2016 | 1 | 3 | 1 | 1 | 0 | 1 | 0 | 0 | 0 | 1 | 0 | 0 | 0 |
| 2016 | 1 | 1 | 0 | 1 | 1 | 3 | 0 | 0 | 0 | 0 | 0 | 0 | 0 |
| 2016 | 1 | 1 | 1 | 1 | 0 | 3 | 0 | 0 | 0 | 1 | 0 | 0 | 0 |
| 2016 | 0 | 1 | 0 | 1 | 1 | 3 | 0 | 0 | 0 | 0 | 1 | 0 | 0 |
| 2016 | 0 | 2 | 0 | 1 | 0 | 3 | 0 | 0 | 0 | 0 | 0 | 0 | 0 |
| 2016 | 0 | 1 | 0 | 1 | 0 | 2 | 0 | 0 | 0 | 1 | 0 | 0 | 0 |
| 2016 | 1 | 0 | 4 | 1 | 1 | 2 | 0 | 0 | 0 | 0 | 0 | 0 | 0 |
| 2016 | 0 | 1 | 0 | 1 | 1 | 1 | 0 | 0 | 0 | 1 | 0 | 0 | 0 |
| 2016 | 0 | 2 | 0 | 1 | 1 | 0 | 0 | 0 | 0 | 1 | 0 | 0 | 0 |
| 2016 | 1 | 2 | 1 | 1 | 0 | 3 | 0 | 0 | 0 | 0 | 0 | 0 | 0 |
| 2016 | 0 | 1 | 4 | 1 | 1 | 1 | 0 | 0 | 0 | 0 | 0 | 0 | 0 |
| 2016 | 0 | 2 | 0 | 1 | 1 | 3 | 0 | 0 | 0 | 0 | 0 | 0 | 0 |
| 2016 | 1 | 0 | 1 | 1 | 0 | 3 | 0 | 0 | 0 | 0 | 0 | 0 | 0 |
| 2016 | 0 | 1 | 0 | 1 | 0 | 3 | 0 | 0 | 0 | 0 | 0 | 0 | 0 |
| 2016 | 1 | 2 | 0 | 1 | 0 | 3 | 0 | 0 | 0 | 0 | 0 | 0 | 0 |
| 2016 | 0 | 4 | 4 | 1 | 0 | 4 | 0 | 0 | 0 | 0 | 0 | 0 | 0 |
| 2016 | 1 | 4 | 0 | 1 | 0 | 1 | 1 | 1 | 0 | 1 | 0 | 0 | 1 |
| 2016 | 0 | 1 | 0 | 1 | 1 | 1 | 1 | 0 | 0 | 0 | 0 | 0 | 0 |
| 2016 | 0 | 0 | 4 | 1 | 0 | 4 | 0 | 2 | 0 | 1 | 0 | 0 | 0 |
| 2016 | 1 | 4 | 1 | 1 | 0 | 1 | 0 | 2 | 0 | 1 | 0 | 0 | 0 |
| 2016 | 0 | 1 | 1 | 1 | 0 | 2 | 0 | 0 | 0 | 1 | 0 | 0 | 0 |
| 2016 | 1 | 1 | 4 | 1 | 1 | 2 | 0 | 2 | 0 | 1 | 0 | 0 | 0 |
| 2016 | 1 | 3 | 4 | 1 | 0 | 3 | 0 | 0 | 0 | 0 | 1 | 0 | 0 |
| 2016 | 0 | 4 | 4 | 2 | 1 | 4 | 0 | 2 | 0 | 2 | 2 | 2 | 2 |
| 2016 | 0 | 4 | 4 | 2 | 0 | 1 | 0 | 2 | 0 | 0 | 2 | 2 | 2 |
| 2016 | 1 | 4 | 4 | 1 | 0 | 3 | 0 | 2 | 0 | 2 | 2 | 2 | 2 |
| 2016 | 1 | 1 | 2 | 1 | 0 | 1 | 0 | 2 | 0 | 1 | 0 | 0 | 0 |
| 2016 | 1 | 1 | 0 | 0 | 0 | 2 | 0 | 2 | 0 | 0 | 2 | 2 | 2 |
| 2016 | 0 | 3 | 4 | 2 | 1 | 2 | 0 | 2 | 0 | 1 | 0 | 0 | 0 |
| 2016 | 0 | 4 | 4 | 2 | 0 | 3 | 0 | 2 | 0 | 2 | 0 | 0 | 0 |
| 2016 | 0 | 1 | 1 | 1 | 1 | 1 | 0 | 2 | 0 | 1 | 2 | 2 | 2 |
| 2016 | 0 | 2 | 1 | 1 | 0 | 1 | 0 | 2 | 0 | 1 | 0 | 0 | 0 |
| 2016 | 1 | 4 | 4 | 2 | 0 | 3 | 0 | 2 | 0 | 1 | 2 | 2 | 2 |
| 2016 | 0 | 1 | 0 | 0 | 1 | 3 | 0 | 2 | 0 | 0 | 2 | 2 | 2 |
| 2016 | 0 | 1 | 0 | 1 | 1 | 3 | 0 | 2 | 0 | 0 | 2 | 2 | 2 |
| 2017 | 0 | 1 | 1 | 1 | 1 | 3 | 0 | 0 | 0 | 1 | 2 | 2 | 2 |
| 2017 | 1 | 1 | 1 | 1 | 0 | 4 | 0 | 1 | 0 | 0 | 2 | 2 | 2 |
| 2017 | 1 | 1 | 1 | 1 | 0 | 3 | 1 | 0 | 0 | 0 | 2 | 2 | 2 |
| 2017 | 0 | 2 | 1 | 1 | 1 | 3 | 0 | 2 | 0 | 1 | 2 | 2 | 2 |
| 2017 | 0 | 3 | 1 | 1 | 0 | 4 | 0 | 0 | 0 | 0 | 2 | 2 | 2 |
| 2017 | 1 | 2 | 1 | 1 | 1 | 0 | 0 | 2 | 0 | 1 | 2 | 2 | 2 |
| 2017 | 1 | 4 | 1 | 1 | 0 | 2 | 0 | 1 | 0 | 0 | 2 | 2 | 2 |
| 2017 | 0 | 1 | 1 | 1 | 0 | 0 | 0 | 2 | 0 | 0 | 2 | 2 | 2 |
| 2017 | 0 | 1 | 1 | 1 | 0 | 2 | 0 | 0 | 0 | 0 | 2 | 2 | 2 |
| 2017 | 1 | 1 | 1 | 1 | 0 | 2 | 0 | 0 | 0 | 1 | 2 | 2 | 2 |
| 2017 | 0 | 2 | 1 | 1 | 1 | 2 | 0 | 0 | 0 | 2 | 2 | 2 | 2 |
| 2017 | 0 | 2 | 1 | 1 | 0 | 2 | 0 | 0 | 0 | 1 | 2 | 2 | 2 |
| 2017 | 1 | 1 | 1 | 1 | 0 | 1 | 0 | 1 | 0 | 1 | 2 | 2 | 2 |
| 2017 | 0 | 3 | 1 | 1 | 0 | 2 | 0 | 2 | 0 | 1 | 2 | 2 | 2 |
| 2017 | 0 | 1 | 1 | 1 | 0 | 2 | 0 | 2 | 0 | 0 | 2 | 2 | 2 |
| 2017 | 0 | 3 | 1 | 1 | 0 | 4 | 0 | 2 | 0 | 0 | 2 | 2 | 2 |
| 2017 | 1 | 2 | 1 | 1 | 1 | 0 | 0 | 2 | 0 | 0 | 2 | 2 | 2 |
| 2017 | 0 | 0 | 1 | 1 | 0 | 4 | 0 | 0 | 0 | 0 | 2 | 2 | 2 |
| 2017 | 0 | 3 | 1 | 1 | 1 | 4 | 0 | 0 | 0 | 0 | 2 | 2 | 2 |
| 2017 | 0 | 0 | 1 | 1 | 0 | 2 | 0 | 2 | 0 | 1 | 2 | 2 | 2 |
| 2017 | 0 | 1 | 1 | 1 | 0 | 1 | 0 | 2 | 0 | 1 | 2 | 2 | 2 |
| 2017 | 1 | 2 | 1 | 1 | 1 | 4 | 0 | 0 | 0 | 0 | 2 | 2 | 2 |
| 2017 | 1 | 2 | 1 | 1 | 1 | 3 | 0 | 1 | 0 | 1 | 2 | 2 | 2 |
| 2017 | 1 | 3 | 1 | 1 | 1 | 4 | 0 | 0 | 0 | 0 | 2 | 2 | 2 |
| 2017 | 0 | 3 | 1 | 1 | 0 | 0 | 0 | 2 | 0 | 1 | 2 | 2 | 2 |
| 2017 | 0 | 1 | 1 | 1 | 0 | 2 | 0 | 2 | 0 | 1 | 2 | 2 | 2 |
| 2017 | 1 | 4 | 1 | 1 | 0 | 3 | 0 | 0 | 0 | 1 | 2 | 2 | 2 |
| 2017 | 0 | 1 | 1 | 1 | 0 | 2 | 0 | 2 | 0 | 0 | 2 | 2 | 2 |
| 2017 | 1 | 1 | 1 | 1 | 1 | 2 | 0 | 0 | 0 | 1 | 2 | 2 | 2 |
| 2017 | 1 | 2 | 1 | 1 | 0 | 3 | 0 | 0 | 0 | 0 | 2 | 2 | 2 |
| 2017 | 0 | 3 | 1 | 1 | 1 | 2 | 0 | 0 | 0 | 1 | 2 | 2 | 2 |
| 2017 | 1 | 1 | 1 | 1 | 0 | 2 | 0 | 0 | 0 | 1 | 2 | 2 | 2 |
| 2017 | 1 | 3 | 1 | 1 | 0 | 3 | 0 | 2 | 0 | 0 | 2 | 2 | 2 |
| 2017 | 0 | 4 | 1 | 1 | 0 | 2 | 0 | 2 | 0 | 0 | 2 | 2 | 2 |
| 2017 | 0 | 1 | 1 | 0 | 0 | 1 | 0 | 2 | 0 | 1 | 2 | 2 | 2 |
| 2017 | 1 | 0 | 1 | 1 | 0 | 2 | 0 | 0 | 0 | 1 | 2 | 2 | 2 |
| 2017 | 0 | 4 | 1 | 1 | 0 | 0 | 0 | 2 | 0 | 0 | 2 | 2 | 2 |
| 2017 | 0 | 3 | 2 | 1 | 2 | 3 | 0 | 2 | 0 | 0 | 2 | 2 | 2 |
| 2017 | 0 | 3 | 2 | 1 | 2 | 4 | 0 | 0 | 0 | 0 | 2 | 2 | 2 |
| 2017 | 0 | 3 | 2 | 1 | 0 | 4 | 0 | 0 | 0 | 0 | 2 | 2 | 2 |
| 2017 | 0 | 2 | 2 | 2 | 0 | 0 | 0 | 1 | 0 | 1 | 2 | 2 | 2 |
| 2017 | 0 | 1 | 2 | 1 | 0 | 2 | 0 | 0 | 0 | 0 | 2 | 2 | 2 |
| 2017 | 1 | 1 | 2 | 1 | 0 | 3 | 0 | 2 | 0 | 1 | 2 | 2 | 2 |
| 2017 | 0 | 4 | 2 | 1 | 1 | 3 | 0 | 0 | 2 | 1 | 2 | 2 | 2 |
| 2017 | 1 | 3 | 2 | 1 | 0 | 0 | 0 | 0 | 0 | 1 | 2 | 2 | 2 |
| 2017 | 1 | 2 | 2 | 1 | 0 | 2 | 0 | 0 | 0 | 1 | 2 | 2 | 2 |
| 2017 | 0 | 1 | 0 | 0 | 0 | 3 | 0 | 0 | 0 | 0 | 2 | 2 | 2 |
| 2017 | 1 | 1 | 0 | 1 | 0 | 4 | 0 | 1 | 0 | 0 | 2 | 2 | 2 |
| 2017 | 0 | 1 | 0 | 1 | 1 | 0 | 0 | 2 | 2 | 1 | 2 | 2 | 2 |
| 2017 | 1 | 1 | 0 | 1 | 1 | 3 | 0 | 0 | 0 | 0 | 2 | 2 | 2 |
| 2017 | 0 | 3 | 0 | 1 | 1 | 3 | 0 | 0 | 0 | 0 | 2 | 2 | 2 |
| 2017 | 0 | 3 | 0 | 1 | 1 | 3 | 0 | 1 | 0 | 0 | 2 | 2 | 2 |
| 2017 | 0 | 1 | 0 | 1 | 0 | 4 | 0 | 0 | 0 | 0 | 2 | 2 | 2 |
| 2017 | 1 | 3 | 0 | 1 | 1 | 4 | 0 | 0 | 0 | 1 | 2 | 2 | 2 |
| 2017 | 0 | 2 | 0 | 1 | 1 | 3 | 0 | 2 | 0 | 0 | 2 | 2 | 2 |
| 2017 | 1 | 0 | 0 | 1 | 0 | 1 | 0 | 2 | 0 | 1 | 2 | 2 | 2 |
| 2017 | 0 | 3 | 0 | 1 | 1 | 0 | 0 | 0 | 1 | 0 | 2 | 2 | 2 |
| 2017 | 1 | 3 | 0 | 1 | 1 | 0 | 0 | 0 | 0 | 0 | 2 | 2 | 2 |
| 2017 | 0 | 1 | 0 | 1 | 0 | 4 | 0 | 2 | 0 | 1 | 2 | 2 | 2 |
| 2017 | 1 | 4 | 0 | 1 | 1 | 4 | 0 | 0 | 0 | 2 | 2 | 2 | 2 |
| 2017 | 0 | 1 | 0 | 1 | 0 | 4 | 0 | 1 | 0 | 0 | 2 | 2 | 2 |
| 2017 | 0 | 4 | 0 | 1 | 2 | 3 | 0 | 0 | 0 | 0 | 2 | 2 | 2 |
| 2017 | 0 | 1 | 0 | 1 | 0 | 3 | 0 | 0 | 0 | 0 | 2 | 2 | 2 |
| 2017 | 0 | 1 | 0 | 1 | 0 | 3 | 0 | 2 | 0 | 0 | 2 | 2 | 2 |
| 2017 | 0 | 2 | 0 | 1 | 1 | 0 | 0 | 0 | 0 | 1 | 2 | 2 | 2 |
| 2017 | 0 | 1 | 0 | 1 | 0 | 3 | 0 | 2 | 0 | 0 | 2 | 2 | 2 |
| 2017 | 1 | 1 | 0 | 1 | 0 | 3 | 0 | 0 | 0 | 2 | 2 | 2 | 2 |
| 2017 | 0 | 1 | 0 | 1 | 1 | 1 | 0 | 2 | 0 | 0 | 2 | 2 | 2 |
| 2017 | 1 | 0 | 0 | 1 | 1 | 2 | 0 | 2 | 0 | 1 | 2 | 2 | 2 |
| 2017 | 0 | 0 | 0 | 1 | 0 | 4 | 0 | 0 | 0 | 0 | 2 | 2 | 2 |
| 2017 | 1 | 1 | 0 | 1 | 0 | 4 | 0 | 0 | 0 | 0 | 2 | 2 | 2 |
| 2017 | 0 | 1 | 0 | 1 | 0 | 4 | 0 | 0 | 0 | 0 | 2 | 2 | 2 |
| 2017 | 0 | 4 | 0 | 1 | 0 | 1 | 0 | 2 | 0 | 1 | 2 | 2 | 2 |
| 2017 | 0 | 3 | 0 | 1 | 0 | 3 | 0 | 0 | 0 | 0 | 2 | 2 | 2 |
| 2017 | 1 | 1 | 0 | 1 | 0 | 4 | 1 | 2 | 0 | 0 | 2 | 2 | 2 |
| 2017 | 1 | 1 | 0 | 0 | 0 | 4 | 0 | 0 | 0 | 0 | 2 | 2 | 2 |
| 2017 | 1 | 1 | 1 | 1 | 1 | 2 | 0 | 0 | 0 | 1 | 2 | 2 | 2 |
| 2017 | 1 | 0 | 1 | 1 | 0 | 2 | 0 | 2 | 0 | 1 | 2 | 2 | 2 |
| 2017 | 1 | 1 | 1 | 1 | 1 | 2 | 0 | 2 | 0 | 1 | 2 | 2 | 2 |
| 2017 | 0 | 4 | 1 | 1 | 2 | 3 | 0 | 0 | 0 | 0 | 2 | 2 | 2 |
| 2017 | 1 | 3 | 1 | 0 | 0 | 1 | 0 | 0 | 0 | 1 | 2 | 2 | 2 |
| 2017 | 0 | 1 | 1 | 1 | 1 | 3 | 0 | 0 | 0 | 1 | 2 | 2 | 2 |
| 2017 | 1 | 3 | 2 | 0 | 0 | 3 | 0 | 0 | 0 | 1 | 2 | 2 | 2 |
| 2017 | 1 | 1 | 2 | 0 | 0 | 3 | 0 | 0 | 0 | 1 | 2 | 2 | 2 |
| 2017 | 0 | 1 | 2 | 0 | 0 | 3 | 0 | 0 | 0 | 0 | 2 | 2 | 2 |
| 2017 | 1 | 4 | 3 | 1 | 0 | 4 | 0 | 2 | 0 | 0 | 2 | 2 | 2 |
| 2017 | 1 | 4 | 3 | 1 | 1 | 0 | 0 | 0 | 0 | 1 | 2 | 2 | 2 |
| 2017 | 1 | 3 | 3 | 1 | 0 | 3 | 0 | 0 | 2 | 0 | 2 | 2 | 2 |
| 2017 | 0 | 2 | 3 | 1 | 0 | 0 | 0 | 2 | 0 | 0 | 2 | 2 | 2 |
| 2017 | 1 | 1 | 3 | 1 | 0 | 3 | 0 | 2 | 0 | 0 | 2 | 2 | 2 |
| 2017 | 0 | 2 | 3 | 1 | 1 | 0 | 0 | 0 | 0 | 1 | 2 | 2 | 2 |
| 2017 | 1 | 0 | 3 | 1 | 0 | 3 | 0 | 0 | 0 | 0 | 2 | 2 | 2 |
| 2017 | 0 | 4 | 3 | 1 | 0 | 0 | 0 | 0 | 0 | 1 | 2 | 2 | 2 |
| 2017 | 1 | 0 | 3 | 1 | 0 | 2 | 0 | 0 | 0 | 1 | 2 | 2 | 2 |
| 2017 | 0 | 3 | 3 | 1 | 0 | 3 | 0 | 0 | 0 | 0 | 2 | 2 | 2 |
| 2017 | 1 | 1 | 3 | 0 | 0 | 2 | 0 | 2 | 0 | 1 | 2 | 2 | 2 |
| 2017 | 1 | 4 | 4 | 1 | 2 | 3 | 0 | 2 | 2 | 1 | 2 | 2 | 2 |
| 2017 | 1 | 3 | 4 | 1 | 0 | 0 | 0 | 2 | 0 | 1 | 2 | 2 | 2 |
| 2017 | 0 | 4 | 4 | 1 | 0 | 1 | 0 | 0 | 0 | 0 | 2 | 2 | 2 |
| 2017 | 1 | 2 | 4 | 1 | 0 | 0 | 0 | 0 | 0 | 0 | 2 | 2 | 2 |
| 2017 | 0 | 2 | 4 | 1 | 1 | 0 | 0 | 0 | 0 | 1 | 2 | 2 | 2 |
| 2017 | 1 | 4 | 4 | 1 | 1 | 2 | 0 | 0 | 0 | 0 | 2 | 2 | 2 |
| 2017 | 1 | 4 | 4 | 1 | 1 | 0 | 0 | 0 | 0 | 2 | 2 | 2 | 2 |
| 2017 | 0 | 0 | 4 | 1 | 0 | 2 | 0 | 2 | 0 | 0 | 2 | 2 | 2 |
| 2017 | 1 | 3 | 4 | 1 | 1 | 0 | 0 | 2 | 0 | 1 | 2 | 2 | 2 |
| 2017 | 0 | 1 | 4 | 1 | 1 | 0 | 0 | 2 | 0 | 1 | 2 | 2 | 2 |
| 2017 | 0 | 4 | 4 | 1 | 1 | 3 | 0 | 0 | 0 | 0 | 2 | 2 | 2 |
| 2017 | 0 | 0 | 4 | 1 | 1 | 2 | 0 | 0 | 0 | 2 | 2 | 2 | 2 |
| 2017 | 0 | 4 | 4 | 1 | 0 | 1 | 0 | 2 | 0 | 2 | 2 | 2 | 2 |
| 2017 | 0 | 3 | 4 | 1 | 1 | 0 | 0 | 2 | 0 | 2 | 2 | 2 | 2 |
| 2017 | 0 | 4 | 4 | 0 | 1 | 0 | 0 | 2 | 0 | 1 | 2 | 2 | 2 |
| 2017 | 0 | 4 | 4 | 1 | 0 | 3 | 0 | 2 | 0 | 1 | 2 | 2 | 2 |
| 2017 | 1 | 3 | 4 | 1 | 1 | 2 | 0 | 0 | 0 | 1 | 2 | 2 | 2 |
| 2017 | 1 | 2 | 4 | 1 | 0 | 1 | 0 | 1 | 0 | 1 | 2 | 2 | 2 |
| 2017 | 0 | 2 | 4 | 1 | 1 | 3 | 0 | 2 | 0 | 0 | 2 | 2 | 2 |
| 2017 | 0 | 1 | 4 | 1 | 1 | 3 | 0 | 2 | 0 | 0 | 2 | 2 | 2 |
| 2017 | 1 | 3 | 4 | 1 | 0 | 3 | 0 | 0 | 0 | 0 | 2 | 2 | 2 |
| 2017 | 0 | 1 | 4 | 1 | 0 | 2 | 0 | 2 | 0 | 1 | 2 | 2 | 2 |
| 2017 | 1 | 2 | 4 | 1 | 0 | 3 | 0 | 0 | 0 | 0 | 2 | 2 | 2 |
| 2017 | 0 | 1 | 4 | 1 | 0 | 3 | 0 | 2 | 0 | 1 | 2 | 2 | 2 |
| 2017 | 0 | 3 | 4 | 1 | 0 | 3 | 0 | 2 | 0 | 0 | 2 | 2 | 2 |
| 2017 | 1 | 1 | 4 | 1 | 1 | 0 | 0 | 0 | 0 | 1 | 2 | 2 | 2 |
| 2017 | 0 | 4 | 4 | 1 | 1 | 3 | 0 | 2 | 0 | 0 | 2 | 2 | 2 |
| 2017 | 1 | 2 | 4 | 1 | 0 | 3 | 0 | 0 | 0 | 1 | 2 | 2 | 2 |
| 2017 | 1 | 2 | 4 | 1 | 0 | 3 | 0 | 0 | 0 | 1 | 2 | 2 | 2 |
| 2017 | 0 | 1 | 4 | 1 | 1 | 4 | 0 | 2 | 0 | 0 | 2 | 2 | 2 |
| 2017 | 1 | 1 | 4 | 1 | 1 | 0 | 0 | 0 | 0 | 1 | 2 | 2 | 2 |
| 2017 | 1 | 1 | 4 | 1 | 0 | 3 | 0 | 2 | 0 | 1 | 2 | 2 | 2 |
| 2017 | 0 | 0 | 4 | 0 | 0 | 2 | 0 | 0 | 0 | 1 | 2 | 2 | 2 |
| 2017 | 0 | 4 | 4 | 1 | 0 | 3 | 0 | 0 | 0 | 0 | 2 | 2 | 2 |
| 2017 | 0 | 3 | 4 | 1 | 0 | 2 | 0 | 0 | 0 | 1 | 2 | 2 | 2 |
| 2017 | 0 | 0 | 4 | 1 | 0 | 3 | 0 | 0 | 0 | 0 | 2 | 2 | 2 |
| 2017 | 0 | 3 | 3 | 2 | 0 | 0 | 0 | 2 | 0 | 0 | 2 | 2 | 2 |
| 2017 | 0 | 4 | 4 | 1 | 0 | 2 | 0 | 0 | 2 | 0 | 2 | 2 | 2 |
| 2017 | 1 | 2 | 4 | 1 | 0 | 4 | 0 | 0 | 0 | 0 | 2 | 2 | 2 |
| 2017 | 0 | 0 | 2 | 1 | 1 | 0 | 0 | 2 | 0 | 0 | 2 | 2 | 2 |
| 2017 | 1 | 4 | 4 | 1 | 1 | 4 | 0 | 2 | 0 | 2 | 2 | 2 | 2 |
| 2017 | 1 | 3 | 4 | 2 | 0 | 3 | 0 | 2 | 0 | 0 | 2 | 2 | 2 |
| 2017 | 1 | 3 | 3 | 1 | 0 | 0 | 0 | 0 | 0 | 1 | 2 | 2 | 2 |
| 2017 | 0 | 0 | 4 | 1 | 0 | 2 | 0 | 0 | 0 | 0 | 2 | 2 | 2 |
| 2017 | 0 | 0 | 4 | 2 | 0 | 2 | 0 | 0 | 0 | 1 | 2 | 2 | 2 |
| 2017 | 0 | 0 | 4 | 2 | 0 | 3 | 0 | 2 | 0 | 0 | 2 | 2 | 2 |
| 2017 | 0 | 2 | 1 | 0 | 1 | 1 | 0 | 2 | 2 | 1 | 2 | 2 | 2 |
| 2017 | 1 | 0 | 2 | 2 | 0 | 2 | 0 | 2 | 0 | 1 | 2 | 2 | 2 |
| 2017 | 0 | 0 | 4 | 1 | 0 | 2 | 0 | 0 | 0 | 0 | 2 | 2 | 2 |
| 2017 | 0 | 0 | 4 | 2 | 1 | 2 | 0 | 2 | 0 | 1 | 2 | 2 | 2 |
| 2017 | 0 | 0 | 1 | 1 | 0 | 2 | 0 | 2 | 0 | 0 | 2 | 2 | 2 |
| 2017 | 1 | 0 | 4 | 1 | 0 | 3 | 1 | 2 | 0 | 2 | 2 | 2 | 2 |
| 2017 | 0 | 0 | 2 | 1 | 0 | 2 | 0 | 2 | 0 | 0 | 2 | 2 | 2 |
| 2017 | 0 | 3 | 1 | 1 | 0 | 3 | 0 | 2 | 0 | 1 | 2 | 2 | 2 |
| 2017 | 1 | 4 | 2 | 2 | 0 | 2 | 0 | 2 | 0 | 1 | 2 | 2 | 2 |
| 2017 | 1 | 3 | 4 | 1 | 1 | 2 | 0 | 2 | 0 | 0 | 2 | 2 | 2 |
| 2017 | 0 | 3 | 2 | 1 | 0 | 2 | 0 | 2 | 0 | 1 | 2 | 2 | 2 |
| 2017 | 0 | 0 | 4 | 1 | 1 | 0 | 0 | 0 | 0 | 0 | 2 | 2 | 2 |
| 2017 | 1 | 0 | 1 | 2 | 0 | 2 | 0 | 2 | 0 | 1 | 2 | 2 | 2 |
| 2017 | 0 | 0 | 4 | 1 | 1 | 2 | 0 | 0 | 0 | 1 | 2 | 2 | 2 |
| 2017 | 1 | 4 | 4 | 2 | 0 | 2 | 0 | 2 | 2 | 1 | 2 | 2 | 2 |
| 2017 | 2 | 3 | 2 | 0 | 1 | 0 | 0 | 2 | 0 | 1 | 2 | 2 | 2 |
| 2017 | 0 | 4 | 4 | 1 | 1 | 4 | 0 | 0 | 2 | 2 | 2 | 2 | 2 |
| 2017 | 1 | 0 | 3 | 2 | 0 | 2 | 0 | 2 | 0 | 1 | 2 | 2 | 2 |
| 2017 | 0 | 3 | 2 | 1 | 0 | 1 | 0 | 0 | 0 | 1 | 2 | 2 | 2 |
| 2017 | 0 | 4 | 4 | 1 | 1 | 4 | 0 | 0 | 0 | 2 | 2 | 2 | 2 |
| 2017 | 0 | 0 | 0 | 1 | 0 | 3 | 0 | 0 | 0 | 0 | 2 | 2 | 2 |
| 2017 | 0 | 0 | 4 | 1 | 1 | 2 | 0 | 0 | 0 | 0 | 2 | 2 | 2 |
| 2017 | 0 | 3 | 2 | 1 | 1 | 0 | 0 | 2 | 0 | 1 | 2 | 2 | 2 |
| 2017 | 0 | 0 | 4 | 1 | 0 | 3 | 0 | 0 | 0 | 0 | 2 | 2 | 2 |
| 2017 | 0 | 0 | 4 | 1 | 0 | 2 | 0 | 0 | 0 | 1 | 2 | 2 | 2 |
| 2017 | 2 | 0 | 1 | 0 | 0 | 1 | 0 | 2 | 0 | 1 | 2 | 2 | 2 |
| 2017 | 0 | 3 | 2 | 1 | 0 | 3 | 0 | 0 | 0 | 1 | 2 | 2 | 2 |
| 2017 | 0 | 2 | 4 | 1 | 1 | 1 | 0 | 0 | 0 | 1 | 2 | 2 | 2 |
| 2017 | 1 | 0 | 1 | 1 | 0 | 3 | 0 | 0 | 0 | 1 | 2 | 2 | 2 |
| 2017 | 0 | 0 | 2 | 1 | 0 | 2 | 0 | 0 | 0 | 0 | 2 | 2 | 2 |
| 2017 | 0 | 3 | 2 | 1 | 0 | 0 | 0 | 0 | 0 | 0 | 2 | 2 | 2 |
| 2017 | 1 | 0 | 4 | 1 | 1 | 3 | 0 | 0 | 0 | 2 | 2 | 2 | 2 |
| 2017 | 0 | 0 | 0 | 1 | 1 | 2 | 0 | 2 | 0 | 1 | 2 | 2 | 2 |
| 2017 | 1 | 3 | 2 | 1 | 0 | 0 | 0 | 2 | 0 | 1 | 2 | 2 | 2 |
| 2017 | 1 | 3 | 3 | 1 | 0 | 3 | 0 | 0 | 0 | 1 | 2 | 2 | 2 |
| 2017 | 0 | 4 | 4 | 2 | 2 | 3 | 0 | 2 | 0 | 1 | 2 | 2 | 2 |
| 2017 | 1 | 0 | 4 | 1 | 0 | 3 | 0 | 0 | 0 | 1 | 2 | 2 | 2 |
| 2017 | 0 | 0 | 4 | 1 | 1 | 2 | 1 | 0 | 0 | 0 | 2 | 2 | 2 |
| 2017 | 1 | 3 | 3 | 1 | 1 | 0 | 0 | 0 | 0 | 1 | 2 | 2 | 2 |
| 2017 | 1 | 3 | 4 | 1 | 0 | 4 | 0 | 2 | 0 | 0 | 2 | 2 | 2 |
| 2017 | 0 | 0 | 4 | 1 | 0 | 0 | 0 | 2 | 0 | 1 | 2 | 2 | 2 |
| 2017 | 0 | 4 | 4 | 2 | 2 | 4 | 0 | 0 | 0 | 0 | 2 | 2 | 2 |
| 2017 | 1 | 2 | 1 | 1 | 1 | 0 | 0 | 2 | 0 | 1 | 2 | 2 | 2 |
| 2017 | 1 | 0 | 4 | 0 | 1 | 1 | 0 | 2 | 0 | 2 | 2 | 2 | 2 |
| 2017 | 0 | 0 | 0 | 1 | 2 | 2 | 0 | 0 | 0 | 0 | 2 | 2 | 2 |
| 2017 | 0 | 3 | 1 | 1 | 0 | 1 | 0 | 0 | 0 | 1 | 2 | 2 | 2 |
| 2017 | 0 | 0 | 4 | 1 | 0 | 3 | 0 | 2 | 0 | 0 | 2 | 2 | 2 |
| 2017 | 0 | 0 | 1 | 1 | 0 | 1 | 0 | 2 | 0 | 0 | 2 | 2 | 2 |
| 2017 | 1 | 4 | 4 | 1 | 0 | 4 | 0 | 0 | 0 | 0 | 2 | 2 | 2 |
| 2017 | 0 | 4 | 4 | 1 | 0 | 3 | 0 | 0 | 0 | 0 | 2 | 2 | 2 |
| 2017 | 0 | 0 | 4 | 1 | 1 | 4 | 0 | 0 | 0 | 1 | 2 | 2 | 2 |
| 2017 | 0 | 3 | 4 | 0 | 1 | 2 | 0 | 2 | 0 | 0 | 2 | 2 | 2 |
| 2017 | 0 | 3 | 0 | 1 | 0 | 0 | 0 | 0 | 0 | 0 | 2 | 2 | 2 |
| 2017 | 1 | 2 | 1 | 1 | 1 | 2 | 0 | 0 | 0 | 1 | 2 | 2 | 2 |
| 2017 | 0 | 0 | 4 | 1 | 1 | 4 | 0 | 0 | 0 | 0 | 2 | 2 | 2 |
| 2017 | 0 | 0 | 4 | 1 | 1 | 0 | 0 | 2 | 1 | 0 | 2 | 2 | 2 |
| 2017 | 0 | 2 | 0 | 1 | 1 | 1 | 0 | 2 | 0 | 1 | 2 | 2 | 2 |
| 2017 | 1 | 0 | 4 | 1 | 0 | 3 | 0 | 0 | 0 | 0 | 2 | 2 | 2 |
| 2017 | 1 | 0 | 1 | 1 | 0 | 2 | 0 | 0 | 0 | 1 | 2 | 2 | 2 |
| 2017 | 1 | 2 | 3 | 1 | 0 | 1 | 0 | 0 | 0 | 1 | 2 | 2 | 2 |
| 2017 | 1 | 0 | 4 | 1 | 0 | 2 | 0 | 0 | 0 | 0 | 2 | 2 | 2 |
| 2017 | 0 | 0 | 0 | 1 | 0 | 2 | 0 | 2 | 0 | 1 | 2 | 2 | 2 |
| 2017 | 1 | 0 | 0 | 1 | 0 | 4 | 1 | 0 | 0 | 0 | 2 | 2 | 2 |
| 2017 | 0 | 0 | 1 | 0 | 0 | 2 | 0 | 0 | 0 | 0 | 2 | 2 | 2 |
| 2017 | 1 | 0 | 4 | 1 | 2 | 2 | 0 | 2 | 0 | 2 | 2 | 2 | 2 |
| 2017 | 0 | 4 | 4 | 1 | 0 | 2 | 0 | 0 | 0 | 0 | 2 | 2 | 2 |
| 2017 | 1 | 2 | 0 | 1 | 0 | 3 | 0 | 2 | 0 | 1 | 2 | 2 | 2 |
| 2017 | 0 | 3 | 2 | 1 | 0 | 1 | 0 | 0 | 0 | 0 | 2 | 2 | 2 |
| 2017 | 0 | 0 | 4 | 1 | 0 | 3 | 0 | 0 | 0 | 0 | 2 | 2 | 2 |
| 2017 | 1 | 0 | 4 | 1 | 0 | 2 | 0 | 2 | 0 | 1 | 2 | 2 | 2 |
| 2017 | 1 | 3 | 1 | 1 | 0 | 3 | 0 | 0 | 0 | 0 | 2 | 2 | 2 |
| 2017 | 0 | 3 | 1 | 1 | 1 | 4 | 0 | 2 | 0 | 0 | 2 | 2 | 2 |
| 2017 | 0 | 0 | 1 | 1 | 1 | 1 | 0 | 2 | 0 | 1 | 2 | 2 | 2 |
| 2017 | 1 | 2 | 0 | 1 | 1 | 3 | 1 | 0 | 0 | 1 | 2 | 2 | 2 |
| 2017 | 0 | 2 | 1 | 1 | 1 | 3 | 0 | 0 | 0 | 0 | 2 | 2 | 2 |
| 2017 | 0 | 0 | 3 | 1 | 1 | 2 | 0 | 0 | 0 | 1 | 2 | 2 | 2 |
| 2017 | 1 | 0 | 4 | 1 | 0 | 2 | 0 | 0 | 0 | 1 | 2 | 2 | 2 |
| 2017 | 0 | 2 | 4 | 1 | 0 | 0 | 0 | 0 | 0 | 0 | 2 | 2 | 2 |
| 2017 | 1 | 2 | 0 | 1 | 0 | 3 | 0 | 0 | 0 | 0 | 2 | 2 | 2 |
| 2017 | 1 | 3 | 2 | 1 | 2 | 0 | 0 | 2 | 0 | 1 | 2 | 2 | 2 |
| 2017 | 0 | 0 | 2 | 1 | 0 | 2 | 0 | 2 | 0 | 1 | 2 | 2 | 2 |
| 2017 | 1 | 4 | 4 | 1 | 1 | 0 | 0 | 2 | 0 | 0 | 2 | 2 | 2 |
| 2017 | 0 | 4 | 4 | 1 | 1 | 3 | 0 | 0 | 0 | 0 | 2 | 2 | 2 |
| 2017 | 0 | 0 | 0 | 0 | 1 | 2 | 0 | 0 | 0 | 1 | 2 | 2 | 2 |
| 2017 | 1 | 2 | 4 | 1 | 0 | 0 | 0 | 0 | 1 | 0 | 2 | 2 | 2 |
| 2017 | 1 | 2 | 2 | 2 | 0 | 1 | 0 | 2 | 0 | 1 | 2 | 2 | 2 |
| 2017 | 1 | 0 | 4 | 1 | 2 | 0 | 0 | 0 | 1 | 0 | 2 | 2 | 2 |
| 2017 | 0 | 2 | 0 | 0 | 0 | 0 | 0 | 2 | 0 | 1 | 2 | 2 | 2 |
| 2017 | 0 | 3 | 2 | 1 | 0 | 3 | 0 | 0 | 0 | 0 | 2 | 2 | 2 |
| 2017 | 0 | 2 | 4 | 1 | 1 | 0 | 0 | 0 | 0 | 1 | 2 | 2 | 2 |
| 2017 | 0 | 0 | 2 | 1 | 0 | 1 | 0 | 0 | 0 | 1 | 2 | 2 | 2 |
| 2017 | 0 | 0 | 0 | 0 | 1 | 4 | 0 | 2 | 0 | 0 | 2 | 2 | 2 |
| 2017 | 0 | 2 | 4 | 1 | 1 | 0 | 0 | 0 | 1 | 1 | 2 | 2 | 2 |
| 2017 | 0 | 2 | 0 | 1 | 0 | 3 | 0 | 2 | 0 | 1 | 2 | 2 | 2 |
| 2017 | 0 | 0 | 1 | 1 | 1 | 1 | 0 | 2 | 0 | 1 | 2 | 2 | 2 |
| 2017 | 0 | 0 | 0 | 1 | 1 | 4 | 0 | 0 | 0 | 0 | 2 | 2 | 2 |
| 2017 | 0 | 3 | 1 | 1 | 1 | 0 | 0 | 0 | 0 | 1 | 2 | 2 | 2 |
| 2011 | 0 | 1 | 0 | 1 | 1 | 0 | 0 | 0 | 0 | 0 | 0 | 0 | 1 |
| 2011 | 0 | 1 | 0 | 0 | 0 | 2 | 0 | 0 | 0 | 1 | 1 | 0 | 0 |
| 2011 | 0 | 1 | 1 | 0 | 1 | 1 | 0 | 0 | 0 | 0 | 1 | 0 | 1 |
| 2011 | 0 | 1 | 0 | 0 | 1 | 2 | 0 | 1 | 0 | 1 | 0 | 1 | 1 |
| 2011 | 0 | 1 | 0 | 0 | 1 | 0 | 1 | 1 | 0 | 0 | 0 | 1 | 0 |
| 2011 | 0 | 1 | 1 | 0 | 1 | 1 | 0 | 1 | 0 | 1 | 0 | 1 | 0 |
| 2011 | 0 | 3 | 1 | 0 | 0 | 1 | 0 | 0 | 0 | 0 | 0 | 1 | 1 |
| 2011 | 1 | 3 | 1 | 0 | 0 | 2 | 0 | 0 | 0 | 1 | 0 | 1 | 1 |
| 2011 | 1 | 3 | 3 | 1 | 0 | 1 | 0 | 2 | 0 | 1 | 1 | 1 | 0 |
| 2011 | 0 | 0 | 1 | 0 | 0 | 2 | 0 | 0 | 0 | 1 | 0 | 0 | 0 |
| 2011 | 0 | 0 | 1 | 0 | 1 | 2 | 0 | 1 | 0 | 0 | 1 | 0 | 0 |
| 2011 | 0 | 1 | 0 | 0 | 0 | 2 | 1 | 2 | 0 | 0 | 0 | 1 | 0 |
| 2011 | 0 | 2 | 2 | 0 | 0 | 1 | 0 | 1 | 0 | 1 | 0 | 1 | 0 |
| 2011 | 0 | 2 | 2 | 0 | 1 | 1 | 0 | 1 | 0 | 1 | 0 | 0 | 0 |
| 2011 | 1 | 2 | 0 | 0 | 1 | 1 | 0 | 2 | 0 | 0 | 0 | 1 | 0 |
| 2011 | 1 | 2 | 0 | 0 | 1 | 0 | 0 | 0 | 0 | 0 | 0 | 1 | 0 |
| 2011 | 0 | 1 | 1 | 1 | 0 | 1 | 0 | 2 | 0 | 1 | 0 | 1 | 0 |
| 2011 | 1 | 3 | 0 | 0 | 1 | 0 | 0 | 2 | 0 | 0 | 0 | 1 | 0 |
| 2011 | 0 | 1 | 0 | 0 | 0 | 2 | 0 | 2 | 0 | 0 | 0 | 1 | 0 |
| 2011 | 1 | 0 | 1 | 0 | 1 | 0 | 0 | 0 | 0 | 2 | 0 | 0 | 0 |
| 2011 | 0 | 0 | 1 | 0 | 1 | 1 | 1 | 1 | 0 | 2 | 0 | 0 | 0 |
| 2011 | 0 | 1 | 0 | 0 | 1 | 0 | 0 | 0 | 0 | 2 | 0 | 1 | 1 |
| 2011 | 1 | 0 | 0 | 1 | 0 | 3 | 0 | 1 | 0 | 0 | 1 | 0 | 0 |
| 2011 | 0 | 1 | 1 | 0 | 0 | 3 | 0 | 0 | 0 | 2 | 0 | 0 | 0 |
| 2011 | 1 | 2 | 0 | 1 | 1 | 1 | 0 | 0 | 0 | 2 | 0 | 1 | 0 |
| 2011 | 1 | 3 | 2 | 0 | 0 | 1 | 0 | 0 | 0 | 2 | 0 | 0 | 0 |
| 2011 | 0 | 1 | 0 | 1 | 0 | 2 | 0 | 1 | 0 | 0 | 1 | 0 | 0 |
| 2011 | 1 | 1 | 0 | 1 | 0 | 4 | 0 | 0 | 0 | 0 | 0 | 1 | 0 |
| 2011 | 0 | 2 | 0 | 0 | 1 | 0 | 0 | 1 | 0 | 1 | 0 | 1 | 1 |
| 2011 | 0 | 1 | 1 | 0 | 0 | 2 | 0 | 2 | 0 | 0 | 0 | 0 | 0 |
| 2011 | 1 | 1 | 1 | 1 | 0 | 3 | 0 | 0 | 0 | 2 | 0 | 1 | 0 |
| 2011 | 1 | 2 | 2 | 0 | 0 | 1 | 0 | 2 | 0 | 2 | 0 | 1 | 1 |
| 2011 | 0 | 3 | 1 | 0 | 0 | 3 | 0 | 2 | 0 | 2 | 0 | 1 | 0 |
| 2011 | 0 | 2 | 1 | 0 | 0 | 1 | 0 | 1 | 0 | 2 | 0 | 1 | 1 |
| 2011 | 1 | 2 | 4 | 1 | 0 | 2 | 0 | 1 | 0 | 2 | 0 | 0 | 0 |
| 2011 | 0 | 0 | 0 | 0 | 1 | 0 | 0 | 2 | 0 | 0 | 0 | 0 | 0 |
| 2011 | 0 | 1 | 0 | 0 | 0 | 3 | 0 | 0 | 0 | 2 | 0 | 1 | 0 |
| 2011 | 1 | 2 | 1 | 1 | 0 | 1 | 0 | 2 | 0 | 2 | 0 | 0 | 0 |
| 2011 | 1 | 1 | 0 | 1 | 0 | 4 | 0 | 0 | 0 | 0 | 0 | 1 | 0 |
| 2011 | 0 | 0 | 0 | 1 | 0 | 1 | 0 | 0 | 0 | 2 | 0 | 1 | 0 |
| 2011 | 0 | 4 | 1 | 0 | 2 | 2 | 0 | 0 | 0 | 2 | 0 | 0 | 0 |
| 2011 | 1 | 3 | 1 | 0 | 1 | 0 | 0 | 0 | 0 | 0 | 0 | 1 | 0 |
| 2011 | 1 | 1 | 0 | 1 | 1 | 3 | 0 | 0 | 0 | 0 | 0 | 0 | 0 |
| 2011 | 0 | 1 | 0 | 0 | 1 | 1 | 0 | 2 | 0 | 0 | 0 | 1 | 0 |
| 2011 | 0 | 1 | 0 | 0 | 0 | 1 | 0 | 1 | 0 | 2 | 0 | 0 | 1 |
| 2011 | 0 | 1 | 2 | 1 | 0 | 1 | 0 | 0 | 0 | 2 | 1 | 0 | 1 |
| 2011 | 1 | 1 | 1 | 0 | 0 | 1 | 0 | 2 | 0 | 0 | 0 | 0 | 1 |
| 2011 | 0 | 0 | 1 | 0 | 0 | 2 | 0 | 2 | 0 | 2 | 1 | 0 | 0 |
| 2011 | 0 | 1 | 1 | 0 | 0 | 2 | 0 | 0 | 0 | 0 | 0 | 1 | 0 |
| 2011 | 0 | 0 | 0 | 1 | 1 | 3 | 0 | 0 | 0 | 0 | 1 | 0 | 0 |
| 2011 | 0 | 1 | 0 | 0 | 1 | 2 | 0 | 0 | 0 | 0 | 0 | 1 | 0 |
| 2011 | 1 | 1 | 1 | 1 | 0 | 1 | 0 | 1 | 0 | 1 | 0 | 1 | 0 |
| 2011 | 1 | 1 | 1 | 1 | 0 | 1 | 0 | 0 | 0 | 0 | 1 | 0 | 0 |
| 2011 | 1 | 3 | 0 | 0 | 1 | 2 | 1 | 2 | 0 | 0 | 0 | 1 | 0 |
| 2011 | 0 | 0 | 0 | 1 | 1 | 2 | 0 | 0 | 0 | 0 | 0 | 1 | 0 |
| 2011 | 1 | 3 | 2 | 0 | 0 | 0 | 0 | 0 | 0 | 2 | 0 | 1 | 0 |
| 2011 | 0 | 1 | 0 | 0 | 0 | 1 | 0 | 2 | 0 | 1 | 0 | 0 | 0 |
| 2011 | 0 | 1 | 1 | 0 | 0 | 2 | 0 | 0 | 0 | 2 | 0 | 0 | 0 |
| 2011 | 0 | 2 | 0 | 0 | 1 | 0 | 0 | 1 | 0 | 0 | 0 | 0 | 1 |
| 2011 | 1 | 0 | 1 | 0 | 0 | 2 | 0 | 1 | 0 | 1 | 0 | 1 | 0 |
| 2011 | 1 | 0 | 0 | 0 | 1 | 3 | 0 | 0 | 0 | 0 | 0 | 0 | 0 |
| 2011 | 0 | 0 | 0 | 0 | 1 | 1 | 0 | 1 | 0 | 0 | 1 | 0 | 0 |
| 2011 | 0 | 1 | 0 | 0 | 0 | 1 | 0 | 1 | 0 | 0 | 0 | 1 | 1 |
| 2011 | 1 | 2 | 0 | 1 | 0 | 2 | 0 | 0 | 0 | 0 | 1 | 0 | 0 |
| 2011 | 1 | 1 | 4 | 0 | 0 | 2 | 0 | 0 | 0 | 2 | 1 | 0 | 0 |
| 2011 | 0 | 2 | 0 | 0 | 0 | 0 | 0 | 1 | 0 | 1 | 0 | 1 | 0 |
| 2011 | 0 | 0 | 0 | 1 | 0 | 3 | 0 | 1 | 0 | 0 | 0 | 1 | 0 |
| 2011 | 0 | 0 | 1 | 0 | 1 | 3 | 0 | 2 | 0 | 2 | 1 | 0 | 0 |
| 2011 | 1 | 2 | 0 | 0 | 0 | 0 | 0 | 1 | 0 | 0 | 0 | 0 | 1 |
| 2011 | 1 | 2 | 0 | 1 | 1 | 3 | 0 | 1 | 0 | 0 | 0 | 0 | 0 |
| 2011 | 1 | 2 | 4 | 0 | 0 | 3 | 0 | 0 | 0 | 0 | 0 | 1 | 0 |
| 2011 | 1 | 1 | 0 | 1 | 0 | 1 | 0 | 0 | 0 | 1 | 1 | 0 | 0 |
| 2011 | 0 | 1 | 1 | 1 | 0 | 2 | 0 | 0 | 0 | 2 | 0 | 1 | 0 |
| 2011 | 0 | 1 | 1 | 0 | 0 | 1 | 0 | 1 | 0 | 2 | 0 | 0 | 0 |
| 2011 | 1 | 1 | 0 | 1 | 0 | 2 | 0 | 0 | 0 | 0 | 0 | 0 | 0 |
| 2011 | 1 | 2 | 0 | 0 | 1 | 0 | 0 | 0 | 0 | 0 | 0 | 0 | 0 |
| 2011 | 0 | 3 | 0 | 1 | 0 | 2 | 0 | 0 | 0 | 0 | 0 | 0 | 0 |
| 2011 | 1 | 3 | 0 | 0 | 1 | 2 | 0 | 0 | 0 | 0 | 0 | 1 | 0 |
| 2011 | 0 | 0 | 1 | 0 | 0 | 2 | 0 | 0 | 0 | 0 | 0 | 1 | 1 |
| 2011 | 1 | 1 | 1 | 0 | 0 | 1 | 0 | 2 | 0 | 0 | 1 | 0 | 0 |
| 2011 | 1 | 1 | 0 | 0 | 0 | 2 | 0 | 0 | 0 | 2 | 0 | 0 | 1 |
| 2011 | 0 | 0 | 1 | 1 | 1 | 0 | 0 | 0 | 0 | 1 | 0 | 1 | 0 |
| 2011 | 0 | 1 | 0 | 0 | 0 | 2 | 0 | 0 | 0 | 1 | 0 | 1 | 0 |
| 2011 | 0 | 0 | 1 | 0 | 0 | 1 | 0 | 0 | 0 | 2 | 0 | 1 | 0 |
| 2011 | 1 | 3 | 1 | 0 | 0 | 1 | 0 | 1 | 0 | 0 | 0 | 0 | 1 |
| 2011 | 0 | 2 | 1 | 2 | 1 | 0 | 0 | 2 | 1 | 2 | 0 | 0 | 1 |
| 2011 | 1 | 1 | 1 | 1 | 0 | 2 | 1 | 2 | 0 | 2 | 1 | 1 | 0 |
| 2011 | 0 | 0 | 1 | 1 | 0 | 2 | 0 | 1 | 0 | 0 | 1 | 1 | 0 |
| 2011 | 1 | 2 | 1 | 1 | 1 | 0 | 0 | 0 | 0 | 2 | 0 | 0 | 1 |
| 2011 | 0 | 2 | 1 | 1 | 1 | 1 | 0 | 1 | 0 | 2 | 0 | 0 | 0 |
| 2011 | 1 | 3 | 1 | 0 | 1 | 0 | 0 | 0 | 0 | 0 | 0 | 1 | 0 |
| 2011 | 0 | 0 | 1 | 1 | 0 | 3 | 0 | 0 | 0 | 0 | 0 | 0 | 1 |
| 2011 | 1 | 3 | 1 | 0 | 1 | 0 | 0 | 2 | 0 | 0 | 1 | 0 | 0 |
| 2011 | 0 | 0 | 0 | 0 | 0 | 1 | 0 | 2 | 0 | 0 | 0 | 1 | 0 |
| 2011 | 0 | 4 | 4 | 1 | 0 | 2 | 0 | 0 | 0 | 2 | 0 | 1 | 0 |
| 2011 | 0 | 3 | 4 | 0 | 2 | 3 | 0 | 2 | 0 | 2 | 0 | 1 | 0 |
| 2011 | 0 | 0 | 1 | 0 | 0 | 1 | 0 | 0 | 0 | 2 | 0 | 1 | 1 |
| 2011 | 1 | 2 | 4 | 1 | 0 | 2 | 0 | 1 | 0 | 2 | 0 | 1 | 0 |
| 2011 | 1 | 3 | 0 | 1 | 0 | 3 | 0 | 0 | 0 | 0 | 0 | 1 | 0 |
| 2011 | 0 | 1 | 0 | 0 | 0 | 3 | 0 | 0 | 0 | 0 | 0 | 1 | 1 |
| 2011 | 1 | 3 | 1 | 0 | 0 | 2 | 0 | 0 | 0 | 0 | 0 | 0 | 0 |
| 2011 | 0 | 1 | 0 | 0 | 0 | 3 | 0 | 1 | 0 | 0 | 0 | 0 | 0 |
| 2011 | 1 | 0 | 0 | 0 | 1 | 0 | 0 | 0 | 0 | 2 | 0 | 1 | 0 |
| 2011 | 1 | 0 | 0 | 0 | 1 | 2 | 0 | 0 | 0 | 0 | 0 | 1 | 0 |
| 2011 | 0 | 4 | 0 | 0 | 0 | 2 | 0 | 2 | 0 | 1 | 0 | 1 | 0 |
| 2011 | 0 | 1 | 4 | 1 | 0 | 1 | 0 | 0 | 0 | 0 | 0 | 1 | 0 |
| 2011 | 0 | 0 | 0 | 1 | 1 | 2 | 0 | 2 | 0 | 2 | 0 | 0 | 0 |
| 2011 | 0 | 1 | 1 | 0 | 0 | 2 | 0 | 0 | 0 | 0 | 0 | 0 | 0 |
| 2011 | 0 | 0 | 1 | 0 | 0 | 2 | 1 | 0 | 0 | 0 | 0 | 1 | 0 |
| 2011 | 1 | 1 | 1 | 1 | 1 | 0 | 0 | 2 | 0 | 0 | 0 | 1 | 0 |
| 2011 | 0 | 1 | 0 | 0 | 0 | 0 | 0 | 0 | 0 | 0 | 0 | 1 | 0 |
| 2011 | 1 | 3 | 1 | 0 | 0 | 1 | 0 | 0 | 0 | 0 | 0 | 1 | 0 |
| 2011 | 0 | 1 | 0 | 0 | 0 | 1 | 0 | 0 | 0 | 1 | 0 | 1 | 0 |
| 2011 | 0 | 2 | 1 | 0 | 1 | 0 | 0 | 0 | 0 | 2 | 0 | 1 | 0 |
| 2011 | 0 | 2 | 0 | 0 | 1 | 0 | 0 | 0 | 0 | 2 | 0 | 0 | 0 |
| 2011 | 0 | 3 | 0 | 0 | 1 | 3 | 0 | 0 | 0 | 2 | 0 | 1 | 0 |
| 2011 | 1 | 1 | 0 | 1 | 0 | 0 | 0 | 0 | 0 | 0 | 0 | 1 | 1 |
| 2011 | 0 | 2 | 1 | 0 | 0 | 2 | 0 | 0 | 0 | 0 | 0 | 1 | 0 |
| 2011 | 0 | 1 | 0 | 1 | 0 | 3 | 0 | 0 | 0 | 0 | 0 | 1 | 0 |
| 2011 | 1 | 1 | 1 | 1 | 0 | 3 | 0 | 0 | 0 | 2 | 0 | 1 | 0 |
| 2011 | 1 | 0 | 0 | 0 | 1 | 1 | 0 | 0 | 0 | 0 | 0 | 1 | 0 |
| 2011 | 1 | 1 | 0 | 0 | 1 | 0 | 0 | 2 | 0 | 2 | 0 | 1 | 0 |
| 2011 | 1 | 0 | 0 | 2 | 1 | 0 | 0 | 0 | 0 | 0 | 0 | 0 | 0 |
| 2011 | 0 | 0 | 0 | 0 | 0 | 3 | 0 | 0 | 0 | 0 | 0 | 1 | 0 |
| 2011 | 1 | 1 | 1 | 0 | 1 | 2 | 0 | 0 | 0 | 0 | 0 | 1 | 1 |
| 2011 | 0 | 2 | 0 | 0 | 1 | 0 | 0 | 0 | 0 | 0 | 0 | 1 | 0 |
| 2011 | 0 | 4 | 2 | 0 | 1 | 0 | 0 | 0 | 0 | 1 | 0 | 1 | 1 |
| 2011 | 0 | 1 | 0 | 1 | 0 | 3 | 0 | 0 | 0 | 1 | 0 | 1 | 0 |
| 2011 | 1 | 1 | 0 | 0 | 1 | 0 | 0 | 2 | 1 | 2 | 0 | 1 | 1 |
| 2011 | 0 | 0 | 1 | 0 | 0 | 0 | 0 | 0 | 0 | 0 | 0 | 0 | 0 |
| 2011 | 0 | 3 | 0 | 0 | 0 | 0 | 0 | 1 | 0 | 2 | 0 | 1 | 0 |
| 2011 | 0 | 3 | 1 | 1 | 1 | 0 | 0 | 2 | 0 | 0 | 0 | 0 | 0 |
| 2011 | 0 | 1 | 0 | 0 | 1 | 1 | 0 | 0 | 0 | 0 | 0 | 1 | 0 |
| 2011 | 1 | 1 | 1 | 1 | 0 | 0 | 0 | 2 | 0 | 2 | 0 | 1 | 0 |
| 2011 | 0 | 2 | 0 | 0 | 0 | 1 | 0 | 2 | 0 | 0 | 0 | 0 | 0 |
| 2011 | 1 | 3 | 1 | 1 | 1 | 0 | 0 | 2 | 0 | 1 | 0 | 1 | 0 |
| 2011 | 0 | 0 | 0 | 1 | 0 | 4 | 0 | 2 | 0 | 0 | 0 | 0 | 0 |
| 2011 | 0 | 0 | 0 | 0 | 0 | 2 | 0 | 0 | 0 | 0 | 0 | 0 | 0 |
| 2011 | 0 | 0 | 0 | 2 | 0 | 2 | 0 | 0 | 0 | 0 | 0 | 0 | 1 |
| 2011 | 0 | 1 | 0 | 1 | 0 | 1 | 0 | 0 | 0 | 1 | 1 | 0 | 0 |
| 2011 | 0 | 3 | 0 | 0 | 0 | 1 | 0 | 0 | 0 | 0 | 1 | 0 | 0 |
| 2011 | 0 | 1 | 1 | 1 | 0 | 0 | 0 | 0 | 0 | 0 | 0 | 1 | 0 |
| 2011 | 1 | 3 | 1 | 0 | 0 | 2 | 0 | 0 | 0 | 1 | 0 | 0 | 0 |
| 2011 | 1 | 0 | 0 | 0 | 0 | 1 | 0 | 2 | 0 | 0 | 0 | 1 | 0 |
| 2011 | 1 | 0 | 0 | 0 | 0 | 1 | 0 | 0 | 0 | 0 | 0 | 1 | 0 |
| 2011 | 0 | 3 | 1 | 0 | 0 | 2 | 0 | 1 | 0 | 1 | 0 | 1 | 0 |
| 2011 | 1 | 3 | 0 | 0 | 1 | 2 | 0 | 2 | 0 | 0 | 0 | 0 | 0 |
| 2011 | 1 | 1 | 0 | 0 | 0 | 2 | 0 | 0 | 0 | 0 | 0 | 0 | 0 |
| 2011 | 0 | 1 | 1 | 0 | 0 | 2 | 0 | 2 | 0 | 0 | 0 | 0 | 0 |
| 2011 | 0 | 2 | 0 | 0 | 0 | 2 | 0 | 1 | 0 | 1 | 0 | 0 | 0 |
| 2011 | 1 | 1 | 1 | 1 | 0 | 3 | 0 | 0 | 0 | 0 | 0 | 1 | 0 |
| 2011 | 1 | 0 | 1 | 0 | 1 | 0 | 0 | 2 | 0 | 0 | 0 | 1 | 0 |
| 2011 | 1 | 3 | 0 | 0 | 1 | 1 | 0 | 1 | 0 | 1 | 0 | 1 | 0 |
| 2011 | 0 | 1 | 1 | 0 | 0 | 1 | 0 | 0 | 0 | 2 | 0 | 1 | 1 |
| 2011 | 1 | 2 | 1 | 0 | 1 | 1 | 0 | 0 | 0 | 1 | 0 | 1 | 1 |
| 2011 | 0 | 4 | 0 | 1 | 0 | 0 | 0 | 2 | 0 | 0 | 0 | 1 | 0 |
| 2011 | 0 | 0 | 0 | 0 | 0 | 2 | 0 | 1 | 0 | 0 | 0 | 1 | 1 |
| 2011 | 1 | 3 | 0 | 1 | 0 | 3 | 1 | 0 | 0 | 2 | 0 | 1 | 0 |
| 2011 | 0 | 3 | 0 | 1 | 0 | 3 | 0 | 0 | 0 | 0 | 1 | 0 | 0 |
| 2011 | 0 | 2 | 2 | 1 | 0 | 1 | 1 | 2 | 0 | 2 | 0 | 0 | 0 |
| 2011 | 1 | 2 | 1 | 0 | 0 | 2 | 0 | 2 | 0 | 2 | 0 | 1 | 0 |
| 2011 | 1 | 3 | 1 | 0 | 0 | 2 | 0 | 0 | 0 | 1 | 0 | 0 | 0 |
| 2011 | 0 | 2 | 1 | 0 | 0 | 0 | 0 | 0 | 0 | 1 | 0 | 0 | 0 |
| 2011 | 0 | 2 | 2 | 1 | 0 | 0 | 0 | 0 | 0 | 0 | 0 | 0 | 1 |
| 2011 | 0 | 0 | 0 | 0 | 0 | 2 | 0 | 0 | 0 | 0 | 0 | 1 | 0 |
| 2011 | 1 | 3 | 1 | 1 | 1 | 1 | 0 | 0 | 0 | 0 | 0 | 1 | 0 |
| 2011 | 1 | 3 | 0 | 0 | 0 | 0 | 0 | 0 | 0 | 2 | 0 | 1 | 1 |
| 2011 | 0 | 4 | 0 | 0 | 0 | 2 | 0 | 0 | 0 | 2 | 0 | 0 | 0 |
| 2011 | 0 | 1 | 4 | 0 | 1 | 0 | 0 | 2 | 0 | 2 | 0 | 1 | 1 |
| 2011 | 0 | 0 | 0 | 1 | 0 | 3 | 0 | 0 | 0 | 0 | 0 | 1 | 0 |
| 2011 | 1 | 0 | 0 | 0 | 1 | 2 | 0 | 0 | 0 | 0 | 0 | 0 | 1 |
| 2011 | 0 | 1 | 0 | 0 | 1 | 1 | 0 | 0 | 0 | 2 | 0 | 1 | 1 |
| 2011 | 1 | 1 | 0 | 0 | 0 | 2 | 0 | 0 | 0 | 0 | 0 | 1 | 0 |
| 2011 | 0 | 2 | 0 | 0 | 0 | 2 | 0 | 0 | 0 | 0 | 0 | 1 | 0 |
| 2011 | 0 | 0 | 0 | 1 | 1 | 3 | 0 | 0 | 0 | 0 | 0 | 1 | 0 |
| 2011 | 0 | 3 | 1 | 0 | 0 | 1 | 0 | 2 | 0 | 2 | 0 | 0 | 0 |
| 2011 | 1 | 1 | 4 | 0 | 1 | 0 | 0 | 0 | 0 | 0 | 0 | 1 | 0 |
| 2011 | 0 | 1 | 1 | 0 | 0 | 1 | 1 | 2 | 0 | 2 | 0 | 1 | 0 |
| 2011 | 1 | 2 | 2 | 0 | 0 | 2 | 0 | 1 | 0 | 2 | 0 | 1 | 0 |
| 2011 | 1 | 2 | 2 | 1 | 0 | 1 | 0 | 0 | 0 | 2 | 0 | 0 | 0 |
| 2011 | 1 | 2 | 1 | 1 | 0 | 3 | 0 | 0 | 0 | 0 | 0 | 1 | 0 |
| 2011 | 0 | 1 | 0 | 0 | 0 | 1 | 0 | 0 | 0 | 0 | 0 | 1 | 0 |
| 2011 | 1 | 1 | 0 | 0 | 1 | 2 | 0 | 2 | 0 | 0 | 0 | 1 | 0 |
| 2011 | 0 | 1 | 0 | 0 | 1 | 0 | 0 | 2 | 0 | 0 | 0 | 1 | 1 |
| 2011 | 0 | 1 | 0 | 1 | 1 | 2 | 0 | 0 | 0 | 2 | 0 | 1 | 0 |
| 2011 | 1 | 3 | 0 | 0 | 0 | 2 | 0 | 0 | 0 | 0 | 0 | 1 | 0 |
| 2011 | 1 | 1 | 1 | 0 | 0 | 2 | 0 | 0 | 0 | 0 | 0 | 1 | 0 |
| 2011 | 0 | 1 | 1 | 0 | 0 | 0 | 0 | 1 | 0 | 0 | 0 | 0 | 0 |
| 2011 | 1 | 2 | 1 | 1 | 0 | 2 | 0 | 2 | 0 | 2 | 0 | 1 | 0 |
| 2011 | 1 | 2 | 1 | 1 | 0 | 2 | 0 | 0 | 0 | 2 | 0 | 1 | 1 |
| 2011 | 1 | 3 | 2 | 0 | 0 | 2 | 0 | 0 | 0 | 1 | 0 | 0 | 0 |
| 2011 | 0 | 2 | 2 | 0 | 0 | 0 | 0 | 2 | 0 | 2 | 0 | 1 | 0 |
| 2011 | 0 | 1 | 0 | 0 | 1 | 2 | 1 | 2 | 0 | 0 | 0 | 1 | 0 |
| 2011 | 1 | 1 | 0 | 0 | 0 | 2 | 0 | 0 | 0 | 0 | 0 | 1 | 0 |
| 2011 | 1 | 0 | 0 | 0 | 0 | 4 | 0 | 0 | 0 | 0 | 0 | 0 | 0 |
| 2011 | 0 | 1 | 1 | 1 | 0 | 2 | 0 | 2 | 0 | 2 | 0 | 1 | 0 |
| 2011 | 1 | 1 | 4 | 0 | 1 | 1 | 0 | 2 | 0 | 2 | 0 | 1 | 1 |
| 2011 | 1 | 3 | 1 | 0 | 0 | 1 | 0 | 2 | 0 | 0 | 0 | 1 | 0 |
| 2011 | 0 | 0 | 0 | 1 | 1 | 1 | 0 | 2 | 0 | 2 | 0 | 1 | 0 |
| 2011 | 0 | 2 | 1 | 1 | 0 | 1 | 0 | 1 | 0 | 0 | 0 | 0 | 0 |
| 2011 | 1 | 3 | 1 | 1 | 1 | 0 | 0 | 2 | 0 | 2 | 0 | 0 | 1 |
| 2011 | 0 | 0 | 0 | 1 | 0 | 2 | 0 | 2 | 0 | 1 | 0 | 1 | 0 |
| 2011 | 1 | 3 | 4 | 0 | 0 | 0 | 0 | 0 | 0 | 2 | 0 | 1 | 0 |
| 2011 | 1 | 3 | 1 | 1 | 1 | 0 | 0 | 0 | 0 | 1 | 0 | 1 | 1 |
| 2011 | 0 | 0 | 0 | 0 | 1 | 0 | 0 | 0 | 0 | 0 | 0 | 1 | 1 |
| 2011 | 0 | 2 | 0 | 1 | 1 | 2 | 0 | 2 | 0 | 1 | 0 | 1 | 0 |
| 2011 | 0 | 1 | 0 | 1 | 0 | 2 | 0 | 0 | 0 | 1 | 0 | 0 | 0 |
| 2011 | 1 | 1 | 1 | 0 | 1 | 1 | 0 | 0 | 0 | 1 | 0 | 1 | 0 |
| 2011 | 0 | 1 | 1 | 0 | 0 | 3 | 0 | 0 | 0 | 1 | 0 | 1 | 0 |
| 2011 | 0 | 1 | 0 | 1 | 1 | 2 | 0 | 2 | 0 | 0 | 1 | 0 | 1 |
| 2011 | 1 | 1 | 0 | 0 | 0 | 2 | 1 | 0 | 0 | 0 | 0 | 1 | 1 |
| 2011 | 0 | 0 | 0 | 0 | 0 | 2 | 0 | 0 | 0 | 0 | 0 | 1 | 0 |
| 2011 | 0 | 1 | 0 | 0 | 1 | 2 | 0 | 2 | 0 | 0 | 0 | 0 | 1 |
| 2011 | 0 | 3 | 0 | 1 | 1 | 0 | 0 | 2 | 1 | 1 | 0 | 0 | 0 |
| 2011 | 1 | 3 | 0 | 0 | 1 | 0 | 0 | 1 | 1 | 1 | 0 | 0 | 1 |
| 2011 | 1 | 0 | 0 | 0 | 0 | 2 | 0 | 2 | 0 | 0 | 0 | 1 | 0 |
| 2011 | 1 | 0 | 1 | 0 | 0 | 1 | 0 | 2 | 0 | 1 | 0 | 0 | 0 |
| 2011 | 0 | 1 | 1 | 0 | 0 | 1 | 0 | 2 | 0 | 1 | 0 | 0 | 0 |
| 2011 | 0 | 2 | 1 | 0 | 1 | 2 | 0 | 0 | 0 | 0 | 0 | 0 | 0 |
| 2011 | 1 | 0 | 1 | 0 | 0 | 3 | 0 | 0 | 0 | 1 | 0 | 0 | 1 |
| 2011 | 0 | 1 | 4 | 0 | 0 | 2 | 0 | 2 | 0 | 1 | 0 | 1 | 0 |
| 2011 | 0 | 1 | 0 | 1 | 1 | 2 | 1 | 2 | 0 | 1 | 0 | 1 | 0 |
| 2011 | 0 | 0 | 0 | 2 | 1 | 0 | 1 | 0 | 0 | 2 | 0 | 1 | 0 |
| 2011 | 0 | 1 | 0 | 0 | 1 | 2 | 0 | 0 | 0 | 0 | 0 | 1 | 0 |
| 2011 | 0 | 3 | 0 | 0 | 1 | 0 | 0 | 2 | 0 | 0 | 0 | 1 | 0 |
| 2011 | 0 | 1 | 0 | 0 | 1 | 2 | 0 | 2 | 0 | 1 | 0 | 1 | 0 |
| 2011 | 0 | 0 | 0 | 1 | 1 | 0 | 0 | 2 | 1 | 0 | 0 | 1 | 0 |
| 2011 | 1 | 1 | 1 | 0 | 0 | 2 | 0 | 2 | 0 | 1 | 0 | 1 | 1 |
| 2011 | 1 | 3 | 1 | 0 | 0 | 2 | 0 | 0 | 0 | 0 | 0 | 0 | 1 |
| 2011 | 0 | 2 | 1 | 0 | 1 | 0 | 0 | 1 | 0 | 0 | 0 | 0 | 0 |
| 2011 | 0 | 3 | 1 | 1 | 1 | 0 | 0 | 1 | 0 | 1 | 0 | 0 | 0 |
| 2011 | 1 | 2 | 1 | 1 | 0 | 0 | 0 | 2 | 0 | 1 | 0 | 0 | 1 |
| 2011 | 0 | 1 | 0 | 0 | 1 | 2 | 0 | 2 | 0 | 1 | 0 | 1 | 0 |
| 2011 | 0 | 1 | 0 | 1 | 0 | 1 | 0 | 2 | 0 | 0 | 0 | 1 | 0 |
| 2011 | 1 | 3 | 1 | 1 | 0 | 3 | 0 | 2 | 0 | 0 | 0 | 0 | 0 |
| 2011 | 0 | 1 | 0 | 2 | 0 | 0 | 0 | 2 | 0 | 0 | 0 | 0 | 0 |
| 2011 | 1 | 3 | 1 | 0 | 1 | 0 | 0 | 0 | 1 | 1 | 0 | 0 | 1 |
| 2011 | 0 | 0 | 0 | 1 | 0 | 1 | 0 | 2 | 0 | 1 | 0 | 1 | 0 |
| 2011 | 1 | 1 | 0 | 0 | 0 | 1 | 0 | 0 | 0 | 0 | 0 | 0 | 0 |
| 2011 | 1 | 1 | 4 | 1 | 0 | 2 | 0 | 2 | 0 | 0 | 0 | 0 | 1 |
| 2011 | 0 | 1 | 0 | 1 | 1 | 2 | 0 | 1 | 0 | 1 | 0 | 1 | 0 |
| 2011 | 0 | 1 | 0 | 1 | 0 | 2 | 0 | 2 | 0 | 0 | 0 | 0 | 0 |
| 2011 | 1 | 1 | 1 | 1 | 0 | 3 | 0 | 2 | 0 | 0 | 0 | 1 | 0 |
| 2011 | 0 | 3 | 1 | 0 | 1 | 0 | 0 | 2 | 0 | 1 | 0 | 1 | 0 |
| 2011 | 0 | 2 | 0 | 0 | 1 | 2 | 0 | 0 | 0 | 0 | 0 | 0 | 1 |
| 2011 | 0 | 2 | 2 | 0 | 0 | 0 | 0 | 2 | 0 | 1 | 0 | 0 | 1 |
| 2011 | 1 | 1 | 0 | 1 | 0 | 3 | 0 | 2 | 0 | 1 | 0 | 1 | 0 |
| 2011 | 0 | 0 | 0 | 0 | 1 | 2 | 0 | 0 | 0 | 0 | 0 | 1 | 0 |
| 2011 | 1 | 4 | 1 | 1 | 1 | 2 | 0 | 2 | 0 | 1 | 0 | 0 | 0 |
| 2011 | 1 | 3 | 0 | 0 | 1 | 0 | 0 | 0 | 1 | 0 | 0 | 1 | 1 |
| 2011 | 1 | 1 | 0 | 0 | 1 | 1 | 0 | 0 | 0 | 1 | 1 | 0 | 0 |
| 2011 | 0 | 1 | 0 | 0 | 0 | 1 | 1 | 0 | 0 | 0 | 0 | 0 | 0 |
| 2011 | 1 | 1 | 2 | 0 | 0 | 2 | 0 | 1 | 0 | 1 | 0 | 0 | 0 |
| 2011 | 1 | 2 | 2 | 0 | 0 | 2 | 0 | 0 | 0 | 1 | 0 | 0 | 0 |
| 2011 | 0 | 3 | 1 | 0 | 1 | 0 | 0 | 0 | 0 | 0 | 0 | 1 | 0 |
| 2011 | 0 | 0 | 2 | 0 | 0 | 0 | 0 | 0 | 0 | 1 | 0 | 1 | 0 |
| 2011 | 1 | 4 | 1 | 2 | 0 | 1 | 0 | 2 | 0 | 0 | 0 | 0 | 0 |
| 2011 | 1 | 2 | 0 | 1 | 0 | 3 | 0 | 2 | 0 | 0 | 0 | 0 | 0 |
| 2011 | 0 | 1 | 4 | 0 | 2 | 5 | 0 | 0 | 0 | 0 | 0 | 1 | 0 |
| 2011 | 0 | 1 | 1 | 0 | 0 | 3 | 0 | 2 | 0 | 0 | 0 | 1 | 0 |
| 2011 | 0 | 1 | 1 | 0 | 0 | 2 | 0 | 2 | 0 | 1 | 0 | 0 | 0 |
| 2011 | 0 | 0 | 1 | 1 | 0 | 4 | 0 | 0 | 0 | 0 | 0 | 0 | 1 |
| 2011 | 0 | 0 | 1 | 1 | 0 | 2 | 0 | 1 | 0 | 1 | 0 | 0 | 0 |
| 2011 | 1 | 1 | 2 | 1 | 0 | 3 | 1 | 2 | 0 | 0 | 0 | 1 | 0 |
| 2011 | 0 | 1 | 0 | 1 | 0 | 2 | 0 | 2 | 0 | 0 | 0 | 1 | 0 |
| 2011 | 1 | 0 | 0 | 1 | 0 | 2 | 1 | 2 | 0 | 0 | 1 | 1 | 0 |
| 2011 | 0 | 1 | 0 | 0 | 0 | 2 | 0 | 2 | 0 | 0 | 0 | 1 | 0 |
| 2011 | 1 | 3 | 2 | 0 | 0 | 1 | 0 | 0 | 0 | 1 | 0 | 1 | 1 |
| 2011 | 0 | 1 | 0 | 0 | 0 | 2 | 0 | 2 | 0 | 1 | 0 | 1 | 0 |
| 2011 | 0 | 0 | 2 | 0 | 0 | 2 | 0 | 2 | 0 | 0 | 0 | 1 | 0 |
| 2011 | 1 | 1 | 0 | 0 | 0 | 0 | 0 | 0 | 0 | 1 | 0 | 1 | 0 |
| 2011 | 0 | 2 | 1 | 0 | 0 | 2 | 0 | 2 | 0 | 1 | 0 | 0 | 0 |
| 2011 | 0 | 1 | 4 | 0 | 0 | 1 | 0 | 2 | 0 | 0 | 0 | 1 | 0 |
| 2011 | 0 | 1 | 0 | 1 | 0 | 0 | 0 | 0 | 0 | 0 | 0 | 1 | 0 |
| 2011 | 0 | 3 | 1 | 0 | 1 | 0 | 0 | 2 | 0 | 1 | 0 | 1 | 0 |
| 2011 | 0 | 1 | 0 | 0 | 0 | 1 | 0 | 0 | 0 | 1 | 0 | 1 | 1 |
| 2011 | 1 | 1 | 0 | 0 | 0 | 0 | 0 | 0 | 0 | 1 | 0 | 0 | 0 |
| 2011 | 1 | 1 | 1 | 0 | 0 | 0 | 0 | 2 | 0 | 0 | 0 | 0 | 0 |
| 2011 | 0 | 2 | 0 | 0 | 1 | 2 | 0 | 2 | 0 | 1 | 0 | 1 | 1 |
| 2011 | 1 | 1 | 0 | 1 | 0 | 4 | 0 | 2 | 2 | 0 | 0 | 0 | 0 |
| 2011 | 0 | 1 | 0 | 1 | 1 | 0 | 0 | 0 | 0 | 1 | 0 | 0 | 1 |
| 2011 | 0 | 2 | 0 | 0 | 1 | 0 | 0 | 2 | 1 | 1 | 0 | 0 | 1 |
| 2011 | 1 | 3 | 1 | 1 | 0 | 0 | 0 | 2 | 0 | 1 | 0 | 1 | 0 |
| 2011 | 0 | 3 | 0 | 0 | 1 | 2 | 0 | 2 | 0 | 0 | 0 | 1 | 0 |
| 2011 | 1 | 1 | 1 | 0 | 0 | 0 | 0 | 0 | 0 | 0 | 0 | 0 | 0 |
| 2011 | 1 | 2 | 1 | 1 | 0 | 3 | 0 | 2 | 0 | 1 | 0 | 1 | 0 |
| 2011 | 1 | 3 | 0 | 0 | 1 | 0 | 0 | 2 | 0 | 0 | 0 | 0 | 0 |
| 2011 | 0 | 1 | 0 | 1 | 1 | 1 | 0 | 0 | 0 | 0 | 0 | 1 | 0 |
| 2011 | 0 | 4 | 0 | 0 | 0 | 1 | 0 | 2 | 0 | 0 | 0 | 1 | 0 |
| 2011 | 0 | 0 | 0 | 1 | 0 | 2 | 0 | 0 | 0 | 0 | 0 | 1 | 1 |
| 2011 | 0 | 3 | 0 | 0 | 1 | 0 | 0 | 0 | 0 | 0 | 0 | 0 | 0 |
| 2011 | 1 | 0 | 0 | 0 | 1 | 0 | 0 | 1 | 1 | 1 | 0 | 0 | 0 |
| 2011 | 0 | 0 | 0 | 1 | 0 | 4 | 0 | 0 | 0 | 0 | 0 | 1 | 0 |
| 2011 | 0 | 1 | 1 | 0 | 1 | 2 | 0 | 2 | 0 | 1 | 0 | 0 | 0 |
| 2011 | 0 | 4 | 1 | 1 | 0 | 2 | 1 | 0 | 0 | 0 | 0 | 1 | 0 |
| 2011 | 0 | 1 | 1 | 0 | 1 | 1 | 0 | 0 | 2 | 1 | 0 | 1 | 1 |
| 2011 | 1 | 3 | 1 | 1 | 0 | 3 | 0 | 0 | 0 | 0 | 0 | 0 | 0 |
| 2011 | 0 | 0 | 2 | 1 | 0 | 4 | 0 | 2 | 0 | 0 | 0 | 0 | 0 |
| 2011 | 1 | 3 | 3 | 1 | 0 | 4 | 0 | 2 | 0 | 0 | 0 | 0 | 0 |
| 2011 | 0 | 1 | 1 | 0 | 1 | 2 | 0 | 0 | 0 | 1 | 0 | 1 | 0 |
| 2011 | 1 | 1 | 1 | 1 | 0 | 1 | 0 | 0 | 0 | 0 | 0 | 1 | 0 |
| 2011 | 1 | 0 | 1 | 0 | 0 | 1 | 0 | 2 | 0 | 1 | 0 | 1 | 0 |
| 2011 | 0 | 1 | 1 | 1 | 1 | 4 | 0 | 0 | 0 | 0 | 0 | 1 | 0 |
| 2011 | 1 | 0 | 1 | 1 | 0 | 4 | 0 | 2 | 0 | 0 | 0 | 0 | 0 |
| 2011 | 0 | 1 | 1 | 0 | 0 | 2 | 0 | 2 | 0 | 0 | 0 | 0 | 0 |
| 2011 | 1 | 3 | 1 | 0 | 0 | 2 | 0 | 2 | 0 | 1 | 0 | 0 | 0 |
| 2011 | 0 | 1 | 2 | 0 | 1 | 3 | 0 | 0 | 0 | 0 | 0 | 0 | 1 |
| 2011 | 0 | 1 | 0 | 0 | 0 | 2 | 0 | 2 | 0 | 0 | 0 | 1 | 0 |
| 2011 | 0 | 0 | 1 | 0 | 1 | 1 | 0 | 0 | 0 | 0 | 0 | 0 | 0 |
| 2011 | 0 | 3 | 0 | 0 | 1 | 0 | 0 | 2 | 1 | 1 | 0 | 1 | 1 |
| 2011 | 1 | 1 | 0 | 1 | 1 | 2 | 0 | 2 | 0 | 0 | 0 | 1 | 0 |
| 2011 | 0 | 4 | 0 | 1 | 0 | 2 | 0 | 2 | 0 | 0 | 0 | 1 | 0 |
| 2011 | 0 | 0 | 0 | 1 | 0 | 4 | 1 | 0 | 0 | 0 | 0 | 0 | 1 |
| 2011 | 0 | 0 | 0 | 0 | 0 | 2 | 0 | 2 | 0 | 1 | 0 | 1 | 0 |
| 2011 | 1 | 2 | 0 | 0 | 0 | 2 | 0 | 2 | 0 | 1 | 0 | 1 | 0 |
| 2011 | 0 | 1 | 0 | 1 | 0 | 3 | 0 | 2 | 0 | 0 | 0 | 1 | 0 |
| 2011 | 0 | 2 | 0 | 1 | 0 | 0 | 0 | 0 | 0 | 0 | 0 | 0 | 1 |
| 2011 | 1 | 1 | 1 | 1 | 0 | 2 | 1 | 1 | 0 | 1 | 0 | 0 | 0 |
| 2011 | 0 | 0 | 0 | 0 | 0 | 1 | 0 | 2 | 0 | 1 | 0 | 1 | 0 |
| 2011 | 0 | 3 | 0 | 1 | 0 | 3 | 0 | 2 | 0 | 1 | 0 | 0 | 0 |
| 2011 | 0 | 4 | 0 | 1 | 0 | 4 | 0 | 0 | 0 | 0 | 0 | 0 | 1 |
| 2011 | 1 | 3 | 3 | 1 | 0 | 1 | 0 | 2 | 0 | 2 | 0 | 0 | 0 |
| 2011 | 1 | 3 | 1 | 0 | 1 | 2 | 0 | 2 | 0 | 1 | 0 | 1 | 1 |
| 2011 | 1 | 4 | 0 | 0 | 1 | 0 | 1 | 2 | 0 | 0 | 0 | 1 | 1 |
| 2011 | 0 | 1 | 1 | 0 | 0 | 1 | 0 | 2 | 0 | 1 | 0 | 1 | 0 |
| 2011 | 0 | 1 | 0 | 0 | 1 | 0 | 0 | 0 | 0 | 0 | 0 | 1 | 0 |
| 2011 | 0 | 2 | 0 | 0 | 0 | 0 | 0 | 2 | 0 | 1 | 0 | 1 | 0 |
| 2011 | 0 | 1 | 1 | 1 | 0 | 1 | 0 | 2 | 0 | 0 | 0 | 0 | 0 |
| 2011 | 0 | 3 | 0 | 1 | 0 | 0 | 0 | 0 | 1 | 0 | 0 | 1 | 0 |
| 2011 | 1 | 2 | 0 | 1 | 1 | 4 | 0 | 0 | 0 | 0 | 0 | 0 | 1 |
| 2011 | 1 | 1 | 0 | 0 | 2 | 2 | 0 | 2 | 0 | 0 | 0 | 0 | 1 |
| 2011 | 1 | 3 | 0 | 1 | 1 | 1 | 0 | 2 | 0 | 0 | 0 | 1 | 1 |
| 2011 | 0 | 3 | 3 | 1 | 0 | 0 | 0 | 2 | 0 | 1 | 0 | 0 | 0 |
| 2011 | 1 | 4 | 4 | 1 | 1 | 3 | 0 | 0 | 0 | 0 | 0 | 0 | 0 |
| 2011 | 0 | 1 | 1 | 0 | 0 | 2 | 0 | 2 | 0 | 1 | 0 | 1 | 0 |
| 2011 | 0 | 1 | 1 | 1 | 0 | 3 | 0 | 2 | 0 | 0 | 0 | 0 | 0 |
| 2011 | 1 | 1 | 1 | 2 | 1 | 2 | 0 | 2 | 0 | 1 | 0 | 0 | 0 |
| 2011 | 0 | 2 | 0 | 1 | 1 | 0 | 0 | 2 | 0 | 1 | 1 | 0 | 0 |
| 2011 | 1 | 4 | 0 | 1 | 1 | 2 | 0 | 0 | 0 | 0 | 0 | 0 | 1 |
| 2011 | 0 | 0 | 0 | 0 | 0 | 5 | 0 | 0 | 0 | 0 | 0 | 0 | 1 |
| 2011 | 0 | 3 | 1 | 1 | 0 | 2 | 0 | 2 | 0 | 1 | 0 | 0 | 0 |
| 2011 | 0 | 3 | 0 | 0 | 1 | 0 | 0 | 0 | 0 | 0 | 0 | 0 | 0 |
| 2011 | 1 | 3 | 0 | 1 | 0 | 3 | 0 | 0 | 0 | 0 | 0 | 0 | 0 |
| 2011 | 1 | 3 | 2 | 0 | 0 | 3 | 0 | 1 | 0 | 0 | 0 | 0 | 0 |
| 2011 | 1 | 0 | 0 | 1 | 0 | 3 | 0 | 2 | 0 | 0 | 0 | 1 | 0 |
| 2011 | 1 | 0 | 0 | 2 | 0 | 0 | 0 | 1 | 0 | 1 | 0 | 0 | 0 |
| 2011 | 1 | 1 | 1 | 0 | 0 | 1 | 1 | 0 | 0 | 1 | 0 | 1 | 0 |
| 2011 | 0 | 2 | 0 | 0 | 0 | 2 | 0 | 2 | 0 | 1 | 0 | 1 | 0 |
| 2011 | 1 | 0 | 1 | 0 | 0 | 2 | 0 | 1 | 0 | 1 | 0 | 0 | 0 |
| 2011 | 1 | 3 | 1 | 0 | 0 | 2 | 0 | 2 | 0 | 1 | 0 | 1 | 1 |
| 2011 | 0 | 2 | 1 | 0 | 1 | 0 | 0 | 2 | 0 | 0 | 0 | 0 | 0 |
| 2011 | 0 | 0 | 1 | 1 | 0 | 2 | 0 | 0 | 0 | 1 | 0 | 0 | 0 |
| 2011 | 0 | 4 | 1 | 0 | 1 | 2 | 0 | 2 | 0 | 1 | 0 | 0 | 0 |
| 2011 | 0 | 0 | 0 | 1 | 0 | 2 | 0 | 2 | 0 | 0 | 0 | 1 | 0 |
| 2011 | 0 | 1 | 1 | 1 | 0 | 1 | 0 | 2 | 0 | 0 | 0 | 0 | 0 |
| 2011 | 1 | 3 | 1 | 0 | 0 | 2 | 0 | 2 | 0 | 0 | 0 | 1 | 0 |
| 2011 | 0 | 3 | 4 | 2 | 0 | 3 | 0 | 0 | 0 | 1 | 0 | 0 | 0 |
| 2011 | 0 | 3 | 3 | 1 | 0 | 1 | 0 | 2 | 0 | 0 | 0 | 0 | 0 |
| 2011 | 0 | 3 | 0 | 0 | 0 | 0 | 0 | 2 | 0 | 0 | 0 | 1 | 0 |
| 2011 | 0 | 1 | 0 | 0 | 0 | 2 | 0 | 2 | 0 | 1 | 0 | 0 | 0 |
| 2011 | 1 | 1 | 1 | 1 | 0 | 3 | 0 | 0 | 0 | 1 | 0 | 1 | 0 |
| 2011 | 0 | 0 | 1 | 1 | 0 | 3 | 0 | 0 | 0 | 0 | 0 | 1 | 0 |
| 2011 | 1 | 1 | 1 | 0 | 1 | 2 | 0 | 0 | 0 | 1 | 0 | 0 | 1 |
| 2011 | 0 | 2 | 2 | 1 | 0 | 0 | 0 | 0 | 0 | 1 | 0 | 0 | 1 |
| 2011 | 0 | 0 | 0 | 1 | 0 | 3 | 0 | 0 | 0 | 0 | 0 | 1 | 1 |
| 2011 | 0 | 2 | 1 | 0 | 0 | 0 | 0 | 2 | 0 | 0 | 0 | 1 | 0 |
| 2011 | 1 | 4 | 1 | 1 | 0 | 2 | 0 | 2 | 0 | 0 | 0 | 1 | 1 |
| 2011 | 0 | 3 | 2 | 0 | 0 | 2 | 0 | 0 | 0 | 0 | 0 | 1 | 0 |
| 2011 | 1 | 1 | 0 | 0 | 1 | 1 | 0 | 2 | 0 | 1 | 0 | 1 | 0 |
| 2011 | 1 | 0 | 0 | 0 | 0 | 3 | 1 | 0 | 0 | 0 | 0 | 1 | 1 |
| 2011 | 1 | 1 | 1 | 1 | 0 | 0 | 0 | 2 | 0 | 1 | 2 | 2 | 2 |
| 2011 | 1 | 4 | 0 | 1 | 1 | 0 | 0 | 2 | 1 | 0 | 0 | 1 | 1 |
| 2011 | 0 | 2 | 0 | 0 | 0 | 1 | 0 | 1 | 0 | 1 | 0 | 1 | 0 |
| 2011 | 1 | 4 | 1 | 0 | 0 | 2 | 0 | 0 | 0 | 1 | 0 | 0 | 0 |
| 2011 | 1 | 1 | 2 | 0 | 1 | 0 | 0 | 0 | 1 | 0 | 0 | 1 | 0 |
| 2011 | 0 | 0 | 0 | 1 | 0 | 2 | 0 | 1 | 0 | 0 | 0 | 1 | 1 |
| 2011 | 1 | 3 | 2 | 0 | 0 | 2 | 0 | 0 | 0 | 0 | 0 | 0 | 1 |
| 2011 | 1 | 1 | 0 | 0 | 1 | 0 | 0 | 2 | 1 | 0 | 0 | 1 | 0 |
| 2011 | 1 | 1 | 1 | 0 | 0 | 2 | 0 | 2 | 0 | 0 | 0 | 0 | 0 |
| 2011 | 1 | 0 | 0 | 1 | 0 | 3 | 0 | 0 | 0 | 0 | 0 | 1 | 0 |
| 2011 | 1 | 1 | 1 | 1 | 0 | 1 | 0 | 2 | 0 | 1 | 0 | 1 | 0 |
| 2011 | 1 | 1 | 1 | 0 | 0 | 2 | 0 | 2 | 0 | 0 | 0 | 1 | 0 |
| 2011 | 1 | 2 | 0 | 1 | 0 | 4 | 0 | 0 | 0 | 0 | 0 | 0 | 0 |
| 2011 | 1 | 2 | 2 | 1 | 0 | 1 | 0 | 2 | 0 | 1 | 0 | 0 | 0 |
| 2011 | 0 | 1 | 0 | 1 | 1 | 2 | 0 | 2 | 0 | 0 | 0 | 1 | 0 |
| 2011 | 1 | 1 | 0 | 0 | 0 | 2 | 0 | 2 | 0 | 0 | 0 | 1 | 0 |
| 2011 | 1 | 3 | 3 | 1 | 0 | 1 | 0 | 0 | 0 | 1 | 0 | 0 | 1 |
| 2011 | 1 | 3 | 1 | 0 | 0 | 3 | 0 | 0 | 0 | 0 | 0 | 1 | 0 |
| 2011 | 0 | 2 | 1 | 1 | 0 | 2 | 1 | 0 | 0 | 0 | 0 | 0 | 0 |
| 2011 | 1 | 1 | 0 | 1 | 0 | 4 | 0 | 0 | 0 | 0 | 0 | 1 | 0 |
| 2011 | 0 | 1 | 1 | 2 | 0 | 1 | 0 | 2 | 0 | 0 | 0 | 1 | 1 |
| 2011 | 1 | 1 | 1 | 1 | 0 | 1 | 0 | 0 | 0 | 1 | 0 | 1 | 0 |
| 2011 | 0 | 4 | 2 | 1 | 0 | 1 | 0 | 0 | 0 | 0 | 0 | 0 | 0 |
| 2011 | 0 | 1 | 1 | 1 | 0 | 1 | 0 | 0 | 0 | 1 | 0 | 1 | 0 |
| 2011 | 0 | 2 | 0 | 0 | 1 | 0 | 0 | 0 | 0 | 0 | 0 | 0 | 1 |
| 2011 | 1 | 3 | 0 | 1 | 1 | 3 | 0 | 1 | 0 | 0 | 0 | 1 | 0 |
| 2011 | 0 | 1 | 1 | 0 | 0 | 0 | 0 | 0 | 0 | 1 | 0 | 0 | 0 |
| 2011 | 1 | 4 | 0 | 0 | 0 | 3 | 0 | 0 | 0 | 0 | 0 | 1 | 1 |
| 2011 | 0 | 2 | 0 | 0 | 0 | 3 | 0 | 2 | 0 | 0 | 0 | 1 | 0 |
| 2011 | 0 | 1 | 2 | 0 | 0 | 1 | 0 | 0 | 0 | 1 | 0 | 0 | 1 |
| 2011 | 1 | 0 | 1 | 0 | 1 | 2 | 0 | 2 | 1 | 1 | 0 | 0 | 0 |
| 2011 | 0 | 0 | 0 | 0 | 0 | 1 | 0 | 2 | 0 | 0 | 0 | 1 | 0 |
| 2011 | 1 | 2 | 0 | 0 | 1 | 0 | 0 | 2 | 0 | 0 | 0 | 0 | 0 |
| 2011 | 0 | 1 | 0 | 1 | 0 | 3 | 0 | 0 | 0 | 0 | 0 | 0 | 0 |
| 2011 | 1 | 1 | 0 | 0 | 1 | 3 | 0 | 0 | 0 | 0 | 0 | 1 | 0 |
| 2011 | 1 | 3 | 4 | 1 | 0 | 4 | 1 | 0 | 0 | 0 | 0 | 1 | 0 |
| 2011 | 0 | 1 | 1 | 2 | 0 | 4 | 0 | 2 | 0 | 0 | 0 | 0 | 0 |
| 2011 | 1 | 1 | 0 | 1 | 0 | 4 | 0 | 2 | 0 | 0 | 0 | 0 | 0 |
| 2011 | 0 | 1 | 0 | 1 | 0 | 2 | 0 | 0 | 0 | 0 | 0 | 0 | 1 |
| 2011 | 0 | 2 | 1 | 0 | 1 | 0 | 0 | 2 | 0 | 1 | 0 | 0 | 0 |
| 2011 | 1 | 1 | 0 | 0 | 0 | 2 | 0 | 2 | 0 | 0 | 0 | 1 | 1 |
| 2011 | 0 | 1 | 1 | 1 | 0 | 2 | 0 | 2 | 0 | 0 | 0 | 1 | 0 |
| 2011 | 0 | 1 | 0 | 1 | 0 | 1 | 0 | 1 | 0 | 1 | 0 | 0 | 0 |
| 2011 | 0 | 0 | 0 | 0 | 0 | 2 | 0 | 2 | 0 | 0 | 0 | 1 | 0 |
| 2011 | 0 | 2 | 1 | 1 | 0 | 3 | 0 | 2 | 0 | 0 | 0 | 1 | 0 |
| 2011 | 1 | 0 | 1 | 0 | 0 | 0 | 0 | 2 | 0 | 0 | 0 | 0 | 0 |
| 2011 | 0 | 1 | 1 | 2 | 0 | 1 | 0 | 2 | 0 | 0 | 0 | 0 | 0 |
| 2011 | 1 | 0 | 1 | 2 | 0 | 2 | 1 | 0 | 0 | 1 | 0 | 0 | 0 |
| 2011 | 1 | 0 | 1 | 2 | 0 | 1 | 0 | 2 | 0 | 1 | 0 | 0 | 0 |
| 2011 | 0 | 0 | 0 | 1 | 0 | 2 | 0 | 2 | 0 | 0 | 0 | 1 | 0 |
| 2011 | 1 | 2 | 1 | 0 | 1 | 2 | 0 | 0 | 0 | 0 | 0 | 0 | 1 |
| 2011 | 1 | 3 | 0 | 0 | 0 | 2 | 0 | 1 | 0 | 0 | 0 | 0 | 0 |
| 2011 | 0 | 1 | 1 | 1 | 0 | 1 | 0 | 1 | 0 | 1 | 0 | 0 | 1 |
| 2012 | 1 | 0 | 1 | 2 | 0 | 2 | 0 | 0 | 0 | 1 | 0 | 2 | 0 |
| 2012 | 0 | 2 | 2 | 0 | 0 | 2 | 0 | 0 | 0 | 1 | 0 | 2 | 0 |
| 2012 | 1 | 1 | 0 | 0 | 0 | 3 | 1 | 0 | 0 | 0 | 0 | 2 | 0 |
| 2012 | 0 | 0 | 0 | 0 | 0 | 1 | 0 | 0 | 0 | 0 | 0 | 2 | 1 |
| 2012 | 0 | 3 | 4 | 0 | 0 | 3 | 1 | 0 | 0 | 0 | 0 | 1 | 0 |
| 2012 | 0 | 1 | 2 | 1 | 0 | 2 | 0 | 1 | 0 | 1 | 0 | 2 | 1 |
| 2012 | 1 | 1 | 2 | 1 | 0 | 1 | 0 | 0 | 0 | 1 | 0 | 2 | 0 |
| 2012 | 0 | 4 | 0 | 0 | 0 | 2 | 0 | 1 | 0 | 0 | 0 | 2 | 0 |
| 2012 | 1 | 2 | 1 | 1 | 1 | 2 | 0 | 2 | 0 | 1 | 0 | 2 | 0 |
| 2012 | 1 | 3 | 1 | 1 | 0 | 0 | 0 | 0 | 0 | 1 | 0 | 2 | 1 |
| 2012 | 0 | 3 | 0 | 0 | 0 | 2 | 0 | 0 | 0 | 0 | 0 | 2 | 0 |
| 2012 | 1 | 3 | 0 | 2 | 1 | 1 | 0 | 2 | 0 | 1 | 0 | 2 | 1 |
| 2012 | 0 | 3 | 0 | 1 | 0 | 2 | 0 | 0 | 0 | 0 | 0 | 2 | 0 |
| 2012 | 1 | 4 | 1 | 1 | 1 | 0 | 0 | 0 | 0 | 0 | 0 | 1 | 0 |
| 2012 | 0 | 4 | 4 | 0 | 0 | 2 | 0 | 1 | 0 | 0 | 0 | 2 | 0 |
| 2012 | 0 | 0 | 2 | 0 | 0 | 1 | 0 | 1 | 0 | 0 | 0 | 1 | 1 |
| 2012 | 1 | 3 | 3 | 1 | 0 | 0 | 0 | 0 | 0 | 1 | 0 | 2 | 0 |
| 2012 | 1 | 1 | 4 | 2 | 1 | 0 | 0 | 0 | 0 | 0 | 0 | 2 | 1 |
| 2012 | 1 | 2 | 4 | 2 | 1 | 0 | 0 | 0 | 0 | 0 | 2 | 2 | 2 |
| 2012 | 0 | 3 | 0 | 0 | 1 | 0 | 0 | 0 | 0 | 0 | 0 | 2 | 1 |
| 2012 | 1 | 1 | 2 | 1 | 0 | 2 | 0 | 0 | 0 | 1 | 0 | 1 | 0 |
| 2012 | 1 | 2 | 1 | 0 | 1 | 0 | 0 | 1 | 0 | 1 | 0 | 2 | 0 |
| 2012 | 0 | 0 | 0 | 1 | 1 | 3 | 0 | 1 | 0 | 0 | 0 | 2 | 1 |
| 2012 | 0 | 0 | 0 | 1 | 1 | 3 | 0 | 0 | 0 | 0 | 0 | 1 | 0 |
| 2012 | 0 | 0 | 1 | 0 | 1 | 2 | 0 | 0 | 0 | 0 | 0 | 2 | 1 |
| 2012 | 0 | 2 | 1 | 1 | 0 | 1 | 0 | 0 | 0 | 0 | 0 | 2 | 0 |
| 2012 | 0 | 4 | 4 | 2 | 0 | 2 | 0 | 2 | 2 | 0 | 2 | 2 | 2 |
| 2012 | 1 | 3 | 1 | 0 | 1 | 1 | 0 | 2 | 0 | 1 | 0 | 2 | 1 |
| 2012 | 1 | 1 | 0 | 0 | 0 | 2 | 1 | 0 | 0 | 0 | 0 | 2 | 0 |
| 2012 | 1 | 0 | 0 | 2 | 0 | 4 | 0 | 2 | 0 | 0 | 0 | 2 | 0 |
| 2012 | 2 | 1 | 0 | 0 | 2 | 3 | 0 | 0 | 0 | 0 | 0 | 2 | 0 |
| 2012 | 1 | 2 | 0 | 2 | 1 | 2 | 0 | 2 | 1 | 0 | 0 | 2 | 0 |
| 2012 | 0 | 4 | 4 | 1 | 0 | 3 | 0 | 0 | 2 | 2 | 0 | 2 | 1 |
| 2012 | 1 | 3 | 3 | 1 | 0 | 0 | 0 | 0 | 0 | 1 | 0 | 2 | 0 |
| 2012 | 0 | 1 | 2 | 1 | 0 | 2 | 0 | 0 | 0 | 1 | 0 | 2 | 0 |
| 2012 | 0 | 3 | 1 | 1 | 1 | 0 | 0 | 0 | 0 | 0 | 0 | 2 | 1 |
| 2012 | 1 | 1 | 4 | 0 | 0 | 2 | 0 | 2 | 0 | 1 | 2 | 2 | 2 |
| 2012 | 1 | 1 | 0 | 2 | 1 | 0 | 0 | 2 | 0 | 0 | 0 | 2 | 0 |
| 2012 | 0 | 1 | 0 | 1 | 0 | 2 | 0 | 0 | 0 | 0 | 0 | 2 | 0 |
| 2012 | 1 | 2 | 4 | 0 | 0 | 0 | 0 | 0 | 0 | 0 | 0 | 2 | 0 |
| 2012 | 1 | 0 | 2 | 1 | 0 | 1 | 0 | 0 | 0 | 1 | 0 | 1 | 0 |
| 2012 | 0 | 2 | 1 | 0 | 0 | 1 | 0 | 0 | 0 | 0 | 0 | 2 | 0 |
| 2012 | 0 | 4 | 0 | 1 | 1 | 1 | 0 | 0 | 0 | 1 | 0 | 2 | 0 |
| 2012 | 0 | 3 | 4 | 1 | 2 | 0 | 0 | 2 | 0 | 2 | 0 | 2 | 0 |
| 2012 | 0 | 0 | 1 | 1 | 0 | 4 | 1 | 2 | 0 | 0 | 0 | 2 | 0 |
| 2012 | 1 | 2 | 0 | 1 | 0 | 4 | 0 | 2 | 0 | 0 | 0 | 2 | 0 |
| 2012 | 0 | 2 | 1 | 1 | 0 | 2 | 0 | 2 | 0 | 1 | 0 | 2 | 1 |
| 2012 | 1 | 1 | 2 | 0 | 0 | 1 | 0 | 0 | 0 | 1 | 0 | 2 | 0 |
| 2012 | 0 | 2 | 1 | 2 | 1 | 3 | 0 | 2 | 0 | 1 | 0 | 2 | 1 |
| 2012 | 1 | 2 | 0 | 1 | 1 | 3 | 0 | 2 | 0 | 0 | 0 | 2 | 1 |
| 2012 | 0 | 2 | 1 | 0 | 0 | 2 | 0 | 2 | 0 | 1 | 0 | 2 | 0 |
| 2012 | 0 | 0 | 0 | 0 | 0 | 2 | 0 | 2 | 0 | 0 | 0 | 1 | 1 |
| 2012 | 0 | 0 | 0 | 1 | 0 | 2 | 0 | 2 | 0 | 1 | 0 | 1 | 0 |
| 2012 | 1 | 2 | 1 | 1 | 0 | 0 | 0 | 0 | 0 | 1 | 0 | 2 | 0 |
| 2012 | 0 | 3 | 2 | 0 | 0 | 0 | 0 | 0 | 0 | 0 | 0 | 1 | 0 |
| 2012 | 1 | 1 | 1 | 2 | 1 | 0 | 0 | 0 | 0 | 0 | 1 | 2 | 1 |
| 2012 | 0 | 0 | 0 | 1 | 0 | 2 | 0 | 0 | 0 | 1 | 0 | 2 | 1 |
| 2012 | 0 | 1 | 1 | 0 | 0 | 1 | 0 | 0 | 0 | 1 | 0 | 1 | 1 |
| 2012 | 0 | 1 | 4 | 0 | 0 | 2 | 0 | 0 | 0 | 0 | 0 | 2 | 0 |
| 2012 | 1 | 0 | 0 | 1 | 0 | 4 | 0 | 0 | 0 | 0 | 0 | 1 | 1 |
| 2012 | 1 | 3 | 1 | 1 | 0 | 3 | 0 | 0 | 0 | 1 | 0 | 2 | 0 |
| 2012 | 0 | 1 | 0 | 0 | 1 | 0 | 0 | 1 | 0 | 0 | 0 | 2 | 0 |
| 2012 | 0 | 2 | 0 | 0 | 1 | 0 | 0 | 0 | 0 | 1 | 0 | 2 | 0 |
| 2012 | 1 | 0 | 1 | 0 | 2 | 2 | 0 | 0 | 0 | 1 | 0 | 2 | 0 |
| 2012 | 1 | 4 | 0 | 2 | 1 | 0 | 0 | 0 | 0 | 1 | 0 | 2 | 0 |
| 2012 | 1 | 4 | 4 | 1 | 0 | 2 | 0 | 0 | 0 | 0 | 0 | 2 | 0 |
| 2012 | 1 | 1 | 0 | 0 | 0 | 2 | 0 | 0 | 0 | 2 | 1 | 2 | 0 |
| 2012 | 2 | 1 | 1 | 1 | 0 | 0 | 0 | 0 | 0 | 1 | 0 | 2 | 0 |
| 2012 | 0 | 1 | 0 | 1 | 0 | 2 | 0 | 2 | 0 | 0 | 0 | 2 | 0 |
| 2012 | 1 | 0 | 4 | 2 | 0 | 1 | 0 | 2 | 0 | 2 | 0 | 2 | 0 |
| 2012 | 1 | 1 | 1 | 1 | 0 | 3 | 0 | 0 | 0 | 0 | 0 | 2 | 0 |
| 2012 | 0 | 2 | 1 | 0 | 0 | 2 | 0 | 0 | 0 | 1 | 0 | 2 | 0 |
| 2012 | 0 | 0 | 2 | 1 | 0 | 2 | 0 | 0 | 0 | 1 | 0 | 2 | 0 |
| 2012 | 1 | 2 | 0 | 2 | 1 | 0 | 0 | 2 | 0 | 0 | 0 | 2 | 0 |
| 2012 | 0 | 1 | 4 | 1 | 0 | 4 | 0 | 2 | 0 | 0 | 0 | 2 | 0 |
| 2012 | 0 | 1 | 0 | 0 | 0 | 4 | 1 | 2 | 0 | 0 | 0 | 1 | 0 |
| 2012 | 1 | 2 | 1 | 1 | 1 | 5 | 0 | 0 | 0 | 0 | 0 | 2 | 0 |
| 2012 | 1 | 2 | 4 | 0 | 1 | 1 | 0 | 2 | 0 | 0 | 0 | 2 | 0 |
| 2012 | 0 | 2 | 1 | 2 | 0 | 1 | 0 | 2 | 0 | 1 | 0 | 2 | 0 |
| 2012 | 1 | 0 | 0 | 1 | 0 | 3 | 0 | 0 | 0 | 0 | 0 | 2 | 0 |
| 2012 | 1 | 4 | 0 | 0 | 2 | 2 | 0 | 0 | 2 | 2 | 0 | 1 | 1 |
| 2012 | 0 | 0 | 0 | 0 | 0 | 0 | 0 | 1 | 0 | 0 | 0 | 2 | 0 |
| 2012 | 0 | 1 | 0 | 0 | 1 | 0 | 0 | 1 | 0 | 0 | 0 | 1 | 0 |
| 2012 | 0 | 1 | 1 | 1 | 0 | 3 | 0 | 2 | 0 | 0 | 0 | 1 | 0 |
| 2012 | 1 | 2 | 1 | 1 | 1 | 0 | 0 | 0 | 0 | 0 | 0 | 2 | 1 |
| 2012 | 1 | 2 | 4 | 2 | 0 | 3 | 0 | 1 | 0 | 0 | 0 | 2 | 0 |
| 2012 | 0 | 1 | 1 | 0 | 0 | 1 | 0 | 1 | 0 | 1 | 0 | 1 | 0 |
| 2012 | 0 | 2 | 1 | 0 | 0 | 2 | 0 | 0 | 0 | 1 | 0 | 2 | 1 |
| 2012 | 1 | 3 | 0 | 1 | 1 | 0 | 0 | 0 | 0 | 0 | 0 | 2 | 1 |
| 2012 | 0 | 0 | 0 | 1 | 0 | 3 | 0 | 2 | 0 | 0 | 0 | 2 | 0 |
| 2012 | 0 | 1 | 2 | 2 | 0 | 2 | 0 | 0 | 0 | 1 | 0 | 2 | 1 |
| 2012 | 0 | 1 | 2 | 1 | 0 | 0 | 0 | 0 | 0 | 0 | 0 | 2 | 0 |
| 2012 | 0 | 0 | 0 | 0 | 0 | 2 | 0 | 2 | 0 | 1 | 0 | 2 | 0 |
| 2012 | 0 | 2 | 0 | 0 | 1 | 0 | 0 | 0 | 0 | 1 | 0 | 2 | 0 |
| 2012 | 0 | 2 | 0 | 0 | 1 | 0 | 0 | 0 | 0 | 0 | 0 | 2 | 0 |
| 2012 | 0 | 1 | 0 | 0 | 2 | 0 | 0 | 2 | 0 | 0 | 0 | 1 | 0 |
| 2012 | 0 | 4 | 2 | 0 | 0 | 0 | 0 | 0 | 0 | 1 | 0 | 1 | 0 |
| 2012 | 1 | 0 | 1 | 0 | 0 | 1 | 0 | 2 | 0 | 1 | 0 | 1 | 0 |
| 2012 | 0 | 1 | 1 | 0 | 0 | 0 | 0 | 0 | 0 | 1 | 0 | 2 | 0 |
| 2012 | 0 | 1 | 1 | 2 | 1 | 1 | 0 | 0 | 0 | 1 | 0 | 1 | 0 |
| 2012 | 0 | 3 | 4 | 1 | 0 | 0 | 0 | 0 | 0 | 0 | 0 | 2 | 1 |
| 2012 | 0 | 1 | 0 | 0 | 0 | 0 | 0 | 0 | 0 | 0 | 0 | 1 | 0 |
| 2012 | 1 | 2 | 3 | 1 | 0 | 2 | 0 | 0 | 0 | 1 | 0 | 2 | 0 |
| 2012 | 0 | 3 | 1 | 2 | 0 | 1 | 0 | 0 | 0 | 1 | 0 | 2 | 0 |
| 2012 | 1 | 3 | 2 | 2 | 0 | 2 | 0 | 0 | 2 | 1 | 0 | 2 | 1 |
| 2012 | 1 | 4 | 0 | 0 | 0 | 2 | 0 | 0 | 0 | 0 | 0 | 2 | 0 |
| 2012 | 1 | 2 | 4 | 0 | 0 | 2 | 0 | 2 | 0 | 0 | 0 | 2 | 0 |
| 2012 | 0 | 2 | 2 | 0 | 0 | 1 | 0 | 0 | 0 | 1 | 0 | 2 | 1 |
| 2012 | 1 | 2 | 2 | 0 | 0 | 2 | 0 | 0 | 0 | 0 | 0 | 2 | 1 |
| 2012 | 1 | 3 | 0 | 0 | 1 | 1 | 0 | 2 | 0 | 0 | 0 | 2 | 0 |
| 2012 | 0 | 1 | 2 | 0 | 0 | 2 | 0 | 0 | 0 | 0 | 0 | 2 | 0 |
| 2012 | 1 | 2 | 0 | 0 | 1 | 0 | 0 | 2 | 1 | 0 | 0 | 2 | 1 |
| 2012 | 1 | 4 | 0 | 2 | 1 | 3 | 0 | 2 | 0 | 1 | 2 | 2 | 2 |
| 2012 | 1 | 1 | 2 | 1 | 0 | 3 | 0 | 1 | 0 | 1 | 0 | 2 | 0 |
| 2012 | 0 | 2 | 0 | 2 | 1 | 0 | 0 | 2 | 0 | 1 | 0 | 2 | 0 |
| 2012 | 0 | 0 | 4 | 1 | 0 | 1 | 0 | 0 | 0 | 1 | 0 | 2 | 0 |
| 2012 | 1 | 3 | 0 | 1 | 0 | 3 | 0 | 2 | 0 | 0 | 0 | 2 | 0 |
| 2012 | 0 | 1 | 0 | 1 | 0 | 2 | 0 | 2 | 0 | 0 | 0 | 2 | 0 |
| 2012 | 0 | 4 | 0 | 1 | 0 | 2 | 0 | 0 | 0 | 0 | 0 | 2 | 0 |
| 2012 | 0 | 1 | 0 | 0 | 0 | 3 | 0 | 0 | 0 | 1 | 0 | 2 | 0 |
| 2012 | 1 | 4 | 4 | 1 | 0 | 3 | 0 | 2 | 0 | 0 | 0 | 1 | 0 |
| 2012 | 0 | 3 | 0 | 0 | 0 | 2 | 0 | 0 | 0 | 0 | 0 | 2 | 1 |
| 2012 | 0 | 0 | 1 | 0 | 1 | 1 | 0 | 2 | 0 | 0 | 0 | 1 | 0 |
| 2012 | 0 | 2 | 1 | 0 | 0 | 1 | 0 | 2 | 2 | 1 | 0 | 2 | 0 |
| 2012 | 1 | 3 | 3 | 1 | 1 | 0 | 0 | 2 | 0 | 1 | 0 | 2 | 0 |
| 2012 | 1 | 2 | 1 | 0 | 0 | 3 | 0 | 2 | 0 | 1 | 1 | 2 | 0 |
| 2012 | 0 | 1 | 4 | 2 | 1 | 1 | 0 | 2 | 0 | 0 | 0 | 2 | 0 |
| 2012 | 1 | 4 | 1 | 2 | 0 | 1 | 0 | 2 | 2 | 0 | 2 | 2 | 2 |
| 2012 | 0 | 1 | 0 | 1 | 0 | 3 | 0 | 0 | 0 | 0 | 0 | 2 | 0 |
| 2012 | 1 | 2 | 1 | 2 | 0 | 2 | 0 | 2 | 0 | 1 | 0 | 2 | 0 |
| 2012 | 1 | 2 | 1 | 2 | 0 | 2 | 0 | 0 | 0 | 1 | 0 | 2 | 0 |
| 2012 | 0 | 3 | 1 | 1 | 0 | 0 | 0 | 0 | 0 | 1 | 1 | 2 | 0 |
| 2012 | 1 | 1 | 2 | 0 | 0 | 1 | 0 | 0 | 0 | 0 | 1 | 2 | 0 |
| 2012 | 1 | 2 | 2 | 2 | 0 | 1 | 1 | 0 | 0 | 1 | 0 | 2 | 0 |
| 2012 | 0 | 1 | 1 | 0 | 1 | 1 | 0 | 0 | 0 | 1 | 0 | 2 | 0 |
| 2012 | 1 | 1 | 4 | 1 | 0 | 2 | 0 | 0 | 0 | 1 | 0 | 2 | 0 |
| 2012 | 0 | 2 | 1 | 1 | 0 | 4 | 0 | 0 | 0 | 0 | 0 | 1 | 1 |
| 2012 | 0 | 1 | 4 | 0 | 1 | 0 | 0 | 2 | 0 | 1 | 0 | 2 | 0 |
| 2012 | 0 | 1 | 4 | 2 | 0 | 1 | 0 | 2 | 0 | 1 | 2 | 2 | 2 |
| 2012 | 1 | 2 | 4 | 2 | 1 | 0 | 0 | 0 | 0 | 0 | 0 | 2 | 0 |
| 2012 | 1 | 1 | 1 | 1 | 0 | 3 | 0 | 2 | 0 | 1 | 0 | 2 | 0 |
| 2012 | 0 | 2 | 1 | 0 | 1 | 1 | 0 | 0 | 0 | 0 | 0 | 2 | 0 |
| 2012 | 0 | 3 | 1 | 0 | 1 | 0 | 0 | 0 | 0 | 1 | 0 | 2 | 1 |
| 2012 | 0 | 1 | 4 | 2 | 1 | 0 | 0 | 2 | 1 | 0 | 0 | 2 | 1 |
| 2012 | 1 | 2 | 2 | 1 | 0 | 3 | 0 | 0 | 0 | 1 | 0 | 2 | 0 |
| 2012 | 0 | 1 | 4 | 2 | 0 | 1 | 0 | 2 | 0 | 0 | 0 | 2 | 0 |
| 2012 | 0 | 2 | 0 | 1 | 1 | 0 | 0 | 2 | 0 | 1 | 0 | 2 | 1 |
| 2012 | 1 | 2 | 1 | 2 | 1 | 0 | 0 | 0 | 1 | 0 | 0 | 1 | 0 |
| 2012 | 1 | 2 | 1 | 0 | 1 | 1 | 0 | 2 | 0 | 1 | 0 | 1 | 0 |
| 2012 | 1 | 1 | 0 | 0 | 0 | 2 | 1 | 0 | 0 | 0 | 0 | 1 | 0 |
| 2012 | 0 | 1 | 0 | 0 | 0 | 3 | 0 | 0 | 0 | 0 | 0 | 1 | 0 |
| 2012 | 0 | 0 | 4 | 2 | 1 | 0 | 0 | 2 | 1 | 0 | 0 | 2 | 0 |
| 2012 | 1 | 3 | 3 | 1 | 0 | 2 | 0 | 0 | 0 | 1 | 0 | 1 | 0 |
| 2012 | 0 | 3 | 2 | 0 | 0 | 1 | 0 | 0 | 2 | 1 | 0 | 1 | 1 |
| 2012 | 0 | 2 | 1 | 1 | 1 | 0 | 0 | 0 | 0 | 1 | 0 | 2 | 1 |
| 2012 | 0 | 1 | 1 | 1 | 0 | 1 | 0 | 1 | 0 | 1 | 0 | 1 | 1 |
| 2012 | 1 | 1 | 1 | 2 | 0 | 4 | 0 | 0 | 0 | 0 | 0 | 1 | 0 |
| 2012 | 1 | 2 | 4 | 1 | 0 | 3 | 1 | 0 | 0 | 0 | 0 | 2 | 0 |
| 2012 | 0 | 3 | 1 | 0 | 0 | 2 | 0 | 1 | 0 | 0 | 0 | 2 | 0 |
| 2012 | 1 | 1 | 1 | 0 | 0 | 1 | 0 | 0 | 0 | 1 | 0 | 1 | 0 |
| 2012 | 1 | 2 | 1 | 2 | 0 | 2 | 1 | 0 | 0 | 0 | 0 | 1 | 0 |
| 2012 | 1 | 3 | 1 | 1 | 0 | 4 | 0 | 2 | 0 | 0 | 1 | 1 | 0 |
| 2012 | 0 | 3 | 0 | 2 | 1 | 0 | 0 | 2 | 0 | 0 | 0 | 2 | 0 |
| 2012 | 1 | 0 | 0 | 0 | 1 | 3 | 0 | 0 | 0 | 0 | 0 | 1 | 0 |
| 2012 | 0 | 1 | 1 | 0 | 0 | 0 | 0 | 0 | 0 | 0 | 0 | 2 | 0 |
| 2012 | 1 | 4 | 1 | 0 | 0 | 2 | 0 | 0 | 0 | 1 | 0 | 1 | 0 |
| 2012 | 0 | 3 | 4 | 2 | 1 | 2 | 0 | 2 | 0 | 0 | 2 | 2 | 2 |
| 2012 | 0 | 2 | 0 | 0 | 0 | 0 | 0 | 2 | 0 | 1 | 0 | 2 | 1 |
| 2012 | 1 | 1 | 4 | 0 | 0 | 3 | 1 | 0 | 0 | 0 | 0 | 1 | 0 |
| 2012 | 1 | 0 | 0 | 1 | 0 | 4 | 0 | 0 | 0 | 0 | 0 | 1 | 0 |
| 2012 | 1 | 2 | 0 | 1 | 0 | 2 | 0 | 0 | 0 | 1 | 0 | 2 | 0 |
| 2012 | 1 | 1 | 0 | 1 | 1 | 3 | 0 | 2 | 0 | 0 | 0 | 2 | 1 |
| 2012 | 0 | 1 | 0 | 0 | 1 | 2 | 0 | 2 | 0 | 0 | 0 | 1 | 0 |
| 2012 | 0 | 1 | 1 | 2 | 1 | 0 | 0 | 2 | 0 | 1 | 0 | 2 | 0 |
| 2012 | 0 | 1 | 0 | 0 | 1 | 0 | 0 | 2 | 1 | 0 | 0 | 2 | 1 |
| 2012 | 0 | 4 | 1 | 0 | 0 | 2 | 0 | 2 | 0 | 1 | 0 | 2 | 0 |
| 2012 | 1 | 2 | 2 | 0 | 0 | 2 | 0 | 0 | 0 | 1 | 0 | 2 | 0 |
| 2012 | 1 | 1 | 0 | 0 | 0 | 1 | 0 | 2 | 0 | 1 | 0 | 2 | 0 |
| 2012 | 2 | 0 | 0 | 1 | 0 | 0 | 0 | 0 | 0 | 0 | 0 | 2 | 0 |
| 2012 | 1 | 1 | 1 | 2 | 0 | 1 | 0 | 0 | 0 | 1 | 0 | 2 | 1 |
| 2012 | 0 | 2 | 1 | 0 | 1 | 0 | 0 | 0 | 0 | 0 | 0 | 2 | 0 |
| 2012 | 0 | 1 | 1 | 0 | 0 | 2 | 0 | 2 | 0 | 0 | 0 | 2 | 0 |
| 2012 | 0 | 2 | 4 | 0 | 1 | 1 | 0 | 0 | 0 | 0 | 0 | 2 | 1 |
| 2012 | 0 | 2 | 1 | 0 | 1 | 0 | 1 | 1 | 0 | 0 | 0 | 2 | 0 |
| 2012 | 0 | 4 | 0 | 1 | 1 | 3 | 0 | 0 | 0 | 0 | 2 | 2 | 2 |
| 2012 | 0 | 1 | 0 | 2 | 1 | 1 | 0 | 0 | 0 | 0 | 0 | 2 | 0 |
| 2012 | 1 | 1 | 0 | 0 | 2 | 0 | 0 | 0 | 0 | 0 | 0 | 2 | 0 |
| 2012 | 1 | 1 | 0 | 0 | 2 | 3 | 0 | 0 | 0 | 0 | 0 | 2 | 1 |
| 2012 | 0 | 1 | 0 | 1 | 1 | 3 | 0 | 2 | 0 | 0 | 0 | 2 | 1 |
| 2012 | 0 | 4 | 4 | 1 | 1 | 0 | 0 | 0 | 0 | 2 | 0 | 2 | 0 |
| 2012 | 1 | 2 | 0 | 1 | 1 | 0 | 0 | 2 | 0 | 0 | 0 | 2 | 0 |
| 2012 | 0 | 2 | 4 | 0 | 0 | 2 | 0 | 0 | 2 | 2 | 0 | 2 | 0 |
| 2012 | 1 | 2 | 0 | 0 | 1 | 0 | 0 | 0 | 0 | 1 | 0 | 2 | 0 |
| 2012 | 0 | 3 | 4 | 1 | 1 | 0 | 0 | 0 | 1 | 1 | 0 | 2 | 0 |
| 2012 | 1 | 3 | 1 | 1 | 0 | 0 | 0 | 0 | 0 | 1 | 0 | 2 | 0 |
| 2012 | 0 | 2 | 0 | 0 | 0 | 0 | 0 | 0 | 0 | 0 | 0 | 2 | 0 |
| 2012 | 1 | 2 | 1 | 2 | 0 | 2 | 0 | 0 | 0 | 0 | 0 | 1 | 0 |
| 2012 | 0 | 0 | 1 | 1 | 0 | 1 | 0 | 2 | 0 | 1 | 0 | 2 | 0 |
| 2012 | 1 | 4 | 4 | 1 | 0 | 1 | 0 | 0 | 0 | 0 | 0 | 2 | 0 |
| 2012 | 0 | 1 | 1 | 0 | 0 | 1 | 0 | 0 | 0 | 1 | 0 | 1 | 0 |
| 2012 | 1 | 3 | 0 | 2 | 0 | 0 | 0 | 2 | 0 | 1 | 0 | 2 | 0 |
| 2012 | 1 | 2 | 4 | 2 | 0 | 4 | 0 | 0 | 2 | 2 | 1 | 2 | 0 |
| 2012 | 0 | 1 | 0 | 2 | 0 | 4 | 0 | 2 | 2 | 0 | 1 | 2 | 0 |
| 2012 | 1 | 2 | 3 | 2 | 1 | 1 | 0 | 0 | 0 | 1 | 1 | 2 | 1 |
| 2012 | 0 | 1 | 1 | 1 | 0 | 2 | 0 | 0 | 0 | 1 | 2 | 2 | 2 |
| 2012 | 0 | 3 | 1 | 0 | 0 | 2 | 0 | 2 | 0 | 0 | 0 | 2 | 1 |
| 2012 | 0 | 1 | 1 | 0 | 0 | 0 | 0 | 0 | 0 | 0 | 0 | 2 | 0 |
| 2012 | 0 | 4 | 1 | 0 | 0 | 2 | 0 | 0 | 0 | 1 | 0 | 2 | 0 |
| 2012 | 0 | 0 | 1 | 0 | 1 | 1 | 0 | 0 | 0 | 0 | 0 | 2 | 0 |
| 2012 | 0 | 1 | 4 | 2 | 0 | 0 | 0 | 2 | 0 | 0 | 0 | 2 | 1 |
| 2012 | 0 | 1 | 0 | 1 | 0 | 2 | 0 | 0 | 0 | 1 | 0 | 2 | 1 |
| 2012 | 1 | 0 | 4 | 1 | 0 | 2 | 0 | 0 | 0 | 0 | 2 | 2 | 2 |
| 2012 | 0 | 2 | 0 | 0 | 0 | 2 | 0 | 2 | 0 | 2 | 0 | 2 | 0 |
| 2012 | 1 | 2 | 1 | 0 | 0 | 3 | 0 | 0 | 0 | 0 | 1 | 1 | 0 |
| 2012 | 1 | 0 | 0 | 2 | 0 | 2 | 0 | 2 | 0 | 0 | 2 | 2 | 2 |
| 2012 | 0 | 0 | 1 | 0 | 0 | 3 | 0 | 0 | 0 | 0 | 0 | 2 | 1 |
| 2012 | 1 | 2 | 0 | 1 | 0 | 3 | 0 | 2 | 0 | 0 | 0 | 2 | 0 |
| 2012 | 0 | 2 | 0 | 0 | 1 | 1 | 0 | 2 | 0 | 1 | 0 | 2 | 0 |
| 2012 | 1 | 3 | 1 | 0 | 0 | 3 | 0 | 0 | 0 | 1 | 0 | 2 | 0 |
| 2012 | 0 | 1 | 0 | 0 | 0 | 2 | 0 | 0 | 0 | 2 | 0 | 1 | 0 |
| 2012 | 1 | 2 | 0 | 2 | 1 | 0 | 0 | 2 | 0 | 0 | 0 | 2 | 0 |
| 2012 | 1 | 4 | 2 | 2 | 0 | 1 | 0 | 0 | 0 | 1 | 0 | 2 | 1 |
| 2012 | 0 | 1 | 0 | 0 | 2 | 2 | 0 | 1 | 0 | 0 | 0 | 1 | 0 |
| 2012 | 1 | 3 | 3 | 1 | 2 | 2 | 0 | 2 | 0 | 1 | 0 | 1 | 1 |
| 2012 | 1 | 2 | 2 | 1 | 2 | 0 | 0 | 0 | 0 | 0 | 0 | 2 | 1 |
| 2012 | 1 | 0 | 1 | 1 | 2 | 2 | 0 | 0 | 0 | 1 | 0 | 1 | 0 |
| 2012 | 0 | 0 | 0 | 1 | 2 | 3 | 0 | 0 | 0 | 0 | 1 | 1 | 0 |
| 2012 | 0 | 0 | 1 | 0 | 2 | 2 | 0 | 0 | 0 | 1 | 0 | 2 | 0 |
| 2012 | 0 | 0 | 1 | 0 | 2 | 1 | 0 | 1 | 0 | 1 | 0 | 2 | 0 |
| 2012 | 0 | 1 | 0 | 1 | 2 | 3 | 1 | 0 | 0 | 1 | 0 | 2 | 0 |
| 2012 | 0 | 1 | 2 | 2 | 2 | 1 | 0 | 2 | 0 | 1 | 0 | 2 | 0 |
| 2012 | 0 | 4 | 0 | 0 | 2 | 3 | 0 | 0 | 0 | 1 | 0 | 2 | 0 |
| 2012 | 1 | 2 | 1 | 0 | 2 | 1 | 0 | 2 | 0 | 1 | 0 | 1 | 1 |
| 2012 | 0 | 1 | 1 | 2 | 0 | 2 | 0 | 0 | 0 | 1 | 0 | 2 | 1 |
| 2012 | 1 | 3 | 1 | 1 | 2 | 3 | 0 | 2 | 0 | 0 | 0 | 2 | 0 |
| 2012 | 0 | 1 | 4 | 1 | 2 | 0 | 0 | 2 | 0 | 1 | 0 | 2 | 1 |
| 2012 | 0 | 0 | 0 | 1 | 2 | 4 | 0 | 0 | 0 | 0 | 0 | 2 | 1 |
| 2012 | 0 | 1 | 0 | 1 | 2 | 2 | 0 | 0 | 0 | 0 | 0 | 1 | 0 |
| 2012 | 1 | 3 | 3 | 0 | 1 | 1 | 0 | 0 | 0 | 1 | 0 | 1 | 1 |
| 2012 | 0 | 0 | 0 | 1 | 1 | 2 | 0 | 2 | 0 | 1 | 0 | 2 | 1 |
| 2012 | 1 | 1 | 0 | 0 | 1 | 1 | 0 | 0 | 0 | 0 | 0 | 1 | 0 |
| 2012 | 0 | 0 | 4 | 2 | 2 | 2 | 0 | 2 | 2 | 1 | 0 | 2 | 0 |
| 2012 | 1 | 4 | 4 | 1 | 2 | 4 | 0 | 0 | 0 | 2 | 0 | 2 | 0 |
| 2012 | 0 | 1 | 0 | 1 | 1 | 0 | 0 | 2 | 0 | 0 | 2 | 2 | 2 |
| 2012 | 0 | 2 | 4 | 2 | 0 | 1 | 0 | 2 | 0 | 1 | 0 | 2 | 0 |
| 2012 | 1 | 2 | 1 | 0 | 1 | 1 | 0 | 2 | 0 | 0 | 0 | 2 | 0 |
| 2012 | 0 | 1 | 4 | 1 | 2 | 1 | 0 | 0 | 2 | 2 | 0 | 2 | 0 |
| 2012 | 1 | 2 | 0 | 0 | 2 | 3 | 0 | 0 | 0 | 0 | 0 | 1 | 1 |
| 2012 | 1 | 1 | 0 | 0 | 2 | 2 | 1 | 0 | 0 | 0 | 0 | 2 | 1 |
| 2012 | 1 | 0 | 4 | 1 | 2 | 4 | 0 | 2 | 0 | 0 | 0 | 2 | 1 |
| 2012 | 0 | 1 | 1 | 2 | 2 | 1 | 0 | 2 | 0 | 1 | 0 | 2 | 1 |
| 2012 | 0 | 2 | 0 | 2 | 2 | 2 | 1 | 2 | 0 | 1 | 0 | 2 | 0 |
| 2012 | 1 | 3 | 0 | 0 | 0 | 0 | 0 | 2 | 0 | 0 | 0 | 2 | 0 |
| 2012 | 0 | 2 | 0 | 1 | 2 | 1 | 0 | 2 | 0 | 1 | 0 | 2 | 0 |
